# Aging and disease-relevant gene products in the neuronal transcriptome of the great pond snail (*Lymnaea stagnalis*): a potential model of aging, age-related memory loss, and neurodegenerative diseases

**DOI:** 10.1007/s10158-020-00242-6

**Published:** 2020-05-24

**Authors:** István Fodor, Péter Urbán, György Kemenes, Joris M. Koene, Zsolt Pirger

**Affiliations:** 1 NAP Adaptive Neuroethology, Department of Experimental Zoology, Balaton Limnological Institute, Centre for Ecological Research, Tihany, 8237 Hungary; 2 Genomics and Bioinformatics Core Facilities, Szentágothai Research Centre, University of Pécs, Pécs, 7624 Hungary; 3 Sussex Neuroscience, School of Life Sciences, University of Sussex, Brighton, BN1 9QG UK; 4 Department of Ecological Science, Faculty of Science, Vrije Universiteit, Amsterdam, The Netherlands

**Keywords:** Mollusc, *Lymnaea stagnalis*, cDNA sequencing, Aging, Neurodegenerative diseases

## Abstract

Modelling of human aging, age-related memory loss, and neurodegenerative diseases has developed into a progressive area in invertebrate neuroscience. Gold standard molluscan neuroscience models such as the sea hare (*Aplysia californica*) and the great pond snail (*Lymnaea stagnalis*) have proven to be attractive alternatives for studying these processes. Until now, *A. californica* has been the workhorse due to the enormous set of publicly available transcriptome and genome data. However, with growing sequence data, *L. stagnalis* has started to catch up with *A. californica* in this respect. To contribute to this and inspire researchers to use molluscan species for modelling normal biological aging and/or neurodegenerative diseases, we sequenced the whole transcriptome of the central nervous system of *L. stagnalis* and screened for the evolutionary conserved homolog sequences involved in aging and neurodegenerative/other diseases. Several relevant molecules were identified, including for example gelsolin, presenilin, huntingtin, Parkinson disease protein 7/Protein deglycase DJ-1, and amyloid precursor protein, thus providing a stable genetic background for *L. stagnalis* in this field. Our study supports the notion that molluscan species are highly suitable for studying molecular, cellular, and circuit mechanisms of the mentioned neurophysiological and neuropathological processes.

**Electronic supplementary material:**

The online version of this article (10.1007/s10158-020-00242-6) contains supplementary material, which is available to authorized users.

## Introduction

Neuroscience research has been using molluscan species since the 1950s, when neuroscientists such as Nobel Prize laurates Alan Hodgkin, Andrew Huxley and Eric Kandel recognized how useful they can be in answering fundamental neurobiological questions. Such attractive and even today frequently used molluscan species are the sea hare (*A. californica*) and the great pond snail (*L. stagnalis*). For a long time, they were used for examining the neuronal processes from molecular signalling through motor pattern generation to behavior, including learning (Crossley et al. 2019; Kandel 2001; Kemenes and Benjamin 2009; Kupfermann and Kandel 1969; Nikitin et al. 2008; Pirger et al. 2010, 2014a; Rivi et al. 2020; Wachtel and Kandel 1967). Their central nervous system (CNS) has a relatively simple organization with a small number of neurons (~ 10,000 in *A. californica* and ~ 25,000 in *L. stagnalis*). These colored, mostly large-sized (~ 50–100 µm) cells are located on the surface of the ganglia, which makes them easily accessible and individually identifiable. These simpler systems are responsible for generating a number of well-defined behaviors (e.g., feeding, locomotion, respiration, learning) generated by similar types of reflexive and central pattern generator networks that also occur in vertebrates (Benjamin and Kemenes 2020; Kemenes and Benjamin 2009; Moroz 2011). Making use of this potential, neuroscientists revealed that these species use numerous evolutionary conserved signalling pathways involved in learning and memory consolidation, providing further evidence for the generality of highly conserved neuronal mechanisms across phylogenetic groups (Kandel 2001; Rivi et al. 2020).

Since the 2000s, owing to the multi-omics coverage, modelling of human aging, age-related memory loss, and neurodegenerative diseases has been in vogue as a dynamically developing topic of invertebrate neuroscience. Compared to arthropods or nematodes, molluscan species seem to be more attractive for investigating these biological processes. This can be attributed to the presented benefits of their CNS and the slower rate of gene evolution in molluscan lineage resulting the presence of more gene homologs associated with human aging and (neurodegenerative) diseases in these species (Moroz 2009; Moroz et al. 2006; Moroz and Kohn 2010; Walters and Moroz 2009). Although *L. stagnalis* has already been used successfully for modelling aging, Parkinson’s and Alzheimer’s diseases (Arundell et al. 2006; de Weerd et al. 2017; Ford et al. 2017; Hermann et al. 2007, 2020; Maasz et al. 2017; Patel et al. 2006; Pirger et al. 2014b; Scutt et al. 2015; Vehovszky et al. 2007; Yeoman and Faragher 2001; Yeoman et al. 2008), due to the enormous set of publicly available transcriptome and genome data *A. californica* was the prevalent model of this field (Choi et al. 2014; Moroz 2011; Moroz et al. 2006; Moroz and Kohn 2010; Shemesh and Spira 2010a, b). For example, an earlier comparative analysis in *A. californica* yielded several homologs to human genes linked to aging and neurodegenerative/other diseases, opening the way for further and deeper investigations (Moroz et al. 2006).

Nowadays, *L. stagnalis* is approaching *A. californica* also in this respect, since more transcriptome datasets (Davison and Blaxter 2005; Feng et al. 2009; Sadamoto et al. 2012) and an unannotated draft genome have already been made available. Furthermore, a collaborative effort is underway to produce an annotated genome (*L. stagnalis* genome sequencing consortium; main partners: M-A Coutellec, Rennes, FR; C Klopp, Génotoul Toulouse FR; A Davison, Nottingham UK; ZP Feng, Toronto CA; JM Koene, Amsterdam, NL; D Jackson, Göttingen, DE). In this study, we have sequenced the whole transcriptome of the CNS of *L. stagnalis* and screened for evolutionarily conserved sequences involved in human aging, age-related memory loss, and neurodegenerative/other diseases. Our analysis has yielded a high number of conserved molecules providing a firm foundation for using *L. stagnalis* in this field.

## Experimental animals, nucleotide sequencing, and bioinformatics

For this study, mature specimens of *L. stagnalis* were obtained from our laboratory-bred stocks (originating from the Amsterdam mass culture). Snails were kept in large holding tanks (100 individuals/tank) containing 10 L oxygenated artificial snail water with low copper content at a constant temperature of 20 °C (± 1.5 °C) on light:dark regime of 12 h:12 h. Specimens were fed on lettuce ad libitum three times a week. All animals used in the experiment originated from the same breeding cohort and were thus all of the same age (5 months old, mature snails). All procedures were performed according to the protocols approved by the Scientific Committee of Animal Experimentation of the Balaton Limnological Institute (VE-I-001/01890-10/2013).

RNA preparation, nucleotide sequencing, and sequence assembly have been performed as reported previously (Fodor et al. 2020); details are presented in the Supplementary information. For verification and sequence correction, the findings were compared with virtual cDNA sequences extracted from the unannotated genomic data (generated by Illumina sequencing) to which we have access as part of the *L. stagnalis* genome consortium (genome publication in preparation). The identified sequences were submitted to the NCBI Nucleotide database. Conserved domain search using NCBI CDD/SPARCLE was performed to check if the key regions are present in the deduced protein sequences.

## Results

Our screening resulted in a high number of evolutionary conserved sequences in *L. stagnalis* involved in human aging, age-related memory loss, and neurodegenerative/other diseases (Table 1, for sequence data see Supplementary Figure 1).

**Table 1:** Identified *L. stagnalis* homologs with NCBI accession numbers to genes involved in human aging, aging-related memory loss, and neurodegenerative/other diseases

*L. stagnalis* findings to genes linked to human aging/disease-related genes	Condition resulted by dysfunction in human	Accession number
Klotho	Aging	MT153186
Major vault 1	Aging	MT153187
Gelsolin	Aging, amyloidosis	MT153188
Huntingtin	Aging, huntington’s disease	MT153189
Fragile X mental retardation protein	Aging, fragile X syndrome	MT153190
Parkinson disease protein 7/protein deglycase DJ-1 (PARK7/DJ-1)	Parkinson’s disease	MT153192
α-secretase (ADAM10)	Alzheimer’s disease	MT153191
Apolipoprotein E (apoE) receptor	Alzheimer’s disease	MT137053
Choline acetyltransferase (ChAT)	Alzheimer’s disease	MT153193
Amyloid precursor protein (APP)	Alzheimer’s disease	MT153194
Presenilin 1 (PSEN1)	Alzheimer’s disease	MT153195
Notch receptor 3	Cerebral arteriopathy	MT153197
Potassium voltage-gated channel subfamily KQT member 2 isoform e (KCNQ2)	Epilepsy	MT153198
Aldehyde dehydrogenase family 3 member A2 isoform 2 (ALDH3A2)	Sjörger-Larsson syndrome	MT153199
Copper-transporting ATPase 2 isoform a (ATP7B)	Wilson disease	MT153200
Ubiquitin-protein ligase E3A (UBE3A)	Angelman syndrome	MT153201

Normal and pathological functions of these molecules in higher organisms are presented in detail in Supplementary Table 1

The characteristic motifs of relevant human homologous sequences could be identified in all of our findings, for example the ADF domains, the Carn_acetyltrans domain, and the DUF3652 domain for gelsolin, ChAT, and huntingtin, respectively (Supplementary Figure 2). Primarily, we found several aging (klotho, major vault 1, gelsolin, huntingtin, and fragile X mental retardation protein) and Alzheimer’s disease (ADAM10, apoE receptor, ChAT, APP, and PSEN1; most of these are also involved in aging) related sequences.

These findings can add valuable molecular information to the earlier mentioned studies utilizing *L. stagnalis* for modelling aging (e.g., Hermann et al. 2020) and Alzheimer’s disease (Ford et al. 2017). Furthermore, since all of these molecules are present also in *A. californica* (Moroz et al. 2006; Moroz and Kohn 2010; for sequence comparison see Supplementary Figure 3), our findings support the concept that molluscs provide a solid genetic background for this kind of modelling. Beside the potential of modelling Alzheimer’s disease, previous studies have demonstrated that in vivo *L. stagnalis* parkinsonian models can mimic several etiological properties of Parkinson’s disease (Maasz et al. 2017; Vehovszky et al. 2007). Moreover, we identified, for the first time in molluscs, the presence of PARK7/DJ-1 protein in *L. stagnalis* and demonstrated that it may have a conserved neuroprotective function (Maasz et al. 2017). The sequence information for PARK7/DJ-1 also has been provided in this study paving the way for further investigations. Finally, some further disease-relevant genes, such as notch 3 receptor, KCNQ2, ALDH3A2, ATP7B, and UBE3A were also identified; all of these also are present *in A. californica*.

## Discussion

Modelling of human aging and diseases requires a high level of conservation among many genetic processes known to be lost in the particularly derived genome of *Drosophila melanogaster* and *Caenorrhabditis elegans*. Furthermore, the long-term investigation of age-related mechanisms is difficult in these species which have extremely short lifecycles. However, the life expectancy of *A. californica* and *L. stagnalis* is about 1 year varying under different raising conditions (Heyland and Moroz 2006; Nakadera et al. 2015), allowing the following of aging-related processes on a larger scale.

Based on our findings, just like *A. californica*, *L. stagnalis* also possesses a high number of evolutionary conserved homolog molecules involved in human biological aging and neurodegenerative/other diseases. The appropriate genetic background, the advantages of simpler CNS, and the relative long lifespan with a well-characterized life cycle make *L. stagnalis* highly suitable for studying molecular, cellular, and circuit mechanisms of aging, age-related memory loss, and neurodegenerative/other diseases. The innovation of CRISPR/Cas9-mediated genome editing approach in molluscan research, allowing the genetic modification of identified key sequences, further supports the high feasibility of this approach (Abe and Kuroda 2019; Perry and Henry 2015).

## Electronic supplementary material

Below is the link to the electronic supplementary material.

Supplementary material 1 (PDF 827 kb)
