# Supplementary material for: Aging and disease-relevant gene products in the neuronal transcriptome of the great pond snail (Lymnaea stagnalis): a potential model of aging, age-related memory loss, and neurodegenerative diseases
Source: Invert Neurosci. 2020 May 24;20(3):9. doi: 10.1007/s10158-020-00242-6 (PMC7246240; doi:10.1007/s10158-020-00242-6)
Supplement: Supplementary file 1 — Supplementary material 1 (PDF 827 kb) [file 10158_2020_242_MOESM1_ESM.pdf]

Supplementary information

[1]István Fodor, [2]Péter Urbán, [3]György Kemenes, [4]Joris M. Koene, and [1]Zsolt Pirger*

[1]NAP Adaptive Neuroethology, Department of Experimental Zoology, Balaton Limnological Institute, Centre for Ecological Research, 8237 Tihany, Hungary

[2]Genomics and Bioinformatics Core Facilities, Szentágothai Research Centre, University of Pécs, 7624 Pécs, Hungary

[3]Sussex Neuroscience, School of Life Sciences, University of Sussex, Brighton, BN1 9QG, UK

[4]Department of Ecological Science, Faculty of Science, Vrije Universiteit, Amsterdam, the Netherlands

*Address correspondence to Dr. Pirger Zsolt, NAP Adaptive Neuroethology, Department of Experimental Zoology, Balaton Limnological Institute, Centre for Ecological Research, 8237, Tihany, Hungary

E-mail: pirger.zsolt@okologia.mta.hu

Details of RNA preparation, nucleotide sequencing, and bioinformatics.

The whole CNS was dissected from the snails (n=15) and homogenized using a TissueLyser LT (QIAGEN) in TRI reagent (#93289, Sigma-Aldrich). RNA was isolated with Direct-zol^{TM} RNA MiniPrep (#R2050, Zymo Research) following the instructions of manufacturer. The RNA was quantified by Qubit BR RNA Kit (#Q10211, ThermoFisher) and the quality was checked on Agilent Bioanalyzer 2100 using RNA 6000 Nano Kit (#5067-1511, Agilent).

Nanopore sequencing was used to identify evolutionary conserved sequences involved aging, aging-related memory loss, and (neurodegenerative) diseases. The library was prepared using cDNA-PCR Kit (#SQK-PCS108, Oxford Nanopore Technologies) according to the description of manufacturer. The sample was sequenced on a MinION device with R9.4.1 flowcells (#FLO-MIN106). Base calling was performed using Guppy v3.2.2 software. Previous findings on relevant sequences of *A. californica* (Moroz et al. 2006; Moroz and Kohn 2010) were used as search queries. Adapters were trimmed with Porechop v0.2.4 (Wick, 2018), moreover sequences with internal adapters, which were indicating chimera reads, were also splitted with Porechop. Reads were assembled with CLC Genomimcs Workbench v12.0.3 software *de novo* pipeline (QIAGEN). Consensus sequence was called and manually corrected also within CLC Genomics Workbench.

Figure S1. Identified nucleotide and deduced protein sequences of *L. stagnalis* homologs of human genes relevant to aging, aging-related memory loss, and neurodegenerative diseases

>Lymnaea stagnalis_klotho_mRNA

```
TTTTTTAATATAATAATAACTATCCACATTGAGGTCTCCCGCGATGGGAGTACTCGAATCGTTGTTGTATGTCGGTAT
CGGAGTCGCAATTATTTCCCAACTCTCCAACCCTGATTTACAGTTGGTTCCGTCAGGTCTACACACTTTCATTGTTTT
CCCAAAGTACAACGCCTTCCATTTGACCATCATTCGACCAGCACATGGTACTAAGTATGGCAGGAGTCATATCCATCG
GACTCGTGCTGGCCTGTCTCACATGTCTGTCTAAGAGTGCCCTCATACTGGACAAGTTCCCGCAGGGCTTCTCATTCG
GCGTGAGCAGCTCGGCTTATCCAACAGAAGGCGCTTGGGACGCCAGTGGTAAGGGGCCGAGTATATGGGACGAGTTCA
CCAAAACTAACGGCAGGATACAAGGGGGCGGCGACGGCAACAAAGCCGCGGATGGATACAATAAAGTAAAAGAAGACG
TGCAGCTGTTGAAATCCCTTGGAGTCGCGCATTACAAGTTTTCTCTGTCCTGGTCTCGTCTGCTGCCAGATGGGACGA
CCGGAAGTGTCAACCAGGCAGGTTTCGCGTACTACAACTCTCTGATTGATGAGCTCATCGCCAATCAAATCACGCCAT
```

TCGTGACGCTGTACCAGTGGGACCTCCCCCAGGCCCTGCAAGAGAAAGGTGGCTGGTCCAATGACGCATCCGCCACGT
GGTTCAAGGACTACGCCAACCTCTGCTTCTCAAGGTTCGGTGACCGGGTCAAACTATGGACTACCATCGATGATCCAG
TCACACAAGCCTACAAGGGATATGAGACTGGTGAACACGCTCCTGGCCTCACGCAGCCTGGGCTTCTGTATAAGGTGG
GTCACAATCTCCTTCTGGCCCACGGAGAGGCCTATCTCCTCTACAAGTCAACATACAAAGCCACACAGAAAGGCGAAG
TAGGTATGACTCTGAGTTCTGATTGGTTTGTATCGAAAACCGGAAGTGCTACAGATGCCACAGCGACAGCTCGGGCCT
TGACCTTCCAGCTCGGCTGGTTTGCTGAGCCTCTCTTCAACGGCGATTACCCACAACTGATGAAAGAGAAAGTTGCGC
TCAAGAGGACGGCACTGGGCGTGGCAGATCAGAAGCTGCCAGAGTTCACTGCCCAGCAGAAGATAACAATCAGAGGAG
CCTTTGATTTTATTGGTATCAGCCATTTCAAGACCAAACTGGTGTCAGAGAAGGCAAGCGCAAACTCAAATCCGGGTT
TTTACAACGACCAAGATGTTTTGATAGAAACAGACAAGTCATACCCCAAGCTTGAATATCGTCCAGAGACCAACCCGG
ACAGTGACAGGCGTCTGATGGGATTCGGACTGAGAGAACTCCTGAACTATCTGACCAACACCTATAGTAAACCTGCCA
TCTATGTGACCCAGAACGGACTCGATACCTGTGGAACTCTTAAAGACCAGAGCAGAATCGAATACATACGAGAGTATA
GTAACAACATTCTACAAGCAATAAAGGCAGGGAGCGACGTCAGAGGCTACTCCCTGTGGTCGCTGGTCGACGGATTCG
ACTGGGATCGTGGCTACACCACCAAGACCGGGCTGTACTATGTGGACTTTGGTCAAGACGACAGACCACGCTTCCCCA
GGTCATCGGCGAACTTTTATAGGGCTCTGATAGCAAATAGAGGACTTAATGAGGACCTCAGGTCCTACCGAGCATATC
CTGCCGACCGGGATGAGTTTCACTACGGGACGTTCCCGGAGTACTTCAAATGGGGCGTGGCCACATCTGCCTACCAGG
TTGAAGGGGGATGGAATCTGGATGGGAAAGGTCCAAGCATATGGGACACATTCGCACACAACAATCGACTGGCCAATG
GCGAGACTGGAGACGTTGCTTGCGACAGCTACCACCTGTACCAGGAGGATGTCAACATGTTGAAAGATCTTGAGGTTG
ATTTCTACCGTCTGTCCATCGCCTGGAGCAGGGTCCTACCTGACGGCACTTCCAGGTCTCTCAACCAGAAAGGAGTAG
ACTACTACAACAATGTCATCGATGCACTTCTTCTCAGAAATATCACCCCAATGGTGACACTGTACCACTGGGACCTCC
CACAAGCTCTCGAAGACAAGGGCGGGTTTCGGTCTGACAACATTGTTGACCTGTTCGAGGACTACGCGAGGGTTTGCT
TCGAGCAGTTTGGTGACAGGGTGAAACATTGGATAACGTTCAACGAGGCATTTGTCATCAGTTGGCTGGGTTACGGGA
TCGGTATATTTGCTCCGGGTGTTAAGGACCCTGGTGTGGGCACCTACAGAGTGGCGCACAACATCATCAGGTCACATG
CCCGGGCCTACCATCTTTACAAGAATAATTTCAAAGCCAAATATGGAGGCAGCGTCGGTATCACGTTGGACATTGAAT
GGAAGGAACCACTCACCGACTCTGTGGACGACTCTCTGGCTGCAGATCGAGCCATACAGTTTAAACTTGGATGGTTTG
GTAACGCCTTGTTTGGGGGCTCTGGTGATTACCCGGCGGTGGTGAAACAGTATGTAGCTGAGAAGAGCAGGCTCCAGG
GCCTTTCTGTCAGCAGACTTCCTGAGTTTACTGATGATGAGAAAGCCCTAAACAAGGGTGCGTACGACTTTCTGGGCA
TGAATCATTATACAAGTACTCTCGTAGCCAACAAGCCTCGGCCGGACTCACAGCCCAGCTACGAGCAGGACCAGGACA
TATACACCAGAGCTGACCCATGCTGGCCGAGCTCTGATGTTGATTGGCTGAAGATCAACCCGTGGGGTATCCGCTACA
TCTTGCGATGGGCCAAACAGCACTTCAATAATCCGCCAATCTACATCACTGAGAGTGGACGACCCAGTAAAGAAAGTC
TCGATGACCCAGACAGGATATACTACTACAAGTATTACATTAATGAGCTGCTCAAAGCCATCGACTTGGACAAAGTTG
ATCTCCGCGGCTACACGGCCTGGTCACTGATGGACAACCTGGAATGGGCAAGTGCCTACAACGACAAGTTCGGCTTAT
ACCATGTTGACTTCAGCAGCACCAACCGCACGAGAACCCCCCGCAGTTCCGCCCAGTTTTACCGGCAACTGATCAGAG
ATAATGGATTCCCTAAACCCTGAGCCACGCATGCAGATTTGAAGTTTCCCATTCTAAATCTAATCCATATATTTCAAA
CAAAGAACTACTACAAATGTTTTGTTCCTCTACTTTGGGGATTGAGTGATTTGAGTTTTTAATTTTAATAAATCAAAG
GAAATATAGTAAATCAAGGGAAATAAGCCTCGAACTCCAAGGTGTGTGTAACCCTCCTAAATTCGTTCATTACCTTAC
ATGTCATTGTCTTCAGAATATCCTACTGACTGTCTGTCCCACTGTCTGATTGTCAGACTGACAGACTGTCCTACCCGT
TTAAAACAAAACAAATGAAAAGCATGGACACAGTAAATGTTTTTTTTTTAAATAAAATTATTTTTCGTAAATAACAAA
GAACATTTTTAATTTTAATGCTATTAAATTTTGTTGTTGTTGTTTTTTTTTTTTTACCTTAATAAAAAAGTTCGGATC
AATAACTATTGATTTAAAACATATTAAACGGATAAAGCAACGCATAACATGTAAAATACGATCATTAAAAACCTTTAC
CAGGAAGTCAGTTCGTGGAACAAATAACTGTTTTATTGAATGCATTTTTAGAGATTCCCGAAAGCTGGAATGTGCAAT
AAAGAAATGTCAAATCTAAATAATATTTTTTTTCTTTCAAATATTGAAACAGAAGCTATTTAAATATGTAATAAAATA
TTAAAATTCACTT

>Lymnaea stagnalis_klotho_protein

MVLSMAGVISIGLVLACLTCLSKSALILDKFPQGFSFGVSSSAYPTEGAWDASGKGPSIWDEFTKTNGRIQGGGDGNK
AADGYNKVKEDVQLLKSLGVAHYKFSLSWSRLLPDGTTGSVNQAGFAYYNSLIDELIANQITPFVTLYQWDLPQALQE
KGGWSNDASATWFKDYANLCFSRFGDRVKLWTTIDDPVTQAYKGYETGEHAPGLTQPGLLYKVGHNLLLAHGEAYLLY
KSTYKATQKGEVGMTLSSDWFVSKTGSATDATATARALTFQLGWFAEPLFNGDYPQLMKEKVALKRTALGVADQKLPE
FTAQQKITIRGAFDFIGISHFKTKLVSEKASANSNPGFYNDQDVLIETDKSYPKLEYRPETNPDSDRRLMGFGLRELL
NYLTNTYSKPAIYVTQNGLDTCGTLKDQSRIEYIREYSNNILQAIKAGSDVRGYSLWSLVDGFDWDRGYTTKTGLYYV

DFGQDDRPRFPRSSANFYRALIANRGLNEDLRSYRAYPADRDEFHYGTFPEYFKWGVATSAYQVEGGWNLDGKGPSIW
DTFAHNNRLANGETGDVACDSYHLYQEDVNMLKDLEVDFYRLSIAWSRVLPDGTSRSLNQKGVDYYNNVIDALLLRNI
TPMVTLYHWDLPQALEDKGGFRSDNIVDLFEDYARVCFEQFGDRVKHWITFNEAFVISWLGYGIGIFAPGVKDPGVGT
YRVAHNIIRSHARAYHLYKNNFKAKYGGSVGITLDIEWKEPLTDSVDDSLAADRAIQFKLGWFGNALFGGSGDYPAVV
KQYVAEKSRLQGLSVSRLPEFTDDEKALNKGAYDFLGMNHYTSTLVANKPRPDSQPSYEQDQDIYTRADPCWPSSDVD
WLKINPWGIRYILRWAKQHFNNPPIYITESGRPSKESLDDPDRIYYYKYYINELLKAIDLDKVDLRGYTAWSLMDNLE
WASAYNDKFGLYHVDFSSTNRTRTPRSSAQFYRQLIRDNGFPKP

>Lymnaea stagnalis_major vault 1_mRNA

GTCTGTGTGGCAATATCGATAAATTTGCTGGTGTCAAAACAGGATTTCCCAAATCAAGGTCCGTCATACGCGCAAGTC
GAGACCGGCTGGCTTGGTCATAACTAGTGTATTCTTCAATCAAACACTTTGAGTTTCTGAATGTCCAACTGCAAGACT
CTATAGACTTGACAGTTTATGACACAACACAGAAGAGGAACTCCGCTGTGTGATTTCAAGAACAGCAAGAACTCGTCT
CACGTTTGCAAACAGGAGTTCATGAGCTAACCCCCGTGTTGCTAGCAAAGAACCAATCAGTTTAAACCCAGGCAAGCC
ATTATATTTAACCAACGAAAAAAAAAAAATGGCTGACCCAAGAAAAACCCGTTCCGATGATCCCCATTCCCGATGGGAC
AAGGCCGACAACCCCATCTACCGGATTCCGCCCTATTATTACATCCACGTGATGGACCAGAACAGCAACGTCACGCGG
ATCGAGCTCGGCCCGCAGACGTTCGTCCGCCAGGATAACCAGCGCGTCACGCTGGGCCCCGAGAAGATGATAATCGTC
CCCCCGAGACACTACTGCACCATTGAAAACCCGGTGTGCCGGGACAAGGACGGCAACGTCGTGGTCGACAGACTCGGC
CAGATAAAGCTCAAGCACGCCGAGCTGGAGATCCGACTGGCACAGGACCCGTTCCCGCTGTACCCTGGGGAGATTCTG
AGGCACCCGGTCACCGCTCTCACGGTAGTCCAGTCGAATTCAGCTCTGAAGATCCTGGCCGTCCTGGATTTCAAAGAC
GGTGACGTGGACAGGGCCGCCGGCGATGTCTGGTTGTTTGAGGGTCCCGGCACCTACATTCCGAAGAAGGAGACGGTC
GTGAAGGAGACGGTCAGGGCCACCGTCATCGGCCCCAACCAGGCCATAAAGCTCAGAGCCACGAAAGAGTTCGAGGAC
AGGGATGGGAACCTGCGCGTGACCGGCGAGGAATGGCTGGTCAAGCAGACAGGGGCGTATCTTCCAGGGGTGTTCGAA
GAGGTTGTGGACATCGTGAAGGCTTACGTGTTGACCGAGACAAAGGCCTTGCACGTCAGAGCGTTGAAGTCGTTCAAA
GATGATTTCGGGGTCGAGCGTAAGAACGGGGAGGAATGGCTGATTACAATGGATGACTCGGAGGCACACATCCCTGGT
GTTTACGAAGAGGTCGTTGGCGTGGTCAACATAACGACGCTGACCAACAGGCAGTACGCGGTCATCCTCGATCCCGTC
GGAGCTGATGGAAAGCCGCAGCTGGGACAGAAGAAGCTGGTCAGAGGGGAAAAATCGTTTTTTCTTTTGCCCGGGGAG
AAATTGGAAAAAGGGATTCAAAACATCTACATCCTCGGCGAGGACGAAGGACTCGTGCTTAGAGCCATCGAACAGTTC
AAGGACGGGGTCTCACAGCGAAGCCCGGGAGACAGGTGGATGATAAGGGGGCCGCTGGAGTACGTGCCCCCTGTAGAA
ATAGAGGTGGTCATGAAACGAAAAGCCATTCCTCTGGACCTCAACGAGGGCATCTACGTCAGGGACATCAAGACCGGG
AAGGTCAGGGCCATTACCGGAGCGACCTACATGATCAAAGAAGACGAAGAGCTCTGGGAGAAGGAGCTGCCCGAAACG
GTGGAGGAGCTGATCATCAGTGAAAGGGATCCCAAGTCAGAGAGATACGCCACCAAGGGTCAAGATTCAAAGTCTAAA
ACTCGGGACAAGACTCGCGTCGTAACCTTCAGGGTGCCCCACAACGCAGCAGTCCAGATTTACGATTATAAAGACAAA
AAGTCCCGGGTCGTCTTCGGACCTGAACTGGTCATGCTCGGTCCGGACGAACAGTTCACGGTTCTCAGCCTCTCGGGT
GGTAAACCGAAGAAAAACCACGCCATTAAAGCGCTGTGTCTGCTTCTCGGTCCGGACTTCGCTACAGATATCATCACC
GTGGAGACTGCCGACCATGCCAGGCTGTCCTTGCAGCTGGCGTACAACTGGTACTTTGAAGTTACCGGCAAAACCCCC
GAGGAATCGGCCAAACTGTTCAGCGTGCCGGACTTTGTCGGTGATTCGTGCAAAGCCATCGCCTCTAGAGTTCGCGGC
GCTGTCGCCCAGGTAACGTTTGACGATTTTCACAAGAACTCTGCCAAAATCATCAGGTCATCCGTTTTCGGCTTCAAA
GATGGAAAAGTCGGATCTCATCTGACCTTCTCACAAAACAACCTGGTCATAACGAATGTCGACATCCAATCAGTAGAG
CCCGTTGACCAGCGCACGAGAGACTCTCTTCAAAAATCTGTGCAACTCGCCATTGAGATAACAACGAATTCCCAAGAA
GCCACTGCAAGGCACGAGGCCGAGCGCCTCGAGCAGGAGGCTAAAGGAAGACTCGAACGCCAGAAAATCATGGACGAG
GCAGAGGCCGAGAAGTCCCGGAGAGAGCTCCTGGAGCTTCAGGCGAACAGCGCCGCGGTCGAAAGCACCGGACAGGCC
AAAGCCGAAGCACAGAGCCGCGCTGAGGCGGCCAGGATCGAGGGCGAGGCTGCAGTCCAGCAGGCTAAACTCAAAGCC
GAGGCTCAGACGATAGAGGCCGAATCGGATCTTCAACGCCTGACGCAAGCCAGAACCGCCGAGCTGGCGTACCTGAAG
GAGCAGAACATCATGGAGATCGAGAAGACCACGGAGCTGACCACCATAGAGGTCAACAAGTTTCGTCAGATGGTCGAG
TCGATCGGCAGTGGCACCCTGCTCGCCATGGCGACGGCCGGGCCGGACATGCAGGTGAGGATGCTGCAGGCGCTCGGT
CTCAAGTCCACGCTCATTACTGACGGGACCACCCCTGTGAATCTGTTCAACACTGCCAGCGGGTTGATCGGGGACCTG
GTGCCGTCGAAACGAAGGCGACGAGAGAAGAAAGAAGAAGAGGAGGAATCAGATTCCTTTGCTTAGTTTTGCAGCTGA
GAAGAAGAACGCAGCAGATGTCAGGGCTGAATTGCAATTTTTTTAGAAGGATGAAAGATTAAGAGTTTTAAAAGAGAT
GCTTGTAAGAAAATATATGTGTATCAGTATATTTATGTTTAAATTAATTAATAGAAACGGAGTGAAGATAACAGGTGT

AAAATAAATGACGATAACAGGTGTAAAATGAGAGAAGATAACAGGTGTAAAATGAGCGAACATAACAGGTGTAAAATG
GGTGAAAATAACAGGTGTAAAACCTGCAATAAAATATTTAAAATTGTTCATCTTTTTTTTTCCTACCTACAAATAAAA
TTTAAAACTATGTAACTATAATTCTATAGATCAATATGTTGGCATATATTCTTTTATTCGAAGGCTTCATTCTATTTG
TTTCAGAGATGTTGGAGAAAATTTAATCACTCTGAAGTGAATATTTTCTTTAACTGTGATATATTTTGTGTTATTGAT
ATATGTATTTATTTTTTTAATATGTGTGGCAAATACTTTTTTTGTACAATCATCACACAGTTGAAATCATCTAACTAC
AAATTATTGATTGAACTTAACCTTTTAAAGCTTTAAATTGCGTTTTTCATTTGGCGTCAACAAATCAACTTTAAAGCC
ATCCTTCACTTTGTGCACTATTTTTTCCCTTCAAAGATTTGTTTGTTTTCTATTATTTTTGTACATGTTATATTATTA
TTATTAAGTATTGTTTTGTTTGGAATTGTTTTAAATATGTCCGAAATAAAATTATAATAAATCTCCATTG

>Lymnaea stagnalis_major vault 1_protein

MADPRKTRSDDPHSRWDKADNPIYRIPPYYYIHVMDQNSNVTRIELGPQTFVRQDNQRVTLGPEKMIIVPPRHYCTIE
NPVCRDKDGNVVVDRLGQIKLKHAELEIRLAQDPFPLYPGEILRHPVTALTVVQSNSALKILAVLDFKDGDVDRAAGD
VWLFEGPGTYIPKKETVVKETVRATVIGPNQAIKLRATKEFEDRDGNLRVTGEEWLVKQTGAYLPGVFEEVVDIVKAY
VLTETKALHVRALKSFKDDFGVERKNGEEWLITMDDSEAHIPGVYEEVVGVVNITTLTNRQYAVILDPVGADGKPQLG
QKKLVRGEKSFFLLPGEKLEKGIQNIYILGEDEGLVLRAIEQFKDGVSQRSPGDRWMIRGPLEYVPPVEIEVVMKRKA
IPLDLNEGIYVRDIKTGKVRAITGATYMIKEDEELWEKELPETVEELIISERDPKSERYATKGQDSKSKTRDKTRVVT
FRVPHNAAVQIYDYKDKKSRVVFGPELVMLGPDEQFTVLSLSGGKPKKNHAIKALCLLLGPDFATDIITVETADHARL
SLQLAYNWYFEVTGKTPEESAKLFSVPDFVGDSCKAIASRVRGAVAQVTFDDFHKNSAKIIRSSVFGFKDGKVGSHLT
FSQNNLVITNVDIQSVEPVDQRTRDSLQKSVQLAIEITTNSQEATARHEAERLEQEAKGRLERQKIMDEAEAEKSRRE
LLELQANSAAVESTGQAKAEAQSRAEAARIEGEAAVQQAKLKAEAQTIEAESDLQRLTQARTAELAYLKEQNIMEIEK
TTELTTIEVNKFRQMVESIGSGTLLAMATAGPDMQVRMLQALGLKSTLITDGTTPVNLFNTASGLIGDLVPSKRRRRE
KKEEEEESDSFA

>Lymnaea stagnalis_gelsolin_mRNA

AGCAAGAGTAACAACAATTTGAAAGACTCGATGAGTAGCTTCCCTTGCTACGGTACACCTACGACGAGCTGTGATAGA
ACATTGTTAAATTTAAGCCATTGCCGGTGGTTCTGTTTCCTGTAGGTATTCCCTAGCCCTGACAGAGCTTCCTTGTTG
TGTACCTGAAGTGTGAATCACTTGTGTTGCAGGCACTTTGTGGGACAACATTAGAATCAATCAAGTATAACCATGGCT
GGAAGAGGGCTTGTTAAGGCCAAGAAATATGACTGGAAGGATTCGAACCTTGCCCTGTTTGGCTCAGACTTGGAGAAG
AATGTCAAAAAGGCATCCGCTGCAACTGAAGTGGCCTGGAAAGGGGCTGGGACTCAACCAGGAGTGCAGGTCTGGCGT
ATTGTGCAATTTAAAGTGACACCATGGCCAAAGGAAGATTACGGTAAATTCTTTTCGGGGGATTCATACATTGTTCTG
AATACGTACAAAGAAGAGGGAAATGATCAGCTCCTGTACGATGTCCATTTCTGGATTGGCAAGGAGAGTACTCAGGAT
GAATATGGCACAGCTGCATACAAGACAGTTGAACTGGACACTTTCCTCAACGATGTCCCAGTGCAGCACAGAGAAGTT
CAGGACCATGAATCTGATTTGTTCAAGTCTTACTTCAAGACCATCACCATAATGAAAGGAGGTGCACAAACTGGGTTT
CGTCATGTGGAGGAAGAGAAGTACAAGCCAAGACTGTTCCACTTTAGTGGTCAGAGGAAAAATGTGGTGGTCTCTGAG
GTACCACTTTGTAAAGATCGTATTAAATCTGATGACGTGTTCATTCTTGATCTGGGCAAAAAGATCTATCAGTGGAAT
GGACGTGGCAGCAACAAGGATGAAAGATTCAAGGCTGGTCAGTTCTGCCAACAGTTGGAGTCAGAGAGATCTGGACGG
GCCAAAGCTGATGTGTTGGAGGAAGATACCACAGATCGTTCTCATCTTTTCTATCAGTCTTTGACTGAGGATGACTCC
GATGATGACTCCGAATTTGATGCCAAAGACCTTCAGAAAGAACTCTTCAGATTGTCTGACTCAAGTGGAAACATGACC
TTTAAAGTTGAAAAGAAAGGGACTGTATCCAAATCTGATTTTGACACAAAGGATGTCTTTATTTTGGATGCCAAGAAA
TCTCTTTTTGTTTGGATTGGCCAAGGCACCACGGCTGGAGAGAAGAAACTGGCACTTCAGTATGCTCATGAGTACCTA
CAGAAAACTGACCATCCACTCATTCCTGTTACTTGTCTGAAAGAGGGCCAAGAATCTCGGGACTTTGCAGCAGCCATA
GCAGCCTAATCAATCAGCTTGCCTCTACTGCACAATGAAGCCACTGCTACACAATGAAACCACTACTACAAAAATAAA
CCACTTCATGACAATATTTTCCAAACTACAGTCCACTGGCCTTAATGTTTTGTTCTTTCAATCCAACTCTGATGCCTT
AAATATTCCATAATTGTTAACATTTGTTTATGTAGTGCTCAATGCCAAAGTAACCCCTCAGATTTGTCCAAATGTTTA
CTTTCTTGAAACAATATGGAACTTCTTTTGAAGCTTTTGTGTCTTTGAATAAGCACCAATGACCAATAACTCCCATGT
ATGCAAGTTGTTAATAATCTCCCTTTTCCAAATTTCAAAAGGGCAAATGTTAAACTTTTAAAGATGATTTTTTATTCA
CAGAAATTCCTACTAGTTCTACCGATCAGTTTCCTTAAATTTTTATCTGGATACATTATTTGTGCCAGCTTTTCTTTT

TCTGAACTTTTAATAACTTTTACTGTATCTCTTCCATTCATTATAGTTGCATAACAATTTCGAATAAGAATGGCTCAA
AAAAGTATTTTTGTGTAATTGGTTACACATATAGCATTTATTCTAATTGTTTTTCATAAATATTTGTTCTGGTCATTC
TGGAAAGATTTGTTTCAAAAGCAGCTAAATATAATAAATCAGCTCAATAGATGTCTCAAAAACATAGAACTTCAGAAA
TTGAACAGTATTTTTTTAGTTAAGGTTACAACAAGCTCAGTAGTTATAGCAACTTCAGTTTTAAAATGTTTGCTAAAA
GATGCTCATAACTGTGTTTTTTGTTTTAGATCATTTATATTTATTTAAGATTTTTTAAAATAAATTTTTTGATATTAA
TTGTAATTCATATCTCTTTCAGCAGCTCTGAAATTGGCTGTCTCAGCAAGATAAATTTCAATTTAATCATCATTACTT
AAAAGTAAACTTGTAAGGATTTAATGATTAGTAAGCAAGTGCATTGATAGTCATTGGTAAAACTTACAAACATTTTTT
AGAATTTTTTTGTGTGGTTATTAATGAGCTCACTTACCACTTATCTGAAGGCATGTTACATGTTTCTCCCTTATTTTT
CTTTTTTTTTTACTAGGAGGCTGTTTAACAATTTTAAAAATCTACAGAACATTTATAATAAATCGTTTAAATTAACTA
TTTTATGAAGCTGAAATTAAAATAATAAAAATGTTTTTCTAATCAAATACTACATTAATTATTGTTAAATGAGTTTTT
AAAGTACAGCAGTGGTAGTGAGTTATTCAGTTTTTTTTTAAAAATAAATTAAACAACCACAGAAGTTCAAATAAGAAA
TTGTTATAGTGTCTCCTTTGTCAATAAGTTACGTTACATTATTTGTATTCAAGACATTACCAAGATCTCATTTTTAAA
TAGACATAAATGACACTGAAATACTGACTATTAGTTGAAATAGATAGAAAACTTGTGATGGCTGTAAATGTAAGTTTA
CATCTATACACTGCATTTTTTATATTGCTTTTTTTTTTTCAATTTTGGTACTTTTCTATTTTCTTTTGCAATTAGTTA
TAGTTTGTACACATACAACTCATATTTCTACTCTATACTCCTTTTACATATACAAAAATTTGTTTTTGTATCTTAATG
TAATAATTTCTTTTATACTAAAATTATGTATTTTATTTTTATAAGTTATAATTTCTTGTTTATTTTATTTTTGCTAAA
TTGTCAAATAAATAGGTTCATTTTATTCTT

>Lymnaea stagnalis_gelsolin_protein

MAGRGLVKAKKYDWKDSNLALFGSDLEKNVKKASAATEVAWKGAGTQPGVQVWRIVQFKVTPWPKEDYGKFFSGDSYI
VLNTYKEEGNDQLLYDVHFWIGKESTQDEYGTAAYKTVELDTFLNDVPVQHREVQDHESDLFKSYFKTITIMKGGAQT
GFRHVEEEKYKPRLFHFSGQRKNVVVSEVPLCKDRIKSDDVFILDLGKKIYQWNGRGSNKDERFKAGQFCQQLESERS
GRAKADVLEEDTTDRSHLFYQSLTEDDSDDDSEFDAKDLQKELFRLSDSSGNMTFKVEKKGTVSKSDFDTKDVFILDA
KKSLFVWIGQGTTAGEKKLALQYAHEYLQKTDHPLIPVTCLKEGQESRDFAAAIAA

>Lymnaea stagnalis_huntingtin_mRNA

ATGGCAACTATAGAAAAGCTGATCAAAGCTTTTGAAGGCTTGAAAGTTTTTCAACCAAATGCACAAACAGTTGAAGAT
CCAAAAAAAAAAGATCAAAACTTGCCTACCAAGAAAGACAAGATGCTTCATTGCAATATTGTTGCTGACTGCATGTGC
TCACCTAATATGAGGACTATTGCTGATTTTCCGAAGTTTTTAGGCATTGCCATGGAAAGTTTTCTCACTCTTTGTGAT
GATCCAGAAGCAGATGTCCGCATGGTGGCAGATGAATGTTTGAACAGAACCATAAAAGTTCTCCTGGAAACAAATCTA
GGGAGGCTGCAGGTAGAGCTTTATAAAGAACTGAAGAAAAATGGACCATCAAGAAGTTTAAGAGCTGCACTGTGGAGG
TTTGCTGACATGTGCCATCTGATCAGGCCACAGAAATGCAGACCTTACATAGTGAACTTATTGCCATGTGTAGCCCGC
ATTTGCAGAAGAGAAGAAGAAGCGGTTCAGGACACTTTATCTATGGCAATGCCTAAGATTTGCTCGGCTTTGATGCCC
TTTGCCAATGACACAGAAGTGAAGGCTTTGTTGAAGTCCTTCCTGCCAAACCTTAAATCGAATTCCGCTGTCTGTCGA
AGGATGTCTGCCAGCAGCCTAGCATTAATATGTCAGCATTCCAGGGCGCCCCTGTCTTTTTTTAACTACCTTATTGGT
GTCCTTCTAGAGATGATATTACCCGTGGACATTGATCACGAAGTACCCACTCTCCTTGGTGTGATCCTCTGTCTCCGT
CATTCCATACCCCACCTGGTTCCCTCTAATAATAAAGATCAAGGTCTTAAGGACAGCTTTGGCTACAGAGAATCAGAA
GCCGAACAAGCCATTGGCGAGGAAAAAATGGTTAAGATTTTGCAAGCTTTGCTTCATTACTCAGGTCACAGTGACCAC
AATGTCGTCACTGCTGCTCTTGAATCTTTACAGCAGCTTTTGCGTACTCCACCTCCTATATTGAGAAAAATGTTGCTC
ACACGAGGCAGCATTTCAAGAACATATATATTCATTCATGATTTTGAGGAAGAGGACCAGGCACGAATAGAAAGTGTA
GTGGACTTGACATCAATGTCTGATGATGCAAATTTAGATGAGGATGCGGACTTAGGGTACACCAACAGTCATCCATCT
GCACGAAAGTCTAATACTTCTGGTTTAATGTCAGAATTTGAAGCAAAGTCTGCAACCTCCCAGTCAAGACTAGCTGCA
AGTTTGACTGAAAGTAACGTTGGATATAATGAGCTTGTCAACAATGATGCCATTTTGGTGACAGACGGTACAGACTAC
TCAGGGGTGGAGATTGGAGATTTAAATGAGGAGCGGTCTGAAATGTCTGCTACATCAGGAATGTCCCACAGCGACAGC
ATTGAGACTCTTCAGAGTGTTCGTAGCATCAGTCCCCACCTTGCCTACACCCCACCTGTTCAATTGCTGGGCCATGAC
ATGAATGGAAACCCTCAGATTGTTGTATACCCCGGGGAGGATGCAGCCCCACCCCTGAGTCCTAACCCTGCCCTAGGG
GAACCCCTTGAGCAGATAAATATTGAGCATGAGTCCCTCCAAGAAGATGAGGTACCTCTTCTCTTTTATCTGAGACTT

CTGTGCAAACGGTTTCTGCTCACAGGCGTCACAGATGGCCTGGTCACAGATAAACAGGTCAGGGTCAGTCTGAAGTCA
CTGGCCTTGGGCTGTGTTTCCTGTAGCTTGGCCTTGTGTCCTAGGCTTTTCCTCTTCAAGTTGTGCCCAACAGCAAAC
AATGCAGGTAATGACCAGAACCTACAAGATACTACACTGTATGCCAGTCACCCTGACCACCAGCTGAAAGGTCAGACA
GCTGTTGTTATTGGCAGTTTCATACGGGCAGCTCTTATCGAAGGCAGGGGCAATTTTCACCAGTGGATAGACTCTCAC
AAGCCTCCTGAACAAAGTGTCTTATCACTGGAGGCCCTTTTGAAGATCATCATTAACATTCTAGAGGACGAATCGGCT
GTTGCTGTTCGAGCTGCACTCATGGCATTGCAGATGTGTTTAAGTTACCTGATGGATAGTTGTCATGGTCGTCTGGGC
TTCCGTATTCTTCTGGATCTTCTCATCGTCAAAACCAATCCTTACTGGCTGGTGAAGGTGGAGTTGTTAGAACTTATT
GCTGGTCTCAATTTCAAGGTAATCAGTTACCTGGAGTCTATATCCCCTGACATAGCAAGAGGAGATCACAATTTCCTG
GGCAGGATGTGCTTACAAGAACATATATTTCAAGAGATTGTCATTCAGCTGCTGGGAGATGAGGATCCCAGAGTGCGT
GCAGCAGCATCAGCTGCAATACTTAGAATGGTCCCAACACTGTTTTTGGGTCAGACAGTCCTCAGCAAGACCCTGTC
CTGTCAGTGGCCTCAGATTTAACCCAACATCTGCTCACACCAATCATGAATCCTATCATGGCAGGCCAGCTGCCGCCA
CTGGTCCAGGGCCTAATGAAGCCCTATGCTTTTGACATCTTGCAAGAAATTGATCCAAGTACAGAAAGTGCTCTGTCA
CGAGTTGTTCAGCAGTTGCTGCATACTTTGTTGATGTCTCAATCTAAATTTGTCACCAGTGGTTGTTGTCTAGCTCTG
TGTAGACTGTCGGAAGAATACCTTGTCACACAGTATGCGTCATCTTGGAGTTGTGGTCCAGCCAAGCCTATAGCTTCA
AAGGAAAGAGTGGAGAAGCTGGGGATGAGAAGACCTCCCAGTAGATCTCTCAGTGCTTCTAGCATGGATGAGTTGACA
TCAGCCAGTGGTGGGGGACCCCTGCCCATTGTCTTGTCTCTAATGTTGTCATCACAGGCAGGTCTTGAACTCACCACC
CATCAGGATCTACTTGAACTTGCTGGGAATCTAGTGTGTGGTGCGGCTTACAAGAATCTACGCCCAAGTGAGGATACT
GAGAAGCTATCTGGCTCTGGAGATGATGGACATTGGGCAGCTGTGGCAGACAGATTTTTGGTGCCAATGATCGTGCAG
CTATTCACACACACAGCTAGGCTGCTAAATGCCTGCACACATGCTATAGAGGAAACAATGCCTGGTCCACCACAAGTT
AAACCATCTCTACCGTCATTGCCAAATGCTGCCACACTTAGTCCTGTAAGGAGAAAGATGAAGGGAGAGAAGGAGACT
AACCCACCTGGTCCTGGTGCGTCACCTGACCAAAAATCAGGGCAGAAAACCCCTGGGAAAGATCAAAAAGATTCAGAA
AAGGACAGGAACAGAAAAGATGGCATTGGCTCATTCTACAACATTCCACAGTATGTGAAACTCTTTGAAGTTCTCAGA
GGTTCCTACTCAAACTTCAAGACATCTCTAGACCTGACAAGCTCAGACAAGTTCTGCACCATGTTACGGACAACTCTG
ACAGTTTTATCACAGCTTTTAGAAATCGCTACGCTGTACGACGTTGGCAAGGTCACAGATGAAATTCTTGGCTACCTA
AAGGTCACAATGGCACTCGAGCCTACCTGGACGGTACTGTGTGTTCAGCAGTTGCTGAAAGCCTTGTTTGGAACTAAC
ATGGCCAGTCAGTGGGATTCGAACAGCCAATCAGCAACATCACTGACATCAGACCTGGGCGCCCGTGTTGCCGGGGGA
TCAAGTCCCGGCCTCTACTACTACTGTCTCAACAAACCTTATGCTCAGTTGGCCCATTGTCTGGTAGGAGCTGCATGC
AGAGCCACACTACCAGCTGATGAAGCTCATGGCTCCCTCCTCTGGTTGAAACAGAGAGTAGATAGGAAGCTCCCAGCT
ATCCTCAAACCAACCAGCAAAGTTGATAAAGCTGTGATTGGATCTTACATTCGATTATTTGAACCTCTGGTTATTAAA
GCTCTCAAACAGTACACAGTCACGTCAAGTCTCGATTTACAATGCCAAGTTCTGGCCCTGCTGGCTCAGCTGATACAA
CTCAGAGTCAACTACTGCCTTTTGGATTCTGATCAAATCTTTATTGGCTTTGTCATAAAACAGTTTGAGTATATTGAG
GAAGGCCAAATTAGAAACTCAGAGGTGTTGATCCCTCATATTTTCCAGTTTTTAGTTATGTTGTCATATGAGAAGTTC
CATACCAAATCTATAATAGACATGCCTCGTATCATTCACCGCTGTGATGGTATTATGGCCAGTGGACTACAACCTACC
ACGCATGCCATTCCTGCCCTGCGCCCGGTTGTGTATGACCTGTTCCTCCTGAGGGGCACAGTTAAATCAGAGGTGGGG
AAGGATCTGGAGACTCAGAGGGAAGTTGTAGTTTCCATGCTGCTGAGACTTGTACAGTATTACCAGGCACTAGATATG
TTCACCCTAGTGTTGCAACAGTGCCACAGAGAGAGTGAGGAGAGGTGGAAAAGGTTATCACGCCAGGTCATGGATGCC
GTACTACCTGCTCTTGCCAAACAGCAGATAAACTTAGAAAGTCAAGATGCTTTGGATGTCTTACACCGTTTGTTTGAG
TCTGTGTCACCAAGTGTGTTCCGACCAGTTGACTTCCTCCTCAAGACCCTGCTGGCTCCGCCCCATGATGTGAGTGAT
GTTTCAAGTCTTCAGAAATGGTTGTGTCTAGTTCTCATCATCGTCAGGGTTCTGATTTCCCAATCCAAGGAAGAAGTC
ATTTTATCCAGACTAACAGAACTACAGCTCCATGTCTGTCTCATCCGTGACCTCGACAACTCTGGTACATCAGCAGCT
AACTCTGTTGTGGAGTTGCTGAGGAACCTAGCTCCAGAAGAGACTGTTGCTTGGTTTTTACTTCAAGTAATAGGGAAA
TGCTCTGAAATGTTGAATAAAGAAACTTCACTTCTTGGATCAAACTACAATGGTCCAAGCTACTCTACAAGCAATGGC
AATGGTATCATTGTAAGCAGGTGTCATTTCCTGTTGCAGCAGACATTGCACCTGATGCTCTACATTACACACATGTTT
CAATCAGGTAGTCTGTTTCGACGTGTGGCCACTGCAGCCATGGGTCTGCTGCAGCTTGATTCACCAGCTTGTATGTAC
AGTGTTGGTGAGATTAATGAACACATGGTGGGAGTGAGCAGTGTGTGTCCAGCCTTGACCTTACACTGGTGTAATGTG
CTCATCCTGCTCAACTTTGATGACAGAGGTCTGTGGACCCGTGTGGTGCAAAGTCCACCAACATATAAGAGCACAGGA
ACAAGTGTCCGTCACCTGCCTAGATCATCTGAGTGTTGTGGCTTGGAAATGTTAAGAAGAGGAGGACTTATTCTGTTC
TGTGATTATGTGTGTGAAAACTTAAGTGATGCAGAGCACATGACCTGGCTCATAATTAATCATGTCAGTGACCTCATA
CTGCTCTCAAATGAATCCCCTGTGCAAGATTTTATCAGGCAAGTTGCCATTCACCGCAACTCAGCTGCCAGCAGTTTG
TTTATTCAAGCTATTCATGCAAGAGGAGACAGCATCACAAAGCCATCCATGGTAAAGAGGACTCTCAAATGTCTGGAT
GCTATCCATATTTCTCAAAGTGGATCACTGGTGGCTCTGCTGATTGACAAGTTTCTGGGCTGTCATAGGCTTGCTATC
ACCAGGATGACAGACTCTATTGTATGCCAAAGACTGGAGAGTCTTCTAGGGGAAACAGCTGAAGAAATAGCCAAGCAG
CTGCCTAAAGAAGATATAGAAAAGCAGTTACAATTCATGAAGTCAAACGGATTGATACAAAGACATCAACGTTTAGCA

TCACTTCTCAGCAAGCTCTGCATAGCAGCTGGGTCTACAGCACAGATCAAACTTTCTCCAGAGAGATCTCATCCCCTC
TCCGTAGTCCCAGTGGATGTTGCTAAAATCAGCATAGACAAGGAGTTCTACCTATCAATAGTGAAGGAGCAATGTTTC
ATGGCTACGCCCAACACAAGAGAATGTGCTTTTTTACTGCAGAGACTAGACTACCCAGACATACTTTCTATTACTATG
ACAAAAGAATTTAACTTGTCCATTCTGGAAGAGTGCATGTCCCTTGGTGCTTTCCGGTCAGTTCTGCGGTACAACCGT
GATGTTGATCTTGGGTCATTGAGTCCTCAGCCAGCCTCAAATGAGCACACACTTGACCCTCTGTTCCAGGCTTCCCAG
CTGACCTTGTTCCGCCACATCAACAATATGGTCATCCAGCTGCCTCTTCCCCACCAGTGTCTAGTGTTTATTGATTCG
GCTCCAGCCTCTAGTTTACATTATATGGACCGCATTGAAGAGTTATTTACTGATGCCCAGTGGGTGGACAATAATTTC
TGTCTAGCATCTGCCCTGGTCCGTTACATGGTAGCTATCCATCAGTTTCCGTGGAGAGCAGAGCTGCCCCCTGAGTCC
CTGAAAGATGTGGCTAGTTTCGTTGTGCTCTGCGCTGAGCTGATCCACTGGTCTGTAGAACATGACATGCTCCCAGAG
TCTGAGCACATCCAGAACTGCTTGTCTTGTTTGTCTCTACTCCTGCAAGACCCTGCTGTACATGCACTGATGAGTCAG
ACAGAACATGCTACATTTGTCTGTAGTATTGTGGGATTTCTCTACCAACTTTTATGTTCCATGGCAGTACTCCCTGGT
GAAAATGTAGCATGTCTGTTCCAAGATGATCGTAGGGATGATGCAGAAGAAGAGGATGTTTCCTTATCTGCATGTTTG
ATCAGAGCATGTGATGAGATATCAGAGCTGGTCCACTGCCTGCACACACGCCTGGACCCCAACACATCACACGAGCCA
CGACTGCCACAGTTCCTGGCCTCAACTTTCAGAAATATAATCATTGCTGTAGCCAGACTGCCAGCAGTAAACACTTAT
GCCCGTACCCCTCCCTTAGTTTGGAGACTCGGTTGGTCCCCTACACCTGTAGGAGAACTGCGCACCTGTCTACCTCCT
TTACCCGTGGAGTATTTGCAGGAAAAAGATGTTCTCAAGGAGTTTGTGACAAGAATTAGCTCACTGGGTTGGGTCAAC
CGCCAACAGTTTGAGGAGAGCTGGATGAGCTTACTTGGTGTGTTAAATCCAGTCTCTCATACGGGGCATGACCTGTCG
GCTGAAGAGGAGATAGAGCAAGCCCAAGGCATGGTCATTGCTGTTAAAGCCATCACCTCACTTCTACTGCAGTCATCA
ATGGTACCCCATGCAGGGAATCCTTCAAATAGCTACTATGAGACCAGACCAAGAGATAAACCCCTTGCTTTCCTACAC
ACAAGATGCGGTAAAAAGTTGTCTGTGATCAGAGGTCTTATTGAGAAGGAGATTGTCAACTTGTGTGCTGCCAGGCCA
GACAGGTTGGTACCCCAAGCTTACAGTGGCAGTCCATCAGACAAGTCCAGTCCAAACTTATTTGATGGCAACCTGGAA
AGGGAGCTGGGTGTGGAAGATTTCAGTTTAGGCCAGATATCCATTGAGTCAACATGGTCTTTAGTAGGCAGCCTTGAC
ACCAACCTGTCCAACTCAGACACAACTGATTCTCTGGACAGTCCAACCGGTCGTGGGGACATGGCAGCCACCTCTCCA
TCGTCAGCCTCCTCATCGAGCGGGGGTAGGCTCTCCACGGCTAGGTCGGTTCATCACTGCGGTCTGGACATTCATTCC
TGTCTACAGTTCCTCCTGGAGTTGTATGGAGCCTGGCTTCACATAGACAACAACCCTAAGCCTCCACTGATGCTTCTC
AACTCTGTCGTCAAATCTATGGTGTGCCTCTCTGACCTATTCATGGAGCGGGAACAGTTTGAGTTTATGCAGGACATA
CTCTTGGACCTCCTCAAGGGTCATCCTGTGGAAGATGAGCTCATCACACAATATCTCATTGTTGGCATGTGCAAGGCC
ACTGCTATTGTTGGCACGGAAGCACTGATCAGTGAGCGTGTTGTTAAGCTTATTGAGTCTGGACTTAAGAGCACTCAC
CTACCTACCAAGATCTCATCTCTCCATGGGGCCCTGTACTTACTAGAGGGTGGACCCTCGGAACTCAACAGCAGCCTG
CTACCTATTCTTACAGACTTCCTCTCAAAACATCTGGCAATTGTTATATCACAAACGTGTATCATCAGCCAACAGTTT
GTCATCATTATGTGGGCAGTAGCTTTCTACATTATTGAAAATTTTAGTAATGAACTCAAAGACTCAGATTTTACTTCA
AAAACAATTCAGCTCGTGGTGCAGACAGCTTCAGGGAATGAAGAAAATGTTTCTACTTCAGTTTTCTTGACTGTCATG
AAAGGCACAGAGAGGCTGCTGTTGATAGATGTACTGACACAGAGTGACACAGAAACTATCATTAAACTTAGCATGGAC
AGGCTGTGTCTACCAAACCCACAGAGAGCACTGGCAGCATTGGGCCTCATGTTTACATGCATGTATTCAGGTAAATCC
ACTGACCAGTACAGTCCTCAGCCAAGAGAAGAACAAATGTTTGGAGACAGTGGCTTCCAACTGCTCCATCAGGATCCT
GATTCTCTCATTCTTGCCATGGAGAGGGTGACTGTCCTGTTTGACAGAATCAAGAAAGGCTACCCATATGAAGCTAGA
GTGATCACAAGGCTGCTTCCTGCCTTCTTGGCTGATTTCTTCCCTGCTCAAGACATTATGAATAAAGTCATTGGGGAA
TTTCTATCAGCTCATCAACCGTACCCATACCTCATAGCAAAGGTTGTCTTTCAGGTATTCACCAACCTCCATCAACAA
AAACAACAAGGACTCGTCAAAGAGTGGGTCATGTTGAGCTTGTCCAATTTTACCCAGAGGTCACCAGTGTCCATGGCC
ATGTGGAGTCTCACACTGTTCTTCATAAGTGCATCCACAAATGTCTGGCTGAGGGCCCTATTTCCCCATGTTCTTGGT
CGCATGGGCTACATGGAGCCGATGGACAGGCGGCTGTTTTGTTTGTGTGCTGTTGACTTCTACTGTCAGCTGACGGAC
GACGGCCACAAGAGAGCTTTTCTGGCCACCTTCCAGACCATTGCCGCGCCAGACTCTCCATACTCGGACCTGGTGCAG
TGCATCACTATGTTGTAATCTTACATGATACAGGGTTGGTTGACCATGGTTGTATGGAATACATTGGTAGCTTTTGAG
ACAGCAGCTAATATTATTTTTAAAAAATCTTTATGGGTTATAAAAAAAACAAGTGCTCACTGATGTAAGATTCCTG

>Lymnaea stagnalis_huntingtin_protein

MATIEKLIKAFEGLKVFQPNAQTVEDPKKKDQNLPTKKDKMLHCNIVADCMCSPNMRTIADFPKFLGIAMESFLTLCD
DPEADVRMVADECLNRTIKVLLETNLGRLQVELYKELKKNGPSRSLRAALWRFADMCHLIRPQKCRPYIVNLLPCVAR
ICRREEEAVQDTLSMAMPKICSALMPFANDTEVKALLKSFLPNLKSNSAVCRRMSASSLALICQHSRAPLSFFNYLIG
VLLEMILPVDIDHEVPTLLGVILCLRHSIPHLVPSNNKDQGLKDSFGYRESEAEQAIGEEKMVKILQALLHYSGHSDH
NVVTAALESLQQLLRTPPPILRKMLLTRGSISRTYIFIHDFEEEDQARIESVVDLTSMSDDANLDEDADLGYTNSHPS

ARKSNTSGLMSEFEAKSATSQSRLAASLTESNVGYNELVNNDAILVTDGTDYSGVEIGDLNEERSEMSATSGMSHSDS
IETLQSVRSISPHLAYTPPVQLLGHDMNGNPQIVVYPGEDAAPPLSPNPALGEPLEQINIEHESLQEDEVPLLFYLRL
LCKRFLLTGVTDGLVTDKQVRVSLKSLALGCVSCSLALCPRLFLFKLCPTANNAGNDQNLQDTTLYASHPDHQLKGQT
AVVIGSFIRAALIEGRGNFHQWIDSHKPPEQSVLSLEALLKIIINILEDESAVAVRAALMALQMCLSYLMDSCHGRLG
FRILLDLLIVKTNPYWLVKVELLELIAGLNFKVISYLESISPDIARGDHNFLGRMCLQEHIFQEIVIQLLGDEDPRVR
AAASAAILRMVPTLFFGSDSPQQDPVLSVASDLTQHLLTPIMNPIMAGQLPPLVQGLMKPYAFDILQEIDPSTESALS
RVVQQLLHTLLMSQSKFVTSGCCLALCRLSEEYLVTQYASSWSCGPAKPIASKERVEKLGMRRPPSRSLSASSMDELT
SASGGGPLPIVLSLMLSSQAGLELTTHQDLLELAGNLVCGAAYKNLRPSEDTEKLSGSGDDGHWAAVADRFLVPMIVQ
LFTHTARLLNACTHAIEETMPGPPQVKPSLPSLPNAATLSPVRRKMKGEKETNPPGPGASPDQKSGQKTPGKDQKDSE
KDRNRKDGIGSFYNIPQYVKLFEVLRGSYSNFKTSLDLTSSDKFCTMLRTTLTVLSQLLEIATLYDVGKVTDEILGYL
KVTMALEPTWTVLCVQQLLKALFGTNMASQWDSNSQSATSLTSDLGARVAGGSSPGLYYYCLNKPYAQLAHCLVGAAC
RATLPADEAHGSLLWLKQRVDRKLPAILKPTSKVDKAVIGSYIRLFEPLVIKALKQYTVTSSLDLQCQVLALLAQLIQ
LRVNYCLLDSDQIFIGFVIKQFEYIEEGQIRNSEVLIPHIFQFLVMLSYEKFHTKSIIDMPRIIHRCDGIMASGLQPT
THAIPALRPVVYDLFLLRGTVKSEVGKDLETQREVVVSMLLRLVQYYQALDMFTLVLQQCHRESEERWKRLSRQVMDA
VLPALAKQQINLESQDALDVLHRLFESVSPSVFRPVDFLLKTLLAPPHDVSDVSSLQKWLCLVLIIVRVLISQSKEEV
ILSRLTELQLHVCLIRDLDNSGTSAANSVVELLRNLAPEETVAWFLLQVIGKCSEMLNKETSLLGSNYNGPSYSTSNG
NGIIVSRCHFLLQQTLHLMLYITHMFQSGSLFRRVATAAMGLLQLDSPACMYSVGEINEHMVGVSSVCPALTLHWCNV
LILLNFDDRGLWTRVVQSPPTYKSTGTSVRHLPRSSECCGLEMLRRGGLILFCDYVCENLSDAEHMTWLIINHVSDLI
LLSNESPVQDFIRQVAIHRNSAASSLFIQAIHARGDSITKPSMVKRTLKCLDAIHISQSGSLVALLIDKFLGCHRLAI
TRMTDSIVCQRLESLLGETAEEIAKQLPKEDIEKQLQFMKSNGLIQRHQRLASLLSKLCIAAGSTAQIKLSPERSHPL
SVVPVDVAKISIDKEFYLSIVKEQCFMATPNTRECAFLLQRLDYPDILSITMTKEFNLSILEECMSLGAFRSVLRYNR
DVDLGSLSPQPASNEHTLDPLFQASQLTLFRHINNMVIQLPLPHQCLVFIDSAPASSLHYMDRIEELFTDAQWVDNNF
CLASALVRYMVAIHQFPWRAELPPESLKDVASFVVLCAELIHWSVEHDMLPESEHIQNCLSCLSLLLQDPAVHALMSQ
TEHATFVCSIVGFLYQLLCSMAVLPGENVACLFQDDRRDDAEEEDVSLSACLIRACDEISELVHCLHTRLDPNTSHEP
RLPQFLASTFRNIIIAVARLPAVNTYARTPPLVWRLGWSPTPVGELRTCLPPLPVEYLQEKDVLKEFVTRISSLGWVN
RQQFEESWMSLLGVLNPVSHTGHDLSAEEEIEQAQGMVIAVKAITSLLLQSSMVPHAGNPSNSYYETRPRDKPLAFLH
TRCGKKLSVIRGLIEKEIVNLCAARPDRLVPQAYSGSPSDKSSPNLFDGNLERELGVEDFSLGQISIESTWSLVGSLD
TNLSNSDTTDSLDSPTGRGDMAATSPSSASSSSGGRLSTARSVHHCGLDIHSCLQFLLELYGAWLHIDNNPKPPLMLL
NSVVKSMVCLSDLFMEREQFEFMQDILLDLLKGHPVEDELITQYLIVGMCKATAIVGTEALISERVVKLIESGLKSTH
LPTKISSLHGALYLLEGGPSELNSSLLPILTDFLSKHLAIVISQTCIISQQFVIIMWAVAFYIIENFSNELKDSDFTS
KTIQLVVQTASGNEENVSTSVFLTVMKGTERLLLIDVLTQSDTETIIKLSMDRLCLPNPQRALAALGLMFTCMYSGKS
TDQYSPQPREEQMFGDSGFQLLHQDPDSLILAMERVTVLFDRIKKGYPYEARVITRLLPAFLADFFPAQDIMNKVIGE
FLSAHQPYPYLIAKVVFQVFTNLHQQKQQGLVKEWVMLSLSNFTQRSPVSMAMWSLTLFFISASTNVWLRALFPHVLG
RMGYMEPMDRRLFCLCAVDFYCQLTDDGHKRAFLATFQTIAAPDSPYSDLVQCITML

>Lymnaea stagnalis_fragile X mental retardation protein_mRNA

AGCATTCTTTTATTCTTGCCCAAAATGGAGGATCTTTCGGTTGAAGTTGGTGGAAGCAACGGTGTTTATTATAAAGCA
TACCTAAAAAGTTTTTACGAAGATGAAGTTTTGGTTTCATTTGAGAATAACTGGCAAGCAGACAAGAGAGTCAAACTC
ACAAATGTGCGACTGCCGCCAAAGTCTGGAGCCACAAAGCCTGAATTCAGAGAAGATGAGAGAGTTGAGGTATTTGGA
AAAGTGAAGGATGAGGAAGGATTAGCATGGTACCCTGCTAAAATCAAGATGCTGAAAGGAGAATTTGCTGTGGTTGCT
TCTCCCTGGGATGCAAATGATATTTTGCCCCTGGATAAAATCAGATCAGTTAACCACAACCCCCCTATCACAAAAGAG
TCGTTCTTTCAGTTTGTACTAGAAGTGCCTCCTGACTTGAGAGAAGGTTGTCAGGAGGAGTTGGCCATTCAAGAATTC
CGCAAGCACATTGGTGGTGCCATGGTCTCCTATAATCCTGAAGACAAATCACTACATGTACTCAGCACTAGTCCCAGT
GTCATTAAGAGAGCTTCCATGATTGGAGACATGTTTTTAAGAAACATGAGGCAAAAAGTCTTATTGAAGCAACGCACA
GAGGAAGCTGCGAAAAAACTTCAGAGTACAAAAATCAGGTCAGGATACATGGAGGAATTTCAAGTCCGTGATGAACTT
ATGGGCTTAGCTATTGGCACACATGGGGCCAACATACAGCAAGCCCGTAAGGTGGATGGCATTACAGGCATTGAACTA

GATGAAGGATCTTGTACATTCAAAGTCTATGGGGAGACTCAAGAAGCAGTGAAATCAGCTCGAGGCTTATTGGAGTTT
TCTGAAGAAACCTTTCAAGTTCCTAGAGATCTAGTTGCTAAAGTCATTGGCAAAAATGGCAGAAATATCCAAGACATT
GTTGACAAGTCAGGGGTAGTACGAGTGAAGATCGAAGGGGACAACGAACACGAGACAGAAAGAGAAGAGTTTTTTGCA
TCCTTCCAGGGTCAAGTGCCATTTATATTTGTTGGAACTATGGAAAGTATCAGCAATGCAAAGCTTCTTCTTGAGTAT
CACCTGGATCATCTCAAGGAAGTGGAACAGTTGCGTCAAGCCAAATTAGAAATTGATCAACAGCTTAAGTCACTGTCA
GGGCCCCAGCCTGGTTCATACTTTCCCCCACCCAGAGATAGAAGATGGGGATATCCAGAACAATTTGATGACCGAAGA
GGTCGTGGCAGCAGGGGTGGCCGTGGTACTGGCAGAGGCCGTCGGTGGAATACAGATAGGCACGGAGATGATCCATCA
ATGCCTGCTGCTATGGTTGGTGATTGGTCAGCTGAGGTAGATGAAGAAAAAAGGCAAGCTGGTTATTTAACGGACAGT
ATTCTGAGTGGTCGCGGAAGGGCTGGTGGTGCATACAGGCGAGGTTCAAGGGGTGGTGGTATGAGAGGAGGAAGAGGA
GGACTACCACCACGAGGAGCGGGTTATGATGATGATGAATCCCGTGATCCCAGATCTCGACGTCGAATGACAGATGAT
GACGATACAGTACTTGACAATGCTAGTGTAACCAGCCAAGATCAAGACTATGACCAGCAAGACCGGCAAAGGCGACCA
AGGAGGAAGAAAAATAGACCACGAGGCAATGGTGGCCAGGCATCTGGAACTGAGACTGACACCAGTGTCTCTAACTTC
AGAGGTGATAGAAGCAGAGGTCGAGGTGGTCGTGGTGGTGGTTACAACCAACGGGGACATGAATCAGATTCAGGTCGT
GGGGGATACAGGGGAAATGAATCAGATTCAGGGCACCCGCCAAGTGTATCGGCCTCAGGAGTCGGTCGCAGTTCAGTC
AGTCCCGGGATGCAAACGGTCAATGGGGGAGGTGGTGACCGCCCCACTAAGCAAGAACCACCCCCAAAAGATCAGCGG
GACGGACGACCACCTAGAGACACTAGACCAAGAGGGAGCAACAACAACTCCTCTGTCCCACCGAGCGGATCCACACAG
CCACCTAAGCAAATGGTCGGGAACCATCACAGTGGGAGCGATTCGGACTCTAAACTAGCCAAAAGTAAAATGAATAAC
AATAGTGCCAAAGCCAAGGAACATATAGTGAATGGTGCGGAGTGACAGGTATGATGCGTGCTAGCAATTTTTAATGGG
TGAAAGTTCTCATGCCGAGCATTAGTTTCAGTTGAAACCTGAGTTGTTTGTGGTTGTGAGTTACGTAAGGGAATTAAA
TTATTCTTGGTTTGTTAGTTGTTCTTAGTGATGCTGTGCCCAAGTTTCTCCTCTTACTGCTGCCCAAGACAATGTAGA
TTACAATTGTATTATTTGTGTTATGTCAAATATTTTATGAGATGACTTTGGTACAGATGCAGTATTGTGTGTGTTTTG
ACTGTTGGGCACAGCCATAACAGAATGTAAATTTGATAGTTGTTGTGTTGGGTGTTTTAAACCCATTTGACTCTGACA
TTAGGTTGTTAACAATTTGTGTTAATTGACAGTTATAATAGTGCACGAAAGAGCTCTGCCAGCTTTGTATGTGAAAAG
TATTAATTGTATTTAATCATTATCATCCTACTCAATGGTTATTTTTTTTTTTTTAAATGTTCAATTTTTCTATATTATTC
TGTTGCCTTGGAACAAATCTTTCGATTTTTAAAAAACCATAAAACCTGCAAGACAAATTGAGTTCATCAATTTAGATC
AACAGATTGTGAACATTTTAACTTGTGGATCACTGGACAATCGGAATTAAAGGTGTAAGTGTTACTTTTGAGACCTTT
TGTTTAGGATGTCCACTCCTGCTCAAGCTTGGGTTAGCTGAACCACGTTTTAAAACAAAAAAATGAATTGACCCCCCT
CAGCAGGCTAAAACACCAATCTTTAAATATGTCTTGTGCAGTGATTATCTAAGGCAAGCCATTCCTTTTTATTATTAT
TATAGGGGAAACCAAGTTTTCTTTTTGTTTCAAACTGAACACAAGTATCAAAATTTAAAAAAAATATATAAAAATACT
CTACTTTCTGATTTAAACATTTGTTGATGGTCAAGAAAGACAACAAATTCACATGTTTAAATTTCCTGTGAAAATATT
GTTAAAATATTCTCTTGTGCAGTTTTTTTTATTTAACCCAAGTTAAAGTGTTTTTATTGGAAACTGTTGCCTTTTTAA
GGCATAAGTTCAAATTGATTATAGAGGTCACCCCCTTTTTTCTGAAATGTTTGTCATAATAGAAATAATAAATCATTT
GGAAAGATTATTTTTAATCTGTTTGTACATTTGTAAACATGGCTTGCTATGGCCTTCATAAATGATCACTGCATAGCA
GCTTTGCTGTACTCTCTAATTAAATGAATCCTGATATTGAGATAGAAGAACTCAAAATGTGACAAGGCTTACCAAATT
ATGTGTACATGTGCTTTATTATAACTATGACTCCATTAAATCTTTCATATGCAATAGCAGTTTATAATATTTAATTTT
TTTCTTGTTCTTTATTGATAGTAAATTCAATGTTTTAAGTAAAATATAATCTCTTCCCACTCTTTCTCCCTGTCATTT
TTATTATATTTGAAACCAAAACTATTGAAATGTGTAGTAAAAAATTTAAAAATTGGTACTGCATATGAAAATTAATTT
TGTGACATAATTGTGCATTTTCATTGATTCCAGCTTCTTTATTAACTCTGCTAAACAAAATATATAAAAAAAATAATA
AAAAGCTGGAAAAATCACAAGTCTGACCGTTTCCCTGAAAGTGTAGGTACCCTGGCCATGGAGGACTTGGATTGTGAA
ATGTTGCCCAAGTTAGCCCACCACCAAACATGTTTTTATCAGCACGGTCCAGTTGGAATGAGTAGAGATCTCCAGCAG
TGAATGTGCCACACTTTGAGAAAGTGTTAAAAGCTCAATCATCTTTGGATACTGTGAGACCTAAGGAAGACTTGTGTG
TAATTGAACATGGCATGTCTTGCATGATAAGCTTGCCAAATACTTACATACCCCATGTGTATGGCATTTTTTTTTAAT
ATCATGACCATCAAATCCATACACCATCGATGACATGTGAACTAAATTTGCACCAATTAATTATGATGTGTAAAAAAA
AAAAAAAAAAAAAAAAACTTTAAAAAATTATAAAAATCTCATCATAATTAATTGACAACAGATTTAAGAGTTTTTTTGG
AACCTCTCAACCTCTGTTCCAGGGTTATTCGGTGCACAATTCCACGTGGAATATCCCGAGTGAAAGGAACAGATGAAG
TTAAACAATGATGTGATTATTGATGGCCCAGCACATCATTTAAGGCCAGTGTCCAGCAACAGTAGATTGGACTTATTT
TACAATTTTGGTGACTCATCACAGCAGAACTTGGTAAATTTTAAACACAAAATTTATCGGAATACTTCAAATTTAAAA
AAAAAAAAGAAAATTCTACTCCAGTTCAACTAACACCCAGAAATTTTTAAAAAGTATCAATATAAAAGTTGCCAGAGT
TTTTGCTGTAGTGACTCACTCTATAATCGAAAGTCACAAACAGGGATATTGGATGTGTTATCCTCCATGAGTGACCCA
TTTTTTCTCTCTATGTTTTGTTGCACATTTACCTATGTTAGTTAAGTTCTGAGCCTGACAGAAAATACAACTCCCTTC
ATTTGAACAAATATGGGAAACAATTTGTGAAATTTGAATTACTTTTGGCTGGATAATTTTCTTCAGAAGGAGAATAAA
AAATATTTCATTATTTAAACAAGTAAGAAATTTTATTCAAATTAAATGCAGGCTGTTGAACCCTGACTTAGGTAATGT
TATTTTTGTCAAATAACTACTTGAGCCAAAGAGGCAAAAGTACGTTGAACTGCCATGCCAGCAGGAGCTGAGCAGCAG

AACCCTATTACTGCACCAAACAGTTCAAGTTTCTAAACATGATTTTAATGGGATATCATTTTTGTGTTAATACTTTAC
AAAATATGGAACATCTGCCATTTATTAAAAACAGTTTAAAAAAAAAAGGAAATTGAAAGAAATATTGATATCCCAGAA
TTTTAAAAAATATATTTGGGCATTTATTTTTTCTAAAATAAAAAAAAACATAACAATGGCATTGATATATATATTGAT
TTTTTTTGGTGTTGAAAAAACTTCAGTTTTTAAAAAAATATTTTTCTCTTAATCATTGGGAGAATAAAAAGTTACATG
TTCATAACAAAAGGAAGATATATAGACTATTTCTTCTGTTGAAAACATTTTTTTCTATGTACAGAAAAAATGTGAAAGG
TTAAGGTGACTGCAAGCAAATTGCATGCTAACCTTTGGAGATTCTTGCTTTGACATACAAATAATCTGGTCTTTTGGA
CAAGAAATAGGGAAGGTTTAGCTCCCCAATATACAAATGCAATGCTACTTGTGCAATGTGCTTGAGTTATACAAATTT
TTTTGAGATTCAAATCTTTTTTGTAAAAAATATTTTGGCTTGAAAGTAGTGAGGCACAAATTTTTTGTTTTATTCTTT
CCATGTTTAAAAGAAAACGAACTTAAAAATATTTTTCGTCAGGCTGAAAGTCTAATTTCATTCTATAATGTATTTTAA
ATAATGAGGAAATGAAGATTTTATTGTTCAAATTACTTGCACAAGTTAAAGCATCACATGGGACATTCAGCCAACATT
GCAAGTAGACATGACCCTCACATGTGACATTAAGCTGACAATGGAAGTGAATATTGCAATATTACCAGTGAAAATATG
CGATAATTTCTAGTGGAAATACTCATTTCTTTTAAAAGCTGAAATTGACACTTACGTTTCATACTTTTGACATATATT
TTGCTCTGCAAAAATTGTTCATATGGCTGAATTCATATTATGAATGAGACAATAAATTTAATATGACTGTAGTAATAA
GCAGCAAATAATAGATTCTTATTTCCAAGGAAAAATAACAGTATGTGCCAATAGTATATGGCTACGACAACGATAAAT
TGAATTCTTCAACACCATATTGAATGATTTTCTTGCTGCAGAACGGCTGTCAATGTTCACGCATTCCATTGCGCGGCA
TGTGTGGACTATATGTCCAAACTGAAATGTTAGTTGTACAAGCACTGTCGGCTGACATTAAATGGGGTCAAGAATCTC
ACCAGAACAACACATCAGAAGGTTGTGCTATTGCACCAGTAAGGTGTGGCGCTACAAGAAAGGAAGAAAAGTGAAATA
CCATAACATTCTGTGGAGGTTTGATTCTGGATGTTCAGTAAATATATCAAGAATATCTTTTT

>Lymnaea stagnalis_fragile X mental retardation protein_protein

MEDLSVEVGGSNGVYYKAYLKSFYEDEVLVSFENNWQADKRVKLTNVRLPPKSGATKPEFREDERVEVFGKVKDEEGL
AWYPAKIKMLKGEFAVVASPWDANDILPLDKIRSVNHNPPITKESFFQFVLEVPPDLREGCQEELAIQEFRKHIGGAM
VSYNPEDKSLHVLSTSPSVIKRASMIGDMFLRNMRQKVLLKQRTEEAAKKLQSTKIRSGYMEEFQVRDELMGLAIGTH
GANIQQARKVDGITGIELDEGSCTFKVYGETQEAVKSARGLLEFSEETFQVPRDLVAKVIGKNGRNIQDIVDKSGVVR
VKIEGDNEHETEREEFFASFQGQVPFIFVGTMESISNAKLLLEYHLDHLKEVEQLRQAKLEIDQQLKSLSGPQPGSYF
PPPRDRRWGYPEQFDDRRGRGSRGGRGTGRGRRWNTDRHGDDPSMPAAMVGDWSAEVDEEKRQAGYLTDSILSGRGRA
GGAYRRGSRGGGMRGGRGGLPPRGAGYDDDESRDPRSRRRMTDDDDTVLDNASVTSQDQDYDQQDRQRRPRRKKNRPR
GNGGQASGTETDTSVSNFRGDRSRGRGGRGGGYNQRGHESDSGRGGYRGNESDSGHPPSVSASGVGRSSVSPGMQTVN
GGGGDRPTKQEPPPKDQRDGRPPRDTRPRGSNNNSSVPPSGSTQPPKQMVGNHHSGSDSDSKLAKSKMNNNSAKAKEH
IVNGAE

>Lymnaea stagnalis_Parkinson disease protein 7/Protein deglycase DJ-1 (PARK7/DJ-1)_mRNA

CTTGCTATTTATACTGTCCTTGACCCCGTGACAGTTGTTTATGTTTAATCTGTGTTGCAACAAGGCAGGACACAGGCT
GTTTTATCACTCTTCAAGAAAAAATATGGCATCAGTTTCAACTGCATTAGTTTTTCTTGCCGAAGGGGCAGAGGAGAT
GGAAACAGTAATTACTGTAGATGTCCTCCGTCGAGGGGAAGTTGATGTAGTTTTGGCTGGAATTGATGGAGATGGTCC
TGTGAAATGCAGTCGTAATGTGAAATTGGTTCCTGACAAAAGCCTGAGGGATGCTCTGCATAAAGAATATGATGTTCT
TATTTGTCCTGGTGGAGGATTAGGAGCAGAGAATTTGTGCAAATCCAAAGAGGTTGGAAAAGCACTGCAGGAGCAAGA
GAAAAGAGGTGGATTTATTGCTGCTATTTGTGCAGGTCCTACAGCTCTCCTTGCTCACAATGTGGGAAAAGGGAAAAA
AATTACATCTTATCCAAGTATGGCTGATAAACTAAAAGATGCTTACAAATATTCAGAAGACAGAGTAGTCGTTGATGG
AAAATTTATTACAAGTCGTGGGCCTGGCACATGTTTTGAGTTTGCCCTTGCCATCGTGGAACAGTTAAAGGGAAAGGA
AAAGGCTCATGCACTTGTCAAGCCAATGTTGATTAAAATTTAAGACAACTGTTTTTTTCCTCTTCTGTGCTCTGATTC
ATTGAACATGGTACTTGGATTAGGATTCCTCCATATTTACCAAATCAGGGCCGATTGAAGGATATTTTGTTAACTGGG
TGTGAAGACTCACTGTTTAAAGGATAGAAATTAAAATGAACGTAATCTTGATAAGAGGTAATCTTCCTATGTGTGGTA
GAATTTCTGAGCTTATTTTTATACTTACAGCCCATTCCAGCATTCAGACCACATTTTAGCACTGATGTTTAAAGCGAT
TGCCTCTGTGGGAGCGGTCTGCAGGTCAGGTTTCGAAGCAACAGCTGTTACTCTTACTTTAAATATTCTTTTAGAAA

CTAGTAAGAAATTTCCTCAAACTATTAATTCTTTGAAACAAATTCAGGAAGGATTTCTAATTATTGTGCTATGCCAAT
GCATTTTTCAATATTTTTGATGACAAAAAATGTGCCATTTTGAGAAATAATGTTAAAAGTTTATCTCTGATGAAACCA
TGAGAGACCATTAGCTCTTGAGTTTTCTTGGCTGTAGCTTAATTTGAATCTAATAACAAAATCCATTTTTAATAAATG
ATTCCTCTATACCTTT

>Lymnaea stagnalis_Parkinson disease protein 7/Protein deglycase DJ-1 (PARK7/DJ-1)_protein

MFNLCCNKAGHRLFYHSSRKNMASVSTALVFLAEGAEEMETVITVDVLRRGEVDVVLAGIDGDGPVKCSRNVKLVPDK
SLRDALHKEYDVLICPGGGLGAENLCKSKEVGKALQEQEKRGGFIAAICAGPTALLAHNVGKGKKITSYPSMADKLKD
AYKYSEDRVVVDGKFITSRGPGTCFEFALAIVEQLKGKEKAHALVKPMLIKI*

>Lymnaea stagnalis_ alpha-secretase (ADAM10)_mRNA

CAGGTGAGCTTGGCGATCAGCTTCGATACTATGAGACACTTGACCATCTTGATATTAAAGTCAGACGGAAGAGGAGCA
CAGACAGCAATGGCTTTGATGAAAAATATGTCAGTTTCTCTGCATTTAACAGGGAATTCAACCTTGTATTAAAACCAG
GTACAAAGGTTTTGACCGCTGATTTTTCTGCCAAGCTTGTCTATTCCGATGGTCGGTCAGCTCCTCTCAATGTAAATC
CCAATGACTTTTTTTCTGGTCACATAACAGGCGACAAGAGCGTCAAGGCAGATGCTTACACAGAAGATGGTGTTTGGG
GTGCTAACATCTATGATGAGAATGATACTATCACACTAGAACCTGCGTGGCGCCACCTGCCTTCACCGAACAATCACA
CAATGATAGTCTATCGTCATTCGGACGTCAAGTGGGATAACATCTTCCCCAATCTAAACACGAGTCTAAATCATACAG
TTAAAGTCTGTGGTACCCGACATCCTGAAGATGATCCTGACTACACGCCCTACAGTGAGGAGGACATCAAACAGATGG
AGGAGATGTTCAACAAGAAGAAGAGTTCAACCAGACATAAAAGAGATGGCCTGACCCTGGACACTTGCCATGTCATTG
CTGTAGCCGACTATACTTTCTTCAATGGACCTGGTGGGGGATTTCCTCACAGGACGGCTAACTATGTTGTTCAGACGA
TGCAGAAAGTAGACACCATCTTCAGGAAAACTGTGTGGAACCAAGAGTTACAATTGACAGGCCTGGGATTTCAGATAA
AGGAGTTACGTATCCACCCTGAACCCACGGTGACAAACCAAAGTCATTACAATATGAAGATGGACAACTGGCCTGACA
GTAATTTGCTCAGGCAATTTGGGCAAGACAAAGATTTCCACAATTTCTGCTTGGCCCATTTATTTACCCACCGCAAGT
TTGCAGGTGGAGTTCTAGGGCTTGCCTACATAGCTTCAGCGAGAAAATTCGCAAATGGAGGAATCTGCTCCTTAATTC
GAAACAACCCCATAGCTTACAACACAGGATTTAGCTCAACTATGAACACCAAGGGAAACAACCTGCTTTCTCAGGAGG
CTGTGCTGGTGACAACTCACGGCCACAATTGGGGGTCCGAGCATGACGCAGAGACCAGCGAGTGCGCGCCCAACTCTT
TCAACAAGGGGAGGTACATCATGTATCCATACGCTGTCAGTGGTTACGAGGAAAATAACCATGTGTTTTCCCCCTGCA
GTAAGAGATATGTCTCCGCAGTCATCATGGCCAGGTCTGGCTCATGTTTTAAAGGTAAGAAATATACCTGGTACACGG
CCAATGTGCCGATGTGTGGGAACGGCATTGTGGACAAGAATGAGGACTGTGACAGCGGCGGGTTGGGACTCAGCGGAC
AGGACCCCTGCTGCTCTGCGACCTGCAAACTGAATCCAGGATCTGTCTGTAGCTCCACCAATTATGAGTGCTGCCAAA
ACTGTAATCTGGCACCCAGAGGAACTGTGTGTCGTGGCAAGAGCAAGGAACTCTGCCAGGAGGAGGCAGAGTGCTCTG
GGTACAGCCTGGACTGCCCAGGATCTAAACCAATAGCTGATGAAACAGCGACTGTTTGTATGGATGAAGGCCTGTGTA
AAGGTGGCAAATGTTTGGGCTATTGTGAAAGACACAAGCCCAACTCTAGACCATGTCGCTGTACTGACACAGGCAAAG
AATGTTTCCGATGCTGCAGACCTGAGAACGGCACGTGTGAGAGCGTCAGTGATGATTTCCTGGCAGACGGCCGGCCGT
GCTCGTTTGGTTATTGTGACAAAGGGCGGTGCCAGAGGGGTAAGGCCAACATGATCCAGAGGCTGTTCTCATTCATAG
AACAGTTGGACTCCAGCACTGTAGTGGCCTTCATGAAGAGTAACATTGTTGGGACCATCGTTGTCTTCTCACTGATCA
TCTGGGTGCCTGTCAGTTGGATCGTCAGCTGTATTGACAAACGTCGTGAAAAGAAGAGCAAGAAGTTCCAGGAGCATA
TGTGGAGTAACGTCCTCTCCCACAAACTCAAGAACTACACCATACGAGATTCCACCCAACGACATG

>Lymnaea stagnalis_ alpha-secretase (ADAM10)_protein

MIVYRHSDVKWDNIFPNLNTSLNHTVKVCGTRHPEDDPDYTPYSEEDIKQMEEMFNKKKSSTRHKRDGLTLDTCHVIA
VADYTFFNGPGGGFPHRTANYVVQTMQKVDTIFRKTVWNQELQLTGLGFQIKELRIHPEPTVTNQSHYNMKMDNWPDS
NLLRQFGQDKDFHNFCLAHLFTHRKFAGGVLGLAYIASARKFANGGICSLIRNNPIAYNTGFSSTMNTKGNNLLSQEA

VLVTTHGHNWGSEHDAETSECAPNSFNKGRYIMYPYAVSGYEENNHVFSPCSKRYVSAVIMARSGSCFKGKKYTWYTA
NVPMCGNGIVDKNEDCDSGGLGLSGQDPCCSATCKLNPGSVCSSTNYECCQNCNLAPRGTVCRGKSKELCQEEAECSG
YSLDCPGSKPIADETATVCMDEGLCKGGKCLGYCERHKPNSRPCRCTDTGKECFRCCRPENGTCESVSDDFLADGRPC
SFGYCDKGRCQRGKANMIQRLFSFIEQLDSSTVVAFMKSNIVGTIVVFSLIIWVPVSWIVSCIDKRREKKSKKFQEHM
WSNVLSHKLKNYTIRDSTQRH

>Lymnaea stagnalis_ apolipoprotein E (apoE) receptor_mRNA

GATTACCTATGCTCTCAACACAGACATGGCCCAGTTATCATGTGGCAGTCTCCGTGATGTTTATCCAACATGGGAGGT
CAAGACTCAGTATTCCCTGAAAAGTGTCAAGGCTATTGCCAAGGACTGGCTGTCAGGAAATTGGTACTTTGCTGATGA
GTTTAAAGAGATCATTTTTTTCTGTACTTCTGACGGAAGCTATTGCCAGACTGTCCTTACGTCGGGTATCAAGAGGCC
AAAAAGTCTGGCTGTTGATGCAAGCAAAGGATATCTGTTCTATTCGGACTGGTCATCAAATGACCTCGCTCATGTGGG
ACGAATTGACCTGGACGGCTCCAACCCACTCAAGTTGGCCAGCATCAAGATTGTCCACCCCAATGGACTAACTGCTGA
CATTGCCAACTCCCACCTCTACTGGGGAGACTCATTCCTTGACGTCATTGAGAGAGTGGACTACATCGGTCATAAAAG
GATTGTTATAGCCAAGGGCATCGATGTGTTCCATGTGTTTGGTATGAGCATACTACAGAACTACCTCTATGTGGTCAA
CCATCTGAACAACACCATAGTGAGGATCCATCGTTACAACAGCACAGTACCAAACAAGATCATGTTGAAGGCATCCAG
GAAGCCTGGCACCATTAAACTGTTTCACCCGGTGGCACAACCATTTGAGAAGTTTGATGTCTGTTCATGGGCCCAATG
TGATCAGATCTGTGTTCCGGTACCCAATCCAGTAAATGACTCTGTGAAAGCAAGTTGTGTCTGTAAACTTGGCTTTAA
ACAAGCTCCCGATGGAAAGTGCACAAAGCAAGATGCCACTAAGTTTATGCTTCTGACCAATGGTCAGCAAGGAATGAT
TCATTTCATCTCGACTGACTCATCTGAAGTTAAGCGTGACCTCTACCCTCCCATAGCCAATCTGGGTCGACCAACTTG
TGTTGACTTTGACTACCTTGAAGGCTACATTTATTTCTTTGATGTGTCCTCCCACACATTGAGACGAAGGCGGTTTGA
CTCTGATAAGGAGCCAGAAGCTGTGGTTACACAGGGTATCAACTGTGACGGACTAGCTGTGGACTGGAGTGGACGGAA
TATTTACTGCTCAGACAGTGGCCGCAATAAAATTGTAGCCATAAGCCTGCGGAACTTCAGCCACATCTACACTGTCAT
TGACTCTGATAAGTTGAAGGAGGTCATCAACCCAAAAGCTTTGGCTATTGATGCTAAAAATGGGAAATTGTATTGGAC
CGACTGGGTCATCTCACCTGGTGCGGGCAATGCCAGTATCAACTGGGTGTACATGGATGGCAGCAACTGGGCAAGGAT
ACAACACAAAGAGATCCAGTGGCCCAATGGCCTAGTGCTTGACCCTAACACACAGATGCTCTACTGGACCGATGCCTA
CTATGACAGGATTGAGTCCATGTCTATTGATGGGACTCACAGACAGGTTATACTGAACTTTACAAGTAACCTGCATCC
GTTTGGTATCACTAAACATGGGAACAAGCTCTACTGGGCAGAGTCCATGGATGGCAGCCTTATGGAAATTGACTTGGC
CACAAAGAAAGTAACAAACTATCGCAACAGTAGTGCTCCAGTTTTTGATGTCAAGTTGTATGCCAATAACTCCCAGCC
CAATGCAGGGCATCCATGTTCTACAAATAATGGAGGATGTAGCGACTTGTGCCTTCTAACTCCTGGTGGTGGGGCTGC
GTGCAAGTGTGCTGATGGCAGAACTTCCACAAACAACGGTGTCCTCTGTACAGGTAAGTTGACACCTGGTAACATTGC
TCCCAGGAAGTGCCAGGGGCTGCTAGAGTTTGAATGTGCCAATGGGGAGTGCATACACAGCACAAGGACATGTGATGG
AAAGTTTGACTGCACTGACAAGTCTGATGAGGACATGATGAATCACACCTGCCAGGATGAAGGAATGTTTAAATGTAA
TACATCAACATGTCTGTACATGCAGTTCAAATGTGATGGAGAGGTGGATTGTGTCTTTGGAGAGGATGAGAAGAACTG
TTCAGACCACTCCTGCATGGCTGATCACTTCCAGTGTGCCACAAGTAAGCAGTGTGTTCCTTTGACCTGGAAGTGTGA
TGGTGAGAAGGATTGCAGCGATGGCTCCGATGAGGATGAAGCCCATTGCTGTAAAGTGACTTGTAAACCGGAAGAATT
TGCCTGCCTTGATGGCCGCTGTATTAGATATGAATTCAGATGTGACAACGAATACGACTGTATGGATAACTCGGATGA
GCTGGACTGTCAAGAATGGTGCGACCCTGTACGAGAATTTAAATGTATCAACGAGAGTCGGTGCATACCAAAAATTTT
CCAGTGTGATGGAGAAAAGAACTGTCAGGATGGTGCTGATGAAAAGGGCTGTGAAAAACATGAACGTATATGCCACAA
GGATGAATTTTCCTGTTCAGATGGCACCTGTCTCAAGCTGGAGTACAAGTGTGATGGTAGCAATGATTGTTTGGATGG
CTCGGATGAAATCAACTGTTTGAAGAACATGACTGATAAAGTTTGCCACTGGACAGAGTTTAAATGCAGTGATGGGAC
CCAATGTATAGCTTCTGTCTGGCGGTGTGACCAAGAATTTGATTGTAAAGACAAATCTGATGAGAAAGATTGTGCCAA
GTGCCAGTCACCCAACTTTGCCTGCAAGGCCCACCAGACCATGTGTATACCACCAGAAAAACTCTGTGACAACAACAA
CGACTGCCCGGATTACTCTGATGAAGGCAGATTGTGTGAATATGACATGTGCCTTAACAATGATTGTGAAGCCAAGTG
TCACAAATCACCAGATGGATTTGTCTGCTCCTGTCCAGAGAACCAGAAGCTTCGTCCTGACAACAAGACATGTGTTGA
TATCAATTCCTGTGAGAAATGGGGTATCTGCAGTCAGCTCTGTCAGCCAACTTTTCATGGTCACAAATGTTACTGTAG
CCCTGGTTACACGTTACAAGCTGATGGCTATGGCTGCAAACCTATTGACCCAGATCCTGTGTACATCATATTTGCCAA
CCGCCATGAGATTCGACGCTTGAACACCCACGACAAAAGCATGACCCACCTTGTGTCCAATCTTCAGAACGCAATCGC
ACTGGACTTCCACTATAACCAGTCCCTAGTCTTTTGGACGGATGTCAGCAATGACAAGATATACAGAGGAGAAATCAA
TGCCAACTCTGTGACCAAGATAGAACCTATCATAGAGTTTGGTCTGGCCACTACAGAGGGTGTAGCTGTTGACTGGAT
TGCCAACACTATTTACTGGGTTGAGAGCAATCTGGACCAAATTGAGGTGGCCAAACTGGATGGATCAGAACGTGCTAC

ACTAATAGCCGGCAACATGACCAGTCCCAGGGCTATTGTTCTTGACCCTCGAGTTGGGAAACTGTTCTGGACTGACTG
GGATGGCGCCCATCCAAGGATTGAATCATGCTCTATGGCGGGAGAGCCTGAAACACGTACAGTAGTATATGACATTCG
CAATCAGAAAGGAGCGGGTTGGCCCAATGGGTTGGCTGTGGATTATGAAACCAAACGTCTTTATTGGGTTGATGCAAG
ATCAGACTCGATTCACTGCATTACATATGAAGGCAAAGACCACCAACTGATTCTCAAGTCCAATCGAGCCCTGAGCCA
TCCCTTCTCAGTGACCGTATTTGAACACTACATCTACTGGACTGACTGGAGATTCAATACTCTTGTTGTGGCTAACAA
ATACAATGGCAGTGATGTTCATGTGGTTCACAGCACATACCAACAGCCCTTTGATCTGCAAGTATATCACCCCAAACG
TCAACCTCAGATGGCCAACCCTTGTCTGAACTCACCGTGCAGCCATCTTTGTCTCATCGGGGATGGCCTGAAACCAGT
CTGCCGTTGTCCACACAGATATAAACTGTCTGATGACAAACGTGTTTGTGAGAAGGACAATATCTTCTTGTTGTTCAC
CAAGGAGAATGAGATCCGTGGTGTTGATCTAGAGAATGCACACTACAATGTCATACCATCCATTACTGTTCCCTTTGT
GGAGAATGCCACCTCCATTGACTATGATGTCACAGAAGAACGTCTGTACTGGACTGACATGAAGAAAAATGTTATCAC
CAGTGCTTACCTCAACGGGACAGGAATAACCACTGTTATAGACTCAGGCCTGTCCAACCCATCTGGTTTTGCCATAGA
CTGGGTCTCTAAGAACATGTACTTTTCTTCCTACAATGATGTGGAGGGATACATCTCAGTTGCTAAGTTGGATGGGGC
GTATCGCAAAGAAATCTACAGATCTACCTTTGCATCAAAGCCAAACTCTATTGCAATTCATCCTTCAAAAGGTGTCAT
GTTTTGGTCAGATCTTGGAGGTGAACATCATACTATCTGGAAGGCTAACATGGATGGCAAGAAATCAGGAGTTTTTGT
GGAGGTAGTCAAGAAGCCTGCCAGTCTGACCCTAGACATGGTGTACAATCGCTTGTACTGGATCAGCCAGGAGGAGGG
AGCCATCTTCTGGTGTGATGTCTCACTATCTAAATGCAATGCCACCCTGGATCAAAATGTCTCCATGAAAGAACCCAT
CTCCATGACCCTCCAAATGTTTACAGTGTCTGAACAGTCCTATTTTATGTTGACAGATTCAACTCTTACAAAATGTTC
TCTTTTTTTTCAGTTTCAAAATGGTCATAAGATAACAATGAGAGAAGATACCAAGAATGTGTTTGACCTGAGGGTGTA
TGACCCTAGCAGTAGACAAGGAACCACCAATAACTGTTCTGTGAAGAATGGAGGCTGTGAACAGCTGTGCCTGCCAAC
ACCAGACAGAGGTGGTATCGTGTGTGAGTGCACAGTTGGCTACCAAGCTGTAGCAGGTGGAAAATGTCATGGAATTGA
TACATTCATACTGTACACACAAGCCTCAGAGATACAGGGGATGTTACTGGATGCACAGACTCATTCTCCAGCTTTGGC
CTCAATATCAAAGATTTCGAGAGCCACATCCGTTGACTTTCACGCAGACAATGGTCATATTTATTGGGTGGACAGTGA
TCTCCGTCTCATATCTCGCATCAAAAGAGATCTGAGTGGACGAGAGGTTATAGTGTCCCAAGGGATATCTGGCGCTGA
ATCTTTGGCAGTGGACTGGATAGCAGGTAATATTTACTGGACCGACCAGGGTCACAATACCATTGAGGTGATCAGATT
AAATGGAAGTCAGAGGCATGTTGTGTTGCATGAGGACATCGACAAGCCACGCTCTATTGCTGTGCATCCAGCAAAGGG
GTATCTTTACTTTGCCAATGGGGGCGTCAGCCCTAAGATTGTGAGAACCAGACTGGATGGCTCAGAGAGAGTGGACTT
TGTCTCATCCACTGATGCACAGCCTGTGAAAGCTCCCTTTGGTCTGGCGATAGACTTTGATACAGACGACTTGTACTG
GTGTGACAAGGACCTGGATTTCATTGAGAGGGTAACCCCATCTGGACAAAGATTTTCTGTGGTCACTCATAACCTGAC
TGACTGCATGAGTGTAGCTGTGCACAAGGATAGACTTTATTGGGCTGATCTAACCGACCTTCAGGGGTCCATCAAGTA
TGTGAACAAGACCGGACGAGATGTGACAGCAAGAGATATAACCGTCATGAAGAAAAACATTACCAAACTTAAGGACAT
CAAAGTGTTTGATGGCGAGGCACAGATAGGCAGTAATCCATGTGGCGAAAACAATGGTGGCTGTGAAGAATTGTGCTT
ATATCGGGGGAACAGTAACTTCACCTGCGCTTGTTCTTATGGGAGGCTTAAGGAAGATGGCAAGAGCTGTGCGGAACA
CGACTCCTTCTTGCTGTACTCCGAGATAACATCACTAAGAAGCTTGATCCTGGCCAACTCGACTGATCGAAACGCCCC
CAGGCACCCCATCCAGAATGAAACTTACATGAAGAATGTCATCGGCCTGGCTTTTGACTATGCAACGGAGAGGATTTT
CTTTAGTGACATTCAGCAAGGCAACATCCAGGCAGTCTTTTTTAATGGCACAGGGTTTAGAATAATTAAAGAAGGAGT
TGGTTCTGCTGAAGGACTAGCATTTGATCCCCTTCAAAAACATCTGTACTGGACAAGCTACTCGGGCTCCAACATCAA
CCGTATCTCATTTAATGGATTATCACCCACTAAAAATGATATTTTCTATCAGTTGGATCATACTGATCACCCCAGGGA
TATTGTTGTCAACTCCTGCATTAGGCGTATATTCTGGACCAATTGGAGTGACCGTCGGCCATCCATTCAAACATCCTC
CTATGACAGCGATGGCAGTACAGACAGCTCCGAGTCTATCATCACCGAGGGCATAAGGACACCCAACGGTCTGACCAT
TGACCACAAAGCTCAGAAACTGTACTGGTCCGATGCAAGATTGGACAAGATAGAGAGATGTGACTTTGATGGAAGTAA
TAGATTTATTGTGGTCACATCTATTCCCGAACATTCCTTTGGTCTTGCTGTGTATGGGGATTTCCTCTACTGGACTGA
CTGGGTCGTACGTGCGGTAGTACGTGCCAACAAGTATGATGGCAGCCGTACAACTTTTCTAAAGAAGAATATCAACAG
ACAGCCCATGGGAATCATTGCAGTTGCTAATGATACAGATGACTGCATGCTGAATCCTTGTTTTGAAAATGCTTTTGG
CTGTGCTGAGATCTGTGTGGTCAGTGTTAAAGGTGATGCCATGTGTCAGTGTGGTCCAGGGAAAAAACTTCTGTCAGA
TGGAAAAAGATGTGTTTCGAAAGATCTTGAAAATTGTGATGGCGAAGATTTTATTTGTGAAGACAACAAACTTTGTAT
TCCCTTCAACAAAACCTGTAATGATATCCCCGACTGTCTGGATGCCAGTGATGAGTCCAACCACTACTGCAGTACACG
TCATTGTCCCCAACACTGGTACCAGTGTCAGAACAGTTCTCGCTGTATTATGAACAGTAGGATCTGTGATGGTCGCAA
TGACTGCGGGGATGGGTCAGATGAACTCAACTGCCCTTGTGCCAAAAATGAGTTCCGCTGCAACAATGGCATGTGCAT
CCTGGCTAAATACAAATGTGACTTTGACAGTGACTGCCCAGATCTGTCAGATGAGATTGGTTGCAGTAAGACCTGTGA
AGATCTGGGCATACATGGCATCCAGCACATTGACCTGGTCTCCTGTAACACTACATCCATGTGCATCTACCCTAACTG
GATCTGTGATGGCAGCAATGACTGCTGGGACAATAATGATGAGGTCAACTGTGATGTATTAAGTGGATGTACAGAATT
TGCCTTCACCTGTGATGATGGCAAGTGCATATTGCCTCAGTGGAAGTGTGATGCTGAAAAGGATTGTGCTGATGGCTC
AGATGAAAAAAATTGCACCTTCGCCTGTGAACCAAACCAGTTTGAGTGTGAAGACACCATATGTATCCCAGGCAGCTG

GGTTTGTGATGGCAGAGAGGACTGTCCTGATGGAAAAGATGAGAAACACAATTGCTCTCTTAACTGCACTGATGACCA
GTTCAAGTGTCCAGGTGGGCCATGTATCTCCAAGGATTGGGTTTGTGATACTGACCCTGACTGTCCTAACGGTGAAGA
TGAGAGAGTTGATGACATTGCTAAATGCAGTCCTGTTGTCTGCATACAAGATCAATTTACATGCTTGAATCGACGCTG
TATCAAAAATGAATTTTTCTGTGATGGAGATGATGATTGCGGAGACAACTCAGATGAACCAAACATGTGTATTCCACT
TAACAGTCAATGTTCAGAAAATGAGTTCCAATGCCAACACGACTTGAAGTGTATCCAGGGAATCAAACGTTGCAATGG
TCGCATCGACTGTCACGATCATTCAGATGAGAAGGACTGTTTGACCTCGGAGTTTGAGAACTTGAACCCATGCCACAA
CACCTCCCAGTTCCAGTGCTCCAACAAGATGTGTATTAATGAAAGCCTTGTTTGCAATGGCAACGATGACTGCGGGGA
TGACTCTGACGAGCCTTCGAACTGTGGTATTAATGAATGCCATTCAAGACGCCCAGTTTGCAGTCAGCACTGCGTGGA
CAAGAAGGTTGGATATGAATGTCAGTGCCACCCTGGATACAGCTTCATGGAGGACACTCATGATAATGTGTCGGAGAC
CAATAACAGAAATAAGATTCAGAAGACCTCACTCAACAGGAAGTGCATTGATGTGGATGAGTGTAAGAACATGTATCC
CTGCAGCCACTACTGCACCAACACCATTGGTTCTTTCAAGTGCAGCTGTGCTGATGGCTACAGCATGCTTGGAGACGG
AAGGTCCTGCGTTGTGGCTGATGGCATAGAGGTCCAACTTCTAGTATCCAACCGTTATTACCTGCGCCTGATTGGGAC
CACTAAAAACAACGTGACCACATTTACCAGTGAATTAAAGAACTCTGTTGCCGTGGACTACGACTGGAAGGATCAGAT
GGTTTATTGGTCAGACATCACCAATGACAGGAGCAGCATCTCAAGGATGGGCTTCAACTTCACCTCCAAGACCAAGAC
TTCCGACACAGAAAACCTGCACACCAACACTGTACGGAACCCTGATGGCCTTGCTGTGGACTGGGTGGGTAGAAACTT
GTATTGGTGTGACAAGACCACGGACACCATCGAGGTGTCAAGACTTGATGGCAAATACAGGAAGGTCTTACTGAGGCA
GCAGCTCCAGGAACCCAGGGCCCTTGAGGTTTTCCCCCAAAAAGGATTGCTTTTCTTTACTGACTGGGGAAACAATCC
ACATATAAGTCGTATGAACATGGATGGCACAAACCTCATGCAGATTGTCAACACAAGCATTGCCTGGCCCAATGCCCT
GACCATTGATTATGTCACTGAAAAGGTATTCTGGGGTGATGGAAGCCTCGATTACATAGGTATGGCTGACCTTGATGG
CTCAAACTTCAGGTACATCATAAAAGATAACAACAAAATCCCCCATGTGTTTGCTTTGGGTATCTTTGAAGGCCTTCT
TTACTGGACAGATTGGGAGAAGAGCAGTGTCTTGTCGGCTGCCAAGTTTTCTGGTAACAACATCACCAGGAAGAAGGT
CTTTTCCCAGCGACCTATGGACATTCAAGTAGTGCACCCATTACGTCAGACAGCTGTTTTGGATAAATACAACATGAG
TCCCTGTGACTACCAGCAGTGCACACATCTCTGTCTGCTTAGGCCAGGGGAGAATGGTGTTGGTGTGCAAGCTGTTTG
TGCCTGCCCAGAGAGCCATTACTTGGCCAATGATAGTCGATCATGTATCTCTAACTGTACAAGCTCCCAGATTTTGTG
TTCCTCCACCTCCAAGTGTATTCCATTCTGGTGGAAATGTGACAACAGGCCTGACTGTGAAGATGGCAGTGATGAACC
AAAGGATGAATGTGGCGCGTACCACTGCAGCCATCCTGGCATGTTCCAGTGCACCAATGCCACATCAGCCAGAGATTG
CCTGCTACCCACACAAATCTGTGATGGAACACAGCACTGTCATGATGGGTCAGATGAAAACATTTGTGCCAAGTATAC
ATGCATGGAGCACTATGCTAAATGTCATGCAGACAACACATGCATTCCCAAATTCAAAGTATGTGATGGACATCCTGA
CTGCAGTGATAAGGAGGATGAGAATAACTGTGAACAAGTAGACTGCAATCCTGAGCAGTTTAAATGTGCAAACAGTCG
TTGTGTGCCTTACGTCTGGAAGTGTGATAATGATGACGACTGTGGAGATGGCAGCGATGAACCAGAAGACTGCAAAAC
TAGTCCTTGTCCAAAAGATTTCTCTAAATGTAATGTCACAGGAAAGTGCATTCCTGAGAACTGGAAGTGTGATGGAGA
TTATGACTGTGGTGACCAAGACACATCAGATGAAGACTTTGATGAATGTCACAGTCAAACGTGCGACCCCACTTTCTT
CAAATGTCACAATGGCCACTGCATCCCTGGCCGCTGGAAGTGCGATTTCCATGATGACTGTCGTGATGGATCAGATGA
ACAGAACTGTAACAATAAGACCTGCTCATCGAATGAGTTCAGGTGTGGCCATGGGAAGTGCATCCCCAAACCACTGAC
TTGTAATGGAGTGTTTGACTGTTGGGACAATACTGATGAAATCAATTGTTCCCTGAGCTGTGACAGCACCACAGAGTT
CCAATGTAAAAATGTGCCCCACTGCATTGCCATCAAGTGGCTGTGTGATGGAGAAAGTGATTGTGCAGATAATAGTGA
TGAGAAGAATTGTGAGCGTAACTGCACAGAGAAGGAATTCAAATGTAAGAACACACAGTGCAAACCAAGGGACTGGCA
GTGTGATGGGGACGACGACTGTGGAGATAACTCTGATGAAGATGAGCAACTCTGTGCCCAACTTGCATGCCCACCTGG
CAGATTCAGGTGTAAGAGACACAAGTGTATCTGGAACAATATGGTGTGCAACAATGTCAAGGACTGCCCTGACGGGGA
GGATGAGGACATTACTCAGTGTGCCATAGCCAAGGAATGCACTCGACCCAGTTTTCTATGTGACTCTCTCACCAAGTG
TGTACATCAAAATCAGATGTGTGATGATGTGACTGACTGTGTGGATGGCTCGGATGAAGATAAGGCTTTTTGTGACTC
ATTGAAGAGTGTCAATTGTTCAGTGAGAAATGGTGGCTGTGAACACCACTGCAACACCACAAGCAATGGGGCCAAGTG
TTCATGTAAGAAGAACTATATCCTGAATGAAGATTCAAAGTCCTGTTCATTTGACAACCCATGCAACCATTACAACAC
GTGTTCCCAGCTGTGTCATTACAACAAAGACCAAGGACTGGTGGAGTGTTCATGTGCCAAGGGCTTCACTAGAGCTGA
ACATTCATCATGTTTTGCCACAGGCACAGAACCTGTCTTCCTCTTAGCTGAGAAAGGAACCGTGGTCTTGAGGTACAT
GGTGGACTTTGATCAAACTGAAACCATTATTCCCAAGATGGACAACAAAAACAAGCACATAGTGTCTATTGACATGGA
CATATCCCAGAACATTGTGTTCTTCATCAACCAGACAGGGACAGAATACTTCCTGATGAAATCAGAGCTACCAAAGAA
CCTCAACAAAACTAGAAGGAAGAGGCAGATCAGTAATAAACCACCCGAAATTCTTCATCTCCAAGATCGGCAACTGTT
TGAGATGGGCGGCCTTGCTGTGGACTGGGTGACCAAGCACGTCTACCTGACAGATGTCACCAACCGACAGATTCATGT
GTTCAACTATAATGCAGAGAGAGGCAAGGTGGTGGCCAGGCAGCACATGTCACAGCCTTATGCCATTGCTGTGGATCC
CATCAAAGGATATATATTTTGGACTGATCGTGGAGTCCAGGCCAAGATAGAAAGAGCAAACCTTGATGGTAGTGAGAG
AACAACCATCGTGTCTAGTGAAATCTCCTGGCCTAATGGTATTACTCTTGACACCATTGGAGGCTGGGTTTACTGGAC
TGATACCAAGAAGCACACAGTTGAAGTGGCTAAATATGACGGCAGTAGGCGGCAGAAAGTATTTGTCACATCTGATGA

TCCCCCATTTGCTGTTGATTTCTTTGAAGACTACCTCTACATCGTCACCTACAAAACCAGCCAAGTGATCAAAGTGCA
CAAGTTCAAGCAGTTCAATTCTACTCTATTCACCACACTCAGCCATGCTTCGGACCTTCGTATCATCCAGGACAATAA
ACAACAGAAAGTCAGCTCCAACTGTACAGGGAATCCTTGTGAAAAAACTGAAATTTGCTTCAACAAAGCCCAAGATGT
CAAAGGTTTCATCTGTTTGTGTCCCGATGGAGCCCAGAAGAATGGAAACAGTTGTGACTATGAGAAGCCCCCTGGCTG
TGAGAACTATTGTAAAAATGGCAGATGTGAACTGACCAAAGTCACTGGAAAGCCCAAGTGCATATGTGAGCAGTCATA
CTTTGGTGAACAATGTGAGACCCACTCATGTCAAGGGTACTGTCTCAATGGCAACTGTACCCCATCACAAACTCCTAA
TACCCCACCCATATGTAGATGTTTGCCAGGATTTTCAGGAGAGAGATGTGAAAAATATGCATGCACGAGTTATTGCCA
AAATGGAGGTGTTTGTCGTATAATTCAAGGACAGCCCCACTGCTTCTGTCCCCTCTTGTATGATGGGGTGAGATGTGA
GAACCAAAAGCCTGAAATGGACTGGTGCTCACACATCTGTAAGAATGGAGGCCAGTGCATCAAAGAAAGGTTTGGCTA
CATCAGATGTAATTGTACTTCTGGCTTCACAGGCCAGAGATGTGAGCAATGTGTTGGCTTGACCTGTGCAAACGGTGG
GACTTGTTTTAGGGATGCCACCACCAAGCAGGCCAAGTGTGAGTGTCTAGAGGGTTTTGACAGTAGGACCAACTGTGC
TAACATTTTAAATTGTTCCATGATGTGCACTTCAGGTGTCAAATTATTGCCAGAGCAGTGTGACTGCAACTGTAAACC
TACATACTTTGCTGCAAACTGCAGGCAGTGTCCAATAGCTTGCCCCAAGAACCAAGTTTGTTTTACAGGTAGTGGCAA
ACCCAATTGTGAATGTGATCTCATTCATGACGGGCCCAACTGTGAGCGATGCAAGTGTGAGACAACCGGTCTTTGCAG
TGTTGACACAGATGGAAAACCTGTTTGCAACTGTAGCATGGAGACATTTGGCCCTTTATGTCAGTACGGGTGTGTTGG
ATCATGTGGGAAGAACGGTCGCTGCTCGGAGTGTTATCTCAAAACCAGCAGCCACCACCCGCAGTGCATGCCAGGGAA
ATGCATATGCTATGAAGGCTTCTCTGGTCCTACTTGCGCGGCACACACAGAAGGCTCAAAAGAACAGAGTACTGCTGA
CACAAATCCCTTGATGATTGCTGTACCGATAGCTATTGCCTTGATAGTTATTGCTGTCATTGTCATTCTCATCTTTGT
CATGAAGAGGAGAAATCGCAGGTCATCACAATATGGACACAAACGTATGGATGAAAGGGGAGGTAACATGAACGTGAC
CAATCCTGTCTACATGAGGAGGGACGCAGAGGATGACGAGGAGGATGAGTGTGAGCCTTTGAGTGGAGGAGCAATATT
TACTGGTGACACTTCCTCTAATTTTGCCAGCCCAATGTACAATGGGTACTCAAATGACAGCACTCAGAAGTTACTGTC
TTCCAGTGTTACAGAGGAACACAACACCTTTAATGATGGAGATGTTGTGTTTGCTGGTAGTACAAGGGATCACACGGA
GGACATACAAACCACCATAGCATAGCATTAGTTGGACTGGCTGAATGGTGGAGAGTGAAAGCTGAGTGGATGAAGGCT
GTGAGCTGAATGCATTTTTTAACCTAATTCTTCCAATGTGGGCGAATGACAATGTGAAGTGAATCTGATTGGGTTGCC
ATTTGTCTAAGATTGCCTAGATCTGCAGCTAAGTGAATGACATTGTTCAGCTGTGGTCTCTGTGTGGCCAACCATCTC
AGTCTTAATGTTACCTGTCACAATGCTGGGGAAATCTTGGACCGAAATTATATTTTTGTGAAATGCTGTACATAGAAA
GATTTATGGAACTAACATTTTCTGTTATACCTTGCACTGAGTGAACTCTACTGTTGAAGGATAGGGACACACTGTTGG
TCACATTTCAAGGAAGTCTCAGCAAACTGTTGGTCACATTTCAAAGAAGTCTCAGTCCCAAGTTATTTAAAAATCTAC
AAAATTTCATATATATATGTATAGAGAGGCATATAAAGCAAACTTAACCAGAATGAAAATGTTTGACTGACCAACTCT
TAGAGGGTCTGATTTCACTTCAACCATTCAAAGTGATACATGTATCATACAGTTTGCAGCGATATTTTCATCTGTTAG
GTTCCAATCAATATGTTAATGTTATGTGTACTTAATCAAATACTGCATCCAGCAGGAAGAGAGCTGTATTTTTCTTGA
ATCCATGTTTGATTGTGATAACAGCCAATGGTGACTAATTCTCTTGCAGTATTACTAAACTTTCTTAATGATGGACTG
TGTTTCAGATAATCACTATAATAGAAAAAAAGTAAATTCATTGTTTGATTCTAACCAGAAACATATCACAGAAACTTT
GATAATATTCTATTTGAAATAACAACAATGTTAAACCCACACCCACTCTCCATTAAATAAATTAAAAATATTTACATA
ATTTTTTTCTGTAGAGCAGCGATTCTCAACCTATGGGTCGCGACCCCTTTGGGCGTCGAACAACCCTCACACAGGGGA
CGCCCAAGACCGTCGTAAAACACATATTTATGAAGTAGCAAAGAGAATAATTTTATGGTTGGGGGTCACCACAACATG
AGGAACTGTATTAAAGGGTCGCGGCATTAGGAAGGTTGAGAACCACTGCTGTAGAGCATCTTGGTCAAATCTGTTGAG
TGCACTGATTGAGGCAACTGTTTAGAAATGTTCAAAGTAAAAAGTGAAATGACTTTGAAAGTGAACCATTATTTAGCT
GAACCTCAGAGGTGAAATAAAGTAGTTATTGAACCATTAAATAGTCTACCTCTGTGTGTCTTAGACATACCGGGTCCA
TTACAGTAACATTTACAAAGCAAGCAACTTTCCCCCTATCAATACCAGCATCATTTCATCTGTTGGGTGAACCTGTTG
CCTTGTTTCATCATGGACAGCATGTATCCCCC

>Lymnaea stagnalis_ apolipoprotein E (apoE) receptor_protein

MAQLSCGSLRDVYPTWEVKTQYSLKSVKAIAKDWLSGNWYFADEFKEIIFFCTSDGSYCQTVLTSGIKRPKSLAVDAS
KGYLFYSDWSSNDLAHVGRIDLDGSNPLKLASIKIVHPNGLTADIANSHLYWGDSFLDVIERVDYIGHKRIVIAKGID
VFHVFGMSILQNYLYVVNHLNNTIVRIHRYNSTVPNKIMLKASRKPGTIKLFHPVAQPFEKFDVCSWAQCDQICVPVP
NPVNDSVKASCVCKLGFKQAPDGKCTKQDATKFMLLTNGQQGMIHFISTDSSEVKRDLYPPIANLGRPTCVDFDYLEG
YIYFFDVSSHTLRRRRFDSDKEPEAVVTQGINCDGLAVDWSGRNIYCSDSGRNKIVAISLRNFSHIYTVIDSDKLKEV
INPKALAIDAKNGKLYWTDWVISPGAGNASINWVYMDGSNWARIQHKEIQWPNGLVLDPNTQMLYWTDAYYDRIESMS
IDGTHRQVILNFTSNLHPFGITKHGNKLYWAESMDGSLMEIDLATKKVTNYRNSSAPVFDVKLYANNSQPNAGHPCST
NNGGCSDLCLLTPGGGAACKCADGRTSTNNGVLCTGKLTPGNIAPRKCQGLLEFECANGECIHSTRTCDGKFDCTDKS

DEDMMNHTCQDEGMFKCNTSTCLYMQFKCDGEVDCVFGEDEKNCSDHSCMADHFQCATSKQCVPLTWKCDGEKDCSDG
SDEDEAHCCKVTCKPEEFACLDGRCIRYEFRCDNEYDCMDNSDELDCQEWCDPVREFKCINESRCIPKIFQCDGEKNC
QDGADEKGCEKHERICHKDEFSCSDGTCLKLEYKCDGSNDCLDGSDEINCLKNMTDKVCHWTEFKCSDGTQCIASVWR
CDQEFDCKDKSDEKDCAKCQSPNFACKAHQTMCIPPEKLCDNNNDCPDYSDEGRLCEYDMCLNNDCEAKCHKSPDGFV
CSCPENQKLRPDNKTCVDINSCEKWGICSQLCQPTFHGHKCYCSPGYTLQADGYGCKPIDPDPVYIIFANRHEIRRLN
THDKSMTHLVSNLQNAIALDFHYNQSLVFWTDVSNDKIYRGEINANSVTKIEPIIEFGLATTEGVAVDWIANTIYWVE
SNLDQIEVAKLDGSERATLIAGNMTSPRAIVLDPRVGKLFWTDWDGAHPRIESCSMAGEPETRTVVYDIRNQKGAGWP
NGLAVDYETKRLYWVDARSDSIHCITYEGKDHQLILKSNRALSHPFSVTVFEHYIYWTDWRFNTLVVANKYNGSDVHV
VHSTYQQPFDLQVYHPKRQPQMANPCLNSPCSHLCLIGDGLKPVCRCPHRYKLSDDKRVCEKDNIFLLFTKENEIRGV
DLENAHYNVIPSITVPFVENATSIDYDVTEERLYWTDMKKNVITSAYLNGTGITTVIDSGLSNPSGFAIDWVSKNMYF
SSYNDVEGYISVAKLDGAYRKEIYRSTFASKPNSIAIHPSKGVMFWSDLGGEHHTIWKANMDGKKSGVFVEVVKKPAS
LTLDMVYNRLYWISQEEGAIFWCDVSLSKCNATLDQNVSMKEPISMTLQMFTVSEQSYFMLTDSTLTKCSLFFQFQNG
HKITMREDTKNVFDLRVYDPSSRQGTTNNCSVKNGGCEQLCLPTPDRGGIVCECTVGYQAVAGGKCHGIDTFILYTQA
SEIQGMLLDAQTHSPALASISKISRATSVDFHADNGHIYWVDSDLRLISRIKRDLSGREVIVSQGISGAESLAVDWIA
GNIYWTDQGHNTIEVIRLNGSQRHVVLHEDIDKPRSIAVHPAKGYLYFANGGVSPKIVRTRLDGSERVDFVSSTDAQP
VKAPFGLAIDFDTDDLYWCDKDLDFIERVTPSGQRFSVVTHNLTDCMSVAVHKDRLYWADLTDLQGSIKYVNKTGRDV
TARDITVMKKNITKLKDIKVFDGEAQIGSNPCGENNGGCEELCLYRGNSNFTCACSYGRLKEDGKSCAEHDSFLLYSE
ITSLRRSLILANSTDRNAPRHPIQNETYMKNVIGLAFDYATERIFFSDIQQGNIQAVFFNGTGFRIIKEGVGSAEGLAF
DPLQKHLYWTSYSGSNINRISFNGLSPTKNDIFYQLDHTDHPRDIVVNSCIRRIFWTNWSDRRPSIQTSSYDSDGSTD
SSESIITEGIRTPNGLTIDHKAQKLYWSDARLDKIERCDFDGSNRFIVVTSIPEHSFGLAVYGDFLYWTDWVVRAVVR
ANKYDGSRTTFLKKNINRQPMGIIAVANDTDDCMLNPCFENAFGCAEICVVSVKGDAMCQCGPGKKLLSDGKRCVSKD
LENCDGEDFICEDNKLCIPFNKTCNDIPDCLDASDESNHYCSTRHCPQHWYQCQNSSRCIMNSRICDGRNDCGDGSDE
LNCPCAKNEFRCNNGMCILAKYKCDFDSDCPDLSDEIGCSKTCEDLGIHGIQHIDLVSCNTTSMCIYPNWICDGSNDC
WDNNDEVNCDVLSGCTEFAFTCDDGKCILPQWKCDAEKDCADGSDEKNCTFACEPNQFECEDTICIPGSWVCDGREDC
PDGKDEKHNCSLNCTDDQFKCPGGPCISKDWVCDTDPDCPNGEDERVDDIAKCSPVVCIQDQFTCLNRRCIKNEFFCD
GDDDCGDNSDEPNMCIPLNSQCSENEFQCQHDLKCIQGIKRCNGRIDCHDHSDEKDCLTSEFENLNPCHNTSQFQCSN
KMCINESLVCNGNDDCGDDSDEPSNCGINECHSRRPVCSQHCVDKKVGYECQCHPGYSFMEDTHDNVSETNNRNKIQK
TSLNRKCIDVDECKNMYPCSHYCTNTIGSFKCSCADGYSMLGDGRSCVVADGIEVQLLVSNRYYLRLIGTTKNNVTTF
TSELKNSVAVDYDWKDQMVYWSDITNDRSSISRMGFNFTSKTKTSDTENLHTNTVRNPDGLAVDWVGRNLYWCDKTTD
TIEVSRLDGKYRKVLLRQQLQEPRALEVFPQKGLLFFTDWGNNPHISRMNMDGTNLMQIVNTSIAWPNALTIDYVTEK
VFWGDGSLDYIGMADLDGSNFRYIIKDNNKIPHVFALGIFEGLLYWTDWEKSSVLSAAKFSGNNITRKKVFSQRPMDI
QVVHPLRQTAVLDKYNMSPCDYQQCTHLCLLRPGENGVGVQAVCACPESHYLANDSRSCISNCTSSQILCSSTSKCIP
FWWKCDNRPDCEDGSDEPKDECGAYHCSHPGMFQCTNATSARDCLLPTQICDGTQHCHDGSDENICAKYTCMEHYAKC
HADNTCIPKFKVCDGHPDCSDKEDENNCEQVDCNPEQFKCANSRCVPYVWKCDNDDDCGDGSDEPEDCKTSPCPKDFS
KCNVTGKCIPENWKCDGDYDCGDQDTSDEDFDECHSQTCDPTFFKCHNGHCIPGRWKCDFHDDCRDGSDEQNCNNKTC
SSNEFRCGHGKCIPKPLTCNGVFDCWDNTDEINCSLSCDSTTEFQCKNVPHCIAIKWLCDGESDCADNSDEKNCERNC
TEKEFKCKNTQCKPRDWQCDGDDDCGDNSDEDEQLCAQLACPPGRFRCKRHKCIWNNMVCNNVKDCPDGEDEDITQCA
IAKECTRPSFLCDSLTKCVHQNQMCDDVTDCVDGSDEDKAFCDSLKSVNCSVRNGGCEHHCNTTSNGAKCSCKKNYIL
NEDSKSCSFDNPCNHYNTCSQLCHYNKDQGLVECSCAKGFTRAEHSSCFATGTEPVFLLAEKGTVVLRYMVDFDQTET
IIPKMDNKNKHIVSIDMDISQNIVFFINQTGTEYFLMKSELPKNLNKTRRKRQISNKPPEILHLQDRQLFEMGGLAVD
WVTKHVYLTDVTNRQIHVFNYNAERGKVVARQHMSQPYAIAVDPIKGYIFWTDRGVQAKIERANLDGSERTTIVSSEI
SWPNGITLDTIGGWVYWTDTKKHTVEVAKYDGSRRQKVFVTSDDPPFAVDFFEDYLYIVTYKTSQVIKVHKFKQFNST
LFTTLSHASDLRIIQDNKQQKVSSNCTGNPCEKTEICFNKAQDVKGFICLCPDGAQKNGNSCDYEKPPGCENYCKNGR
CELTKVTGKPKCICEQSYFGEQCETHSCQGYCLNGNCTPSQTPNTPPICRCLPGFSGERCEKYACTSYCQNGGVCRII
QGQPHCFCPLLYDGVRCENQKPEMDWCSHICKNGGQCIKERFGYIRCNCTSGFTGQRCEQCVGLTCANGGTCFRDATT
KQAKCECLEGFDSRTNCANILNCSMMCTSGVKLLPEQCDCNCKPTYFAANCRQCPIACPKNQVCFTGSGKPNCECDLI
HDGPNCERCKCETTGLCSVDTDGKPVCNCSMETFGPLCQYGCVGSCGKNGRCSECYLKTSSHHPQCMPGKCICYEGFS
GPTCAAHTEGSKEQSTADTNPLMIAVPIAIALIVIAVIVILIFVMKRRNRRSSQYGHKRMDERGGNMNVTNPVYMRRD
AEDDEEDECEPLSGGAIFTGDTSSNFASPMYNGYSNDSTQKLLSSSVTEEHNTFNDGDVVFAGSTRDHTEDIQTTIA

>Lymnaea stagnalis_choline acetyltransferase (ChAT)_mRNA

GTGTCACCGCACAAGAATTCGGACCGCTTTAGCTGTACATGTATATACACACGTATATATAACGATACATATACACAC
ACGAGAGACATACGAGCAGCGCATGTAAACGCTAACAGCTGTCACCGGAGCACACGCTTAAACCGAGACCCGTGATAA
GTTGCCGATCGAGATGGGGAAGACAACAGAGTGCCCTTTTTCGCCCAGAAAGTTCCCATTACCCAAGCTGCCAGTGCC
CGATCTCCAGGGTACCATGGAGAAATATCTCAGCCTGATCAAGACAGTGGTGTCCCCTCAAGAATATTCCAGGACCAA
ATACACCGTGGACGAGTTCTGTAAGCCCGGGGGTGTGGGGCACGAGCTCCAGGAGTATCTCTTGAAACGACAGCAGTG
CATGGACAACTGGGCCAACGAGTGGTGGCTTAATGACATGTACCTCAACGTCCGCATCCCGGTGCTCATCAACCCCAA
CCCAGCTGCCGTGTTTCCATACCAGGGATTCCCGTCTCACAGAGAGCAGATCAGATTTGCAGCAAAATTCATCCGAGG
CATGCTGGACTTCAAACACTTAACTGACACGCGAACGCTGCCCATAGAAAGATGCAAGTACAAAGAGAAAGGCCAGCC
TCTGTGCATGGACCAGCACTACAGACTGTTCACGTCGTACAGAGAACCAGGGAGGGAGAGAGACGTGCAGAGGACAGA
CTTGGGGTTCATGGGACGAGAATACATCGTCGTCGCCTGTAACAATCAGTTCTACAAGGTGGATGTCCAACGGGAGGG
GGATGAGCTGACAGAAGCGGACATCTGCTTGCAGCTATCACGTGTCTTAAACATGGCCGAGCAGGAAAGTAACCAATC
AGAACCAGTTGGTCTCCTGACGTCACAGAAACGAACCTTGTGGGCCGAGCAGAGAGAAAGACTCCAACTAGATGCCAC
AAACAGAGCGAATCTCCACATCCTAGAGAACTGCCTGTTCCTGTTGTGTCTGGACAAACCCACGGTCCCGTCACGGTG
CGGTATCCAAGGAGGAATGCTTAACGACATGACGGCACGAACCCACCACATCATCCACGGACAAGGCGTCCACAACAA
CTCGGCCAACAGATGGATGGACAAGACGATACAGGTCATCGTGTCAGAAGACGGAACGTGCGGTATCAACATGGAGCA
CTCGGTCGCCGAAGGGATTGCTCTGGGTCACATGATCGAACACGCTTTCGGTGTCATGGGCAAGGAGAAGATTCAAGA
CGGCCCCCGTGACCCCAACAGCCTACCTCACCCCAAGTGTTTGCAGTGGAGCCTGTCAGCACATTCCCTGGCAGACAT
TGAGCAGGCCAAGGAATCAGTTGACAACATGGTGAACGACTTTGACCTGACTGTGTTCAGATTTGAAGGCTACGGGAG
GGAATTCATCAAAACCCAAGGCATGAGTCCGGACGCCTACATTCAACTGGCCCTTCAGTTGACCTACTACAAAATCCA
CGGGACTTTAACCTCTACGTACGAGAGCGCGTCAGTGAGGCGATACAGGCAGGGGCGAGTTGACGTCATCAGGGCCAA
CTCTCCACCAGCTTTGACTTGGATCAAAGCTATGCTTGGACAGACAGAAGCCACGGAGGAGGACAAGCTTAGACTTTT
TACAGAAGCCGTCCACTGGCAACAAGATTACATGCTTGACACTATCCTTGGCTACGGAATCGACTTACATCTGTTGGG
TCTGAGAGAGGCTGCCAAGGAGATGGGGATCCCCACACCTGAGTTTTTCAATGACCCCTCCTACAAGGAGCTGAACAC
GTTTCGCCTCTCAACAAGTCAGGTCCCCACCGTGTCCGATTACTGGATGGGCTACGGGGCCGTTGTGCCTGACGGGTA
CGGCTGCTGTTACAACCCAAAGCCTGACAGCATTATCTTCAGCGTGGCCACCTTCAACACGTGCCAGGACACGTCGTC
CGAGATGTTCGCCCACTCCCTGGAGTCCAGCCTGCTTCAGATGGCCGAGCTGTGCACATACGATCCTGAGACGGCAAC
CATTAAACAAAGATAAGACTGGCTAAAGACGAAAAGGAGGACCACCCCCCCCCCTTTTTCCCTTGGGATGCAAGCCA
TCATCAAAATCTCACAGGACACATTGCCAATATTTTATAGTGTATATTTCTTGAAGGTGTGAGTTTTATTGTAAAGTT
GTAGATGTCCCAACAATGAGTTAAAGACATCTCGCAGCCATATCAGCTTGTCTTAGGGCAGTTAAGACGGGTTGTAGC
TGTTGTTTTCAGTGGATTGAGTGGGTAAGGGGCTAGAAGAAAAACCATCATCACTGAATATAAAAATCTCTTTGGTCT
GAATAGGATTTGTCATTTAATGGCGGAAATCTATTAATAGAACATCTAGTCATACATTCTGGCATACATTGTGGCCAA
TGGCATTAAGATATTAATGGAACAATCCTGGTATTCTGTTTGGCCAATCTCTTCGGTACTTCTACGTTAATGTAAGCC
AGCGTAGTGGGGCTTAACCTAGACTGCTAAATAAATAGGCAATTCTAAGTGAGAAACTATGTTTTACGAATACATTGC
CTAAAAATGGTAACATGATGTAATGCGATGTGAAGTTTTCCAGTCTTAGAAATGACAAACTCTATAGGAGAATTTTTA
ACACTTCAATGAAATTCTTATTACATTTTTTCACTGATGATTTTTGACATGTTTACATTTACAAGATGTGACCTATAA
TTATAAGGGCCCATATTGGGCAATGAAACATGGAGTCTCTGAGTATACATTCGTTTGCTGATGCACACACTTGCTACT
GCAACTACGCATTAGTGTAGTAGTCGTAGTCGAGAAACAGAACATTTTGAATATCCACATTGTCTCTTCCGATTTATC
CGATGCCTAAAAACACAAAGCATCTCAATTAGGTATGCAAGATAATGTCAACACCTTATGTATCGATCTGTGAAAGAG
GGTACCACTTTTTATATAGCCCATGTATCTACAAAATGTATGCATTGGTGAGGCAGAAAGCTGACATTGTTGAATACT
CTTCCTTTCTTATAGGATATAATTATGCTTCTGCAGATCACACAACAGGAGATCTTCAATTATGTTGTAACTATAATT
ACTATTGTTGTTATTATTATCTTTGCTACTAATCCAAACTTGATTTTTGGCGTGATTACAGTTTTTTCTATAAAAAAA
ATGAACACAATGTCGTTTATTATTTAGAAACTGTCAGCAAACCAATTGTATAACTGGTTGCCATTGTTTGGGACCACT
GTATACCTGGTGGCCATTGTTTAGAAACTGTTAGCAAACCAATTGTACGCCTGGTGGCGATTGTTTTACAGTGAGGTA
TTAGACTCTACGTGTAGATGTGATTTTTGTTTCAACTTATTACTATTTATTAGCAACATGTGATATGTATTAATACAC
CCATTTTTAGCCAACCGGAAAGGGTTTAACAACGGTTTACAAGGACATGTGCACTTCTTTAATGAATACCCGTCAGTT
TTTTAAAACAAAATAAATACGAATCAACTTAACAATCATATTGTATTAAAGGTATAATCTTTAGAAAGG

>Lymnaea stagnalis_choline acetyltransferase (ChAT)_protein

MGKTTECPFSPRKFPLPKLPVPDLQGTMEKYLSLIKTVVSPQEYSRTKYTVDEFCKPGGVGHELQEYLLKRQQCMDNW
ANEWWLNDMYLNVRIPVLINPNPAAVFPYQGFPSHREQIRFAAKFIRGMLDFKHLTDTRTLPIERCKYKEKGQPLCMD
QHYRLFTSYREPGRERDVQRTDLGFMGREYIVVACNNQFYKVDVQREGDELTEADICLQLSRVLNMAEQESNQSEPVG
LLTSQKRTLWAEQRERLQLDATNRANLHILENCLFLLCLDKPTVPSRCGIQGGMLNDMTARTHHIIHGQGVHNNSANR
WMDKTIQVIVSEDGTCGINMEHSVAEGIALGHMIEHAFGVMGKEKIQDGPRDPNSLPHPKCLQWSLSAHSLADIEQAK
ESVDNMVNDFDLTVFRFEGYGREFIKTQGMSPDAYIQLALQLTYYKIHGTLTSTYESASVRRYRQGRVDVIRANSPPA
LTWIKAMLGQTEATEEDKLRLFTEAVHWQQDYMLDTILGYGIDLHLLGLREAAKEMGIPTPEFFNDPSYKELNTFRLS
TSQVPTVSDYWMGYGAVVPDGYGCCYNPKPDSIIFSVATFNTCQDTSSEMFAHSLESSLLQMAELCTYDPETATIKQR

>Lymnaea stagnalis_ amyloid precursor protein (APP)_mRNA

TTGCAAAATATATTTTTAGCTTTTGGTTCCACTCACTACACGAGGTAATTTCCCCATTTCATTCATATATTATTATAA
TCACACAAAAAAACACATCACAATCCAGCCAATCGTCGCGGGCTTTAAGGAAGGGATGGTTGAACTCTATGAATGACG
TCACGAAGACATTTTGGTTGGAAATTTTAGTATACAAGCAGTGTCCGATGCAAGTGGTGCCTTTATTTTTCTGCGAAT
AATTTGGATTACTTAAACAAGATGAGGCATCTTTTTCAAGTCGGTGCCCTTTTCGCATTAATACAGGTTTTGTTTTCT
GCAAGCCTTGAGGATAAGTATGAACCCATGGTCGCTTTCATATGCGAGCGACCTGCCATGCACAGGGGTGTCAATGGT
TGGATTGCAGACAAATCAACAGATTGTTTAGACAGAATGGAAGACATCCTGGCTTATTGCAAAGCTATGTACCCTGAC
CACAACATCACCAACGTTGTTGAATCCTCATACCTTGTGACCATTTCTGACTGGCCAATGGGCAATGCTGAGCGCCAA
CACCCACATCGTGTCCGCCCATTCCGTTGCTTAGTTGGAGGCTTTCAATCAGATGCCCTTCTAGTCCCCCAGCACTGT
GAATTTGATCACCGCCACGATCAGACTCAGTGTGAGGGCTTCGCACACTGGAATGTCATAGCTGACGACGCTTGCGAC
AAGAAGGGCATGCACCTGGAGAGCTTCGGCATGCTGCTGAACTGCAACCTGGGGAAATTCAGCGGTGTGGAATATGTG
TGCTGTCCAGTTGAGACTGAGACCAAATACCATCAACCCCAGACAGATGACAAGCCAGACAGCTGGGTCAACACCCAT
GATGACATGGACAAGGAGGAGAAGACCAATGCCAGCAAGTCAAGCACTGATCCCGCTGCAGCCACCAGCAGTAGCAGC
AGCAGCAGTGAAGAGGTATTGATTTTTGTTTGTTCTTTGCTACAGGAGGCCGATGAAAACACTGTTGACTTGTATGAA
GCTTACTTACGAGGCCAGGAATTCCCACAAAAGTACAACAATGAGCACAAGAAATTTGTTGCGGCCAGAGACAGGATG
AAGAAGAACCAGCAGCACAAGGTTACCAAGCTTTTGCAAGAGTGGCAGGCTGCCAGGGATCATGTTAATGAGGTCAGA
AAGTCAGACCCCAAAACAGCTGACACAATGGCAAAGGAAATCACTGCAAGATTCCAGAACTTGTACGCAGCCTATGAG
CAGGAAGATGACTCTGAGAAGGAACAGCTGACATCTCTACACCAGCAACACGTCCAGGCAGCGTTGAATGAGAGGAAG
AGGGATGCCATGGACAAGTACATGAGGGCCCTGGAGAAGGGAGATGCCGATAAGATAATCAAGTATCTCCGTGCATAC
ATCAAAGCCGAGGAGAAGGATCGCATGCACACAGTCAACCATTTCGAACACGTCAAGTATTCCTCCCCATCTCAGGCC
AAAGCCCTGCAGCCCCACATCAAGGATCATCTTTCTCTGACTGAGAAGAGAATCGAGCAAGCCTTGCAGCTGCTCTCA
CGTTATCCAGATATTGAGGCCAAGAACAAGCCTGAAATCTATGAATTCCTGAAGAGGTTTGAGTCTATTGCCAACAGC
ATAAGGGATGTCGTCCTGCCTGAGATTGTGGAGGAAAGTGAGTCGGAGGAGAGTGAGGAAGTCACAGAAGTCCCACAA
AACATTGTGAACAACAACGATGAGATTAACCTCGATGACAACAACGACTTTGATGTCAGCAAGCAGGAGATTGACACT
CAAGGGGACGACGTGGTGGAGAACGAGCACGACTACGAGAAGAAAAATGCCTTCGTAGCCAACCGTATGGAAGACACT
CACCACATACAACAGGGCTTTGTTGAATCCGCAGCCACCAGCAGCCAAGTGGGCAGCACCATCGGTATAGCACTTGGC
AGCGTCAGCGTGTTTGTCATCATTGTTGTTGCCATTATCATGTTGAAACGTAACAAGACAAGGCAGTCTGTAACACAT
GGTTATGTTGAGGTTGATCCTTCAGCTTCACCTGAAGAAAGGCACCTTGCTAACATGCAGATGAATGGTTATGAAAAC
CCCACATACAAGTACTTTGAGGTACAGAACAACCCAAAGGCTTGAAAGGGAGATAACCTTACCATTAATCATCCATCC
ATATTGTCTGTTCCTGTTCTTTTACACATCGTAACACCAAGAGAGGAGATGACCCATTCTTAACATCATGCCTGCAGT
AAGTACCTGCAAAAAAGCACAACAGAAACATCCAAAACAGTCCAGTGATTATGCACAACAATTGGGAACTTGACTGGA
GGACTGGAGGTGAAAAATGAATTTCAAAAAGCCAAGCAAGAGTAATGTTTTTTTAAAATTCTTTTTATTTTGATTTTC
ATACCAGGTTGTGTTTACAATTATTTTATCTTATTTTTCCCTTTTTACAAAGATGATTAGGAGGACTGTTTTAGCTTT
GTGCAACAAATTATTTCAGAGACGAAAATGTGATTTATCCATCATAACAAAGAACGTTTAAAATTTTTTAAAAATTTA
TTGGTAAAACATTCGTTAACTACACCCTTGAATTTTTTTTTACATTCATAAATTTTGACATCATTTTCAAAACATGCT
CATTTAAATCATTTTCACCAATAATATTAAAAAATAAAAATGTCAGAATTAACCTGTCCATATGTAAATGTGTGTGCT
TTTAAATCCCACTTGATGTAACACATAGTGATATGTTTCTACAATGGAGTTAGTGAAGGCAACGTTTGCTTGATAGAA
TTTTGAGCTCTAGCAAAATGAAATAATTTTTAATGCTATTAAAGTCAAGCCAAAATACTTAACAATCCATCATGGTTC
CAAGCTAAACCAATTAATTGTTAAGTTTAAATAAATTATTCATGCCGAGGATCAAATTTAAACATCCCCGTCTTAATT

TTTAAACACCAGAGAAAAAAAATCAGAAGCTAAAGTTTTTTAACAAAAATTCACATTCTTTAATCAGGATGTTGGAATA
CCTTTTAAAGACCTTTGCTGCTAAGGAAATAAACAAATTTAAACTTCAGCCTATCAGTTATAGTTAAAATTACAAGGT
TATGAGTAGAATTTCTTCTGTCCCCCTTGTCTGAGCTATGTGCCTCCTTCTGTGAAACTATTTCATTGATAGACATGT
CACACGGCTTTGTTAAATTTTTTACCACCATCATCATCATCTTGTCATCATTTCCACCATCTTTTTAGACGTCTTGTC
ATAATTTCAGATCTAGTTCAGTGGTTCAATGTACGCTAATTATAATCCCTGTGTCAGTGTCTTATTTGTAGGTCACAT
TCAGTTGTAGTTCTTGGTCTTTCTTGCCTCCTGTGCAACAGCTGTGCATCATTTTGTACATCTTCAATATTTCCTCAT
GGAATGGCATTAGTAAGGTTTTTATATCAGGTCTCAGTGTTATTTTTAAAAGAATTAAATTTTTTTTTTTTTTATCTT
CCCTGTGGTCTCTGAAGGGCTGTTCCAGAGTTTCTTTAATTAATGTGGTTCAATAAAAGTTCATGGAAACTGCTTCAT
GTGTAGTTTTTTTTTGGGGTCTTTTTTAACTCTTAAAAAAAAAGAAAAGAAAATACAAATAAAAATTAATGCGATCAG
TTTTTGTGTCAATTTCTTGTTATTCCCATATATTTCTAACCGGGAAAAAAAAAAATTAGAAGTTTTTACCTGCTTCTAT
CAGTTTTCTGTAATAGCTGTGTTTGTTATAGTTATATAACTTTTTACAATGTGAAACGTATGCATATTGGGACATGAG
AAGCTTCTAAACTTACGTACTGCATGCTTCTATTAAATCTGTACTTGTACCTCTTTGATCACAGAACAATATGAGACC
ACCAATTTTCTTTAAAATTGTACATATTTATAAATACATCTAGAACATTTTATGTTGCAGTGTTTGTTTACACCATAT
TTTTAATCAACCTTTTTTTTCTGTCGCTTTAGATATTAAAAGTTTTTGTGGAAAAAAATATTTTGAATACAAATTTGAA
TCTGTAAGCTGCCGTGAAACAGAATAACATTTACGTAACAAAATCTGTTGTAGCTTCACTTGTTAGTCCGGCCTTTTT
TAAACAATTTTTTATTGTAAGCTATTGTTACATCTCACAAGTAGAAACTCTACGACAACCTTAGATGTTAGCACCCAT
TATTATATTTTCATTACAATCAAGAACAAATGGAGTTCCTGTACACCTCCCTTGTATTTTAAAGTGTTGGCCACTGCC
ATGGTTTCCTACGTGTGCGTGACTGGGCTTGTTATTCATGTTCGCCTATTATTTCTATACTCTGTTCTGTTAATTTTT
TTGTTTTTTGTTCACTCTCATGATTAGTGCTGGGCTTATGTAACCTATGAAGATATAGCCTGCAGAAGTTTTAACATT
CCAAAATTATATCCATGAGATGCAGATAGGAAATAGCTGGCAAACACAGATACTAAATACTGGGTAAGATCAGGAAAT
TCAATGTCAGTGTGAATAACGCCATGTCTCTGCTTAGGTTTAAAGCCTGCCCATCTATCTTTAAGCCTAGTGACCGGT
GATGGTCACTTTGTGGAGGAACACAACCCCCCTTTTATTTTTTATCTCAGGTGGGGTGGGGGAGGTGTTAATCCTATA
TAGGGCATTCTTAGATTTTTCGAGGAATCAAGGGAGAGAAGGCTGTGAGAATCTTTTTTTTAAAAATGGTTGTGTTGA
CAAAATATGCATTCCAATATTTTACTGCTGGTCTCTGTGATGCCAGTTCCATACATTAAACATGTATTAAACATTTAG
TAGCAGCGGTGAATTTATGACGTAAACATTTTCCTCGAATGTCTTTTTGCTTTGGTTTCTGCTGGTTTCACTTGAAAT
GAACAAAGAAAATTAAAGAATTGCTTTGCATGATTTTATTTAAAGCCAGCAGTTCCAGACCAAAAAAACGTGTTTAAG
AATGACCTATATAGAATCCTGGAGTGTTTTAATTTATTTGTTTTCTTGATGGGACATTTCACTTGGCAACCTTGGTGA
ACATATGGCACACTTCTATAACACCCCCTGCTCCCCTATTTTCCTCACCACCGGCAAGAATGATGTGTTCTCAAGGGT
TTTGTTTCGTAGCCTTTAGGTTCTTAACTGTCCCAACAATCTATACTTTTAAGCTACTAATACTTCATGTTATTAATA
CTTATTATTATTATTATTATTACTACTACATATTTATTATCATCAATTACCTAGATAAGGAAATGGGTAATGACATGT
TAACGTTCCTTGGACTTGTAGCTGCAGTTTTGTGTGTTAATGTATTGCTACAATTGAGTTAAAACTTGTTTTGTGTTC
AAGTAAGATTTGTGTTTTTAAGTTGGGCTAGTTCATGATGGAACAATAAGCTTTTAATTAAAATTGATGTCTTTTTGT
TTTACTGATGCAGAACATTTTTGTCATAAATGCTGGTTTTTAAATGTCTCCACTCTTTTTCTCCCCAACAGTAAATTT
TGTTAACTAAAAGATCCATCCCCTGAAATTTTTGTCACAGCTATGTAAATTGCTTTGTTTAGAAATTAAAAGAAACAC
CATTTCCAC

Lymnaea stagnalis_ amyloid precursor protein (APP)_protein

MRHLFQVGALFALIQVLFSASLEDKYEPMVAFICERPAMHRGVNGWIADKSTDCLDRMEDILAYCKAMYPDHNITNVV
ESSYLVTISDWPMGNAERQHPHRVRPFRCLVGGFQSDALLVPQHCEFDHRHDQTQCEGFAHWNVIADDACDKKGMHLE
SFGMLLNCNLGKFSGVEYVCCPVETETKYHQPQTDDKPDSWVNTHDDMDKEEKTNASKSSTDPAAATSSSSSSSEEVL
IFVCSLLQEADENTVDLYEAYLRGQEFPQKYNNEHKKFVAARDRMKKNQQHKVTKLLQEWQAARDHVNEVRKSDPKTA
DTMAKEITARFQNLYAAYEQEDDSEKEQLTSLHQQHVQAALNERKRDAMDKYMRALEKGDADKIIKYLRAYIKAEEKD
RMHTVNHFEHVKYSSPSQAKALQPHIKDHLSLTEKRIEQALQLLSRYPDIEAKNKPEIYEFLKRFESIANSIRDVVLP
EIVEESESEESEEVTEVPQNIVNNNDEINLDDNNDFDVSKQEIDTQGDDVVENEHDYEKKNAFVANRMEDTHHIQQGF
VESAATSSQVGSTIGIALGSVSVFVIIVVAIIMLKRNKTRQSVTHGYVEVDPSASPEERHLANMQMNGYENPTYKYFE
VQNNPKA

>Lymnaea stagnalis_presenilin 1 (PSEN1)_mRNA

```
CAAATTGACAACACGTTAAAGAATTCGTATTTTTTTTAAATTCCCTTGGTAGAAAGTCGGAGGTTGTTCTGTTTAAGT
AGTTTAATACTAACTACTAACTATACTAACTATACTTAGACCATACTACTATATTTAGAACTTCCTCTAGATCTCTGC
CTCTTAAATAATGATATTAGTAATTAGTTACTAAATTGTTACAGTGCAGTATTTGCTGTTTTAAGTTACACTACTAGT
AATAACTATGAGCCTACACTACACTACTAATCTGAATACTGACAAGTTTTGAATGGATTACTATTTCAATGTCAATTC
AATAGAGATATTATTATTGAGTGGAAAGTCTAAGTCTTTGTCTAAGATTTGGAGGAGTTCACTTATTTCATTCAAAGC
TTTTATAGAAGTTTTTTTTATATTAAAGAGAGCTTTTTAAATGAGTTCACACAGTAAAACTTCAGGTGCTGTCTTTGC
AACTGATGACTCACCAACTGAAAGAACAAGCCTAATGAGTAGCTTTGTTAATGAAGCTGCTAGAGATGGTGGTTCCAC
TTCTGTCTTTGGCCCGGTTCCATCATCCCCGAATACTAATAGGATAACAACAATAGAAGATGGGTCTGCAACACCAGC
TATACCAGAGACAAGTGTGATAATTCCAAATGCTAACAACACTGGACAGGTTTCTGGGGCACCCAGGGACTCTCACAG
AAGAAGACGAAGAAACCCTTCACAGCGATCAGCACATCAACAGCAAGAAGAGGAAGAAGATGAAGAAACGCTCCTATA
TGGGGCCAAGCATGTTATTATGCTCTTTGTCCCAGTAACCTTGTGCATGGCTGTTGTGGTTGCTACAATCTCCACTGT
AAATTATTATACAGACACTGGTACATACTTGATTTACACTCCATTCCATGATAAAACTGACAATACTGGAACCAAAAT
CTGGCAGTCATTGGCAAACTCCCTCATCATGTTGGGTGGCATTGTGGTCATGACAATTGTGCTACTACTTTTATACAA
ATACAAGTGTTACAAGATCATCCATGGATGGCTTGTACTGTCTTCAGTCATGCTGCTGTTTTTCTTCTCGTATATATA
CATGGAACAAATTCTTCGTTCGTATAACACACCTATTGACTATATCACTGTGGCTATTATCATGTGGAATTTCGGAGT
GGGAGGGATGTTTTGTATACACTGGAAGGGGCCTTTACTTTTGCAGCAAGCTTATCTCATTATGGTTAGCTCACTCGT
TGCTCTAATGTTCATAAAATACTTGCCAGATTGGACGGCATGGACTGTACTTGGTGTCATGGTAGTATGGGATCTTGT
TGCTGTCTTGTGTCCTAAAGGACCATTGCGCATGCTTGTTGAAACTGCTCAGACAAGGAATGAGCCTATTTTCCCTGC
GCTAATTTATTCCTCTACCATGGTTTGGCTAATAACCATGGCCGACGATGAAAGTTCATCAAAAAAAGGAAAAAGAGG
AACATCTCAGGGCACTGGGGTTTCTGGAACCTCTGGTGGTGCACGTGGAGCCTCTGAAATTCATGAAAGTGATGATGA
GGGGGGCTTCAGAGAGCATCTGTCTAATGGAACAAACAGGGCCAATGAGCTAACGGCTAGTTCAGATTCACAAACAGC
CCGTAGAGCTGTACAGGCATTAGGAGAAATGAGTCATGGAAATCCTAATCCCCACAGGCACATTGCAGGGAGCAGCTC
AGACCCAGTTCAAAATCCGACAGAAACATCTGCTGTTGTCCCTAAAAAGAAAAAGACACCAAAACCCAGGCAACGAGC
AGCTGCACCTCAAGAAAATAGTGCAGAAGAGGATGAAGAGAACAATATTGTAAGATTTCAGAAGTTCGAGAGAAAACA
TATCTGTAATTATTATTTATTCCCCAAATTACTAACTCATCATTTTATTTTATTTGCAGGAGGTGTTAAATTGGGCTT
GGGAGATTTCATCTTCTATGGTGTACTAGTTGGAAAAGCTTCATCCAAAGGTGACTGGAACACAACCTTGGCCTGCTT
TGTTGCAATTCTAATCGGTCTGTGTTGCACTTTGCTGTTATTGGCCATATTCAGAAAGGCTCTACCAGCTCTACCAAT
TTCTCTCACTTTTGGCCTGGTTTTTAATTTTGCTACGAGTGCGCTTGTACGTCCATTTATGGACAGTTTAGCCAGTGA
GCAAGTGTATATTTAACTTTTAATGGATCTGATATCTAAACCTTATGAAATTCCAAATTCAAATTGTAATTTCCAAAT
AAATCTCTTACTATGATTATTAGCACAAGAATTTTACTGTCTCATTTAGCAAATACAAGTTTGCTCTTGCTTCTGTCA
CAAAACATTAACGCTTGAAAAAAAAAACAACTTAAGATTATTATTTATTTTTCTTTGAAGATGATTTTGTTTTTTTTA
GGCTTTTTTCATTGTTATGCATGGAGTCCAATGCAATCACTTGAATAAACGTTTTCAATTCTTTTTAATATATGAAGA
TAATATGTCAGCAGTTCAAAATTTATTTTTTCAGAGGAACCACTTAAAAAATTAGTCATTCAAAATTAATTTTGGGGA
GAAACACCCCC
```

>Lymnaea stagnalis_presenilin 1 (PSEN1)_protein

```
MSSHSKTSGAVFATDDSPTERTSLMSSFVNEAARDGGSTSVFGPVPSSPNTNRITTIEDGSATPAIPETSVIIPNANN
TGQVSGAPRDSHRRRRRNPSQRSAHQQQEEEEDEETLLYGAKHVIMLFVPVTLCMAVVVATISTVNYYTDTGTYLIYT
PFHDKTDNTGTKIWQSLANSLIMLGGIVVMTIVLLLLYKYKCYKIIHGWLVLSSVMLLFFFSYIYMEQILRSYNTPID
YITVAIIMWNFGVGGMFCIHWKGPLLLQQAYLIMVSSLVALMFIKYLPDWTAWTVLGVMVVWDLVAVLCPKGPLRMLV
ETAQTRNEPIFPALIYSSTMVWLITMADDESSSKKGKRGTSQGTGVSGTSGGARGASEIHESDDEGGFREHLSNGTNR
ANELTASSDSQTARRAVQALGEMSHGNPNPHRHIAGSSSDPVQNPTETSAVVPKKKKTPKPRQRAAAPQENSAEEDEE
NNIVRFQKFERKHICNYYLFPKLLTHHFILFAGGVKLGLGDFIFYGVLVGKASSKGDWNTTLACFVAILIGLCCTLLL
LAIFRKALPALPISLTFGLVFNFATSALVRPFMDSLASEQVYI
```

>Lymnaea stagnalis_notch receptor 3_mRNA

GGCAGGCTACAGGGGCGTCACCTGCATGGACGACGTGGACGAGTGTGCCCAGGATCCCCACATTTGCAGAAATGGCGG
GAAATGTATCAACAAGTTTGGAAGCTATGAGTGTGTCTGTACCCCTGAGTACACGGGGACCCACTGTGACGACAACTA
CATTCCATGTCTGCCCTCGCCGTGTCAGAACAACGGGACGTGCAACAGGACGGGACCTTACAGCTACGAGTGCTCGTG
TCTTTCAGGATTCAGGGGCCGTAACTGTGAGGTGAACATCGATGACTGCATCCACAACAGGTGCAACAACGGGTCTAC
CTGTGTGGACAAGGTCAACTCCTACGAATGTATCTGTCCACCATCCTACAGAGGGCGGTACTGTGACGATGACATCGA
CGAGTGCGCCAACAACCCGACCATGTGCCAGAACGGGGGCACGTGCATGAACAGCCCCGGCTCCTACATGTGCGTCTG
CGTCAACGGGTGGGCCGGGGTCCACTGCGCCCACAACGAGGATGACTGCCAGAACAACCCGTGCTACAGCGGCGGCAC
CTGCCACGACCGGGTCGGATACTTTGAGTGTGAATGTCCGATCGGTAAAACAGGCTTGCGATGTCACCTGGAGGACGC
TTGCCAGAGCAACCCCTGCAACGCCGGGGCCAGCTGCGAGACGTCGCCCATCGATGGGGAGCCCATCTGTGAGTGCAG
GAAGGGGTGGCGTGGCCGAGACTGCAGTCAGGACGTCAACGAGTGTGTGGAGACTGAAGACAGCCCTTGTGAACACGG
TGGCACCTGCATCAACACCAATGGTTCCTTCCACTGTCAGTGTCCCGTGGGCTTCTCTGGCAGCTTCTGTGAGAGGAA
CATCAATGAGTGCAACAGCAACCCTTGCATGAACGAGGGCACATGTCTGGACGAGACCGGGAAGTACAGCTGTCTCTG
CATGCCAGGCTACACCGGCACCAACTGCGAGACTGAGGTGGACGAATGCATCACGGCCAGTGGGAGGAGTCCTTGCTT
CAATGGCGGAGTGTGTCAGGACAACATCAACAGGTTCAGCTGTACCTGTCCCGCAGGTTTTGAAGGCCCAACCTGTCA
GCTTGACATAAACGAGTGTGACAGCGCACCGTGCAAGAATGGGGCCAACTGCATTGACAGCATCAACAAATACACATG
CGACTGCAAGAAGGGCTATAGCGGGCTGAACTGCGAGACGAACAAAAATGACTGCGAGGGAATCACCTGTAAGAATGG
CGGCAGGTGCGTTGACGGCCTGGACGCGTACGAGTGTCAGTGCTCACCTGGCTACACCGGACGGTTCTGCGAGACGCA
GGTCAATGAGTGCTCCTCAAACCCTTGCAGGTATGGGGGCACCTGCCAGGACCTGGAGAACAGCTACGAATGCAAGTG
TCCAACCGGAACATCAGGTGTCAACTGTGAAAATAACTTTGACGACTGCGCCATAAACCCCTGCAGAAACGGCGCCAC
CTGCAATGACGGCATCAACCAGTACTCGTGTACCTGTAAGGCCGGATTCACAGGTTCAAACTGTGATGTGGACATTGA
TGACTGTAAGTCCAATCCTTGCCACAATGGCGGCCGCTGCACTGATTTGGAGAACAGTTACCGCTGCGACTGTCCGGC
AGGGTATTACGACAGCCAGTGTCTCTCCAAGACTGATGAATGTGCCTCGAACCCTTGCACCAATGGTGGCCGATGCAT
TGATGGATACAACAGATTCGACTGTGAATGTCCACAAGGCTACACGGGCAACAGGTGTCAGAGTCAGATCAACGAGTG
TCAGTCCAACCCGTGTCAACATGGAGGCACCTGTCAGGATTTCCTAAACTATTACACCTGTTCATGTAAACGTGGATA
CACAGGTAAGAACTGCGAGACCAACAAAAACGAGTGCTCCGTCAACCCTTGCCTGCACGGCACCTGCATCGACCTGGT
TGATGACTATCAGTGTTACTGCGAGGGTTCCTTCACTGGCAAGAACTGTGACGTCAAGATGGATCCCTGCAGTCCCAA
CCCGTGCAAAAATCTGGCACTGTGCATGCCGCAGAACAACTTCCCCATGCTGTACAAATGTACCTGCCCTGTTGGATA
CACAGGGGCTCTCTGCGAACAAGACATCAATGAATGCGCGGGGACCAACCCTTGCAGAAATGGTGGCACCTGCCGTAA
CATCCCCGGCTCCTACAGCTGTACCTGCATCAATGGTTACAGTGGACGGCAATGCGAGGTCAACAACGATGACTGTGA
GCCGAATCCGTGCTTCAACGGAGGAACTTGTGTGGACGGCGTCAACAGCTACACGTGCCACTGTGTCGGGGGCTTCGG
GGGACAGCACTGTCAGAATGACATCGACGAATGCGCCTCCAGCCCCTGCATCAACAACGGCACCTGCACGGATTACGT
AGACTCGTACACCTGTTCCTGCAAGCAGGGCTTCAGCGGCGTCCACTGCCAGGTCAATGACAATGACTGTTCCAGAAG
TTCTTGCCTGAATAACGGCAGATGCATTGACCAGGTGAACGGCTTCACCTGTGAGTGCGCCAAAGGCTACACCGGCAG
CAACTGTCAGTACAACATCAACCCGTGCGACTCCAACCCCTGCCTGAACGGAGGCTCCTGTTACAACAAGCCGGGCAA
CAGTTACATGTGTTACTGCCCCTACGGTCTGACCGGGTCAAGGTGTGAGAGCTTTGTCGACTGGTGTAGTCTGAGCCC
GAATCCATGTCAGAACAAGGCGACTTGCGAGCAGATCGCCAATGACTTCCGGTGCACCTGTGCCAGCTCCTGGAGCGG
CAAGCTCTGTGATGTCCAGATGGTGTCCTGCAGCCTGGCGGCACAGAACAAAGGTGTGGATCTAAACTCATTGTGCCA
GAACGGCGGCACCTGCAGCAATGTTGACAGCGGCAGGTCTCACATATGCTCATGTCGCAAAGGTTTCGAAGGCTCCTA
CTGTGAGATAGACGTCAACGAATGTCTGTCGGCCCCCTGCCAAAATGGGGCGACCTGTAACGATCTCATCGGAAAATA
TTCCTGTCAGTGTGCTAGAGGATTCCAGGGACAGAACTGTGAGCTGAATGTCGATGACTGTGCCAACCAGCCGTGTGC
CAATGGCGGAGTGTGTCACGACCTTGTTGACAACTTCCAGTGCTCGTGTCCCCCGGGGACGGCCGGCTTCCTGTGTGA
GGTCAATGACAACGATTGTCTTCTCACTACCTGTCACAACGGAGGAGTCTGTGTGGATAAGGTCAGCGGCTATGAGTG
CATCTGTCCGCCTGGATTTGTGGGCAAGAGGTGCGAAGGTGATGTCAACGAGTGCCTCTCCAACCCATGCGACCCATA
CGGGACCCAAGACTGTGTTCAGCTGAACAACAGCTACAGGTGCGACTGCAAGGCGGGATGGACAGGTCGAACATGTAA
CATGAGAGTCCATTGTAATGAGAACCCCTGTGAACACGAAGGTCAATGCAGAGATACTGACTATGGGGCTGTCTGCAC
TTGCCTTCCTGAATTTACCGGAGAGTTCTGCCACGTTGCTACGTCTGACTGCGACTCCAGCCCTTGTAAGAACCAGGG
CACCTGCTCTGTTACGGCCAGCGGTTACACCTGCTCATGTCCCAAGGGATCCTCTGGGGTCAACTGTGAGATTGACAG
CTATGACAACTGTCAGGATAAGCCATGTCAGAATGGGGGCACCTGCAATGATAAAATTGACTCGTTTGAGTGTTGGTG
TCCCAGGTCATGGAATGGTCTCACCTGTCAAACATTGGACAAAAACTTCCAAGGTGGCCTAGGGACCCCGGTGTCCAC
AACATCTGTCAATGCCGTTCCCAAGATTTGCCTGGACAACAACTGTCCTGATAAAGCTTACAATGGTGTTTGTGATAT
TGAGTGTAACGTCATCGACTGTGCCTACGACAACACTGATTGTTCTTACGGGACGAAACCATGGAGAACTGCACGCA

GAAAACATCAGGTGGCAAGGCTTGTTGGCAGGTGTTCAAGAATGGCAACTGCGACAGTGAATGCAACAACCAGAAATG
TTTGTATGATGGTTTCGACTGTGAAAGACCGCTGCAAGACTGTTTGTATGAGAAGTACTGCCAGATAAACTACAACAA
CGGCCACTGTGATAACGGGTGCAACAATGCAGAGTGCGGCTGGGATGGTCTGGACTGTGACCAGGGAATCGAACAGGT
GATCCCTGGGACACTGTTTATCATTGTCCTCATTGAACCTGAGGAGTTCACTGCCATCAAGACAGACTTTGTCAGGAA
ACTGGGTCACCTGGTGCGGGCCATGGTGCGCATCAAGAAGACCAGCTCAGGGAAGGAGATGATCTACCCCTGGAAGGA
TAATGAGGATCAAAGTTCAGGACTGACACGCACCAGGCGATGGGTGGCTGAGTTGTTCAGCAGCAGTGAGGTGTCGCA
TCTCAGGTCAAAGAGATCCGTCCTTACCGGCACTAAAGTTTTTCTTGAAATTGACAACCGAGCCTGTCATAAGTTTGA
CAACCACACCTGCTTCGACAACACTGAAATCATCGCACAGTTTGTGGCTGAAAACATCAGAAACAAAGGCTTTGACCC
GGCCGTGGCTGTCCATCAGATCGGAACTGAAAAGGATGAACAGGTCCCAACTCCGTCTAATCTCAACGTCCTCTACAT
CATTGTCGGGGTGGCCCTTGGGGTCGTCATGCTGGTGTTTGGTGTCATCGTCATGCTCAAGAGAAAGCGCGTCTATGG
ACTCACCTGGTACCCGGAGAACTCCAAGAAGAGCTCAGCAGAGGGCGCCAAATCCGGACTGAACAGGAAAGGACCCGA
CGGCGAGGAGATGCAGGACAAGATGGATAAAATGGACGCCAACGACAACCACACCTCCCCACCACCAAGTATCGAGCA
CTGGCAAGAAGACGACGACGACGACAGGCCGATCAAACGTAGCAGAGTAAGTTTTTATAACAAAACCAATATTCGTAG
TGATGACCGACCTGGGATTGACAACACTGACATTCGCCAGTGGACCACCAAACATTTCGATGCTGCCAATGTTCCTCA
TCATCCCCACCTGGCCTTGACCCCACCCCAAGGAGAAGACAACCTGCAGGCCGATGTCAACGTAAGAGGCCCAGACGG
CTACACACCTCTGATGCTGGCTTCCATCCGTGGCGGGGGACTCGGTGACGATGATGACAGCAACTCTGGATCTGGCTC
CACTGAAGGAGGTGACAGCGACAACAACACATCCGTGGATGTTATTTCCAGTTTACTGATGCAAGGTGCTGCCATCAA
TGCTCAGACGGACAGGACAGGCGAGACCTCGCTCCACCTGGCCGCCCGCTATGCCAGAGCCGATGCCGCCAAAGTATT
GCTGGATGCAGGGGCCGACCCCAACGCCGAAGACAGCACGGGCAGAACTCCACTACACACTGCAGTGGCGGCCGACGC
CCAGGGGGTTTTCCAGATTCTGCTGCGGAACAGATCCACAAACTTGAATGCCCGCATGCACTGTGGGACCACGCCCCT
CATACTGGCCTGCAGACTGGCCATCGAGGAGACGGTGGAGGAGCTGATATCGGCCGATGCCGACATTGAGGCCACAGA
CAACAATGGTCGTACTGCCCTCCACTGGGCTGCCGCAGTCAACAACGTCGATGCCGTGATGATTCTGTTAAAAAACAA
TGCCAACAGAGACGTCCAGGATCAGAAGGAGGAGACTCCTCTGTTCCTTGCTTCAAGAGAGGGAGGACATGAGACGGT
CAAGGTCTTGCTTGACCACTATGCAAACCGAGACATGACCGATCACATGGATCGTCTGCCTCGAGACATTGCCCTCGA
GAGGCAACACTTAGACATTGTGGAACTTCTGGACACCTACAAGGTTGCCTCCGCTTCGACCGTTCATCTGGGTGGCGG
AGTTACTTCCCCCAACCACCACTCTAATATGGGTTACATGCACCAGACCAAACAGTCTAAAGCTAAAAGTCGGAAGAA
GAGCACCACTGTGAAAGACCCTGGTGCAATTTCACCACCAGGTGGCGCTGTGCCCGCCACCGTGACCAATGGGAAGGT
TTCTAAACCTAGAAAAAAGAAGGGAGATGGAGAAACTGCTGCGGGCAACAAGAAGAACAAACAGCAGAGTTTACAGTT
TTCATCCGCATCGTCATCATCATCCCAAATCCAAGGTCGCCATCCGTCAAGTAGTGACTCGTCACCGATGAGCACCAT
CTCACCTGCCAATTACTCCATCGAGTCTCACCACTCACCCCCTGGGTACGAGTCATCACCATTCGACTCAAACCTTGT
CATGTCAGCAGATGTCGTACGCCATATGGATGACATCCAGGTCATGGCCTACACGTACAGTTCTCCTGCACTTCTTGA
CCACAACAACGTGGACTGGACACGGGCCCAGTACAAGCAGTTGCACCAGCAGCAACAACAGTTGCAACAGCAGCATCT
GCAACAAAACATGGCAGGAGCTCAGATGGCCACCCTGCAGAATGGCCACATGGGGGCATCGGGTGGTGGAAGTGCACC
CAATCTTCAGAATGGTGTCATAGGAGGCACAAATTCTGGTCTAATGGGGACAGTAGGCCATGTACCACCCCCCTCTAT
TAAATCAAAAAATCTGCCCATGTCACCCACACACATGCAGGCCATCCATCAACACGCCCAGCAGCGTGCGACACATGG
TAGCCCCCACCATCGCAATGGGGACTTCCCTACGTCATTCCACCCGGACGGCACCATTCAGATCCCTGCACAACAAAC
TGTAGCACAAAATTACTCAACTTTGCAGCACAAGATGGTACAGCAGCATCACCACCACCAGCAGCAGCCGCAACAAAC
TATTCAGCAGCAGCAGTTGCCCCATCAACAGCAGCAAGGGGGCTACGCTATAACATTAGAACAGTACCCCACCCCCCC
CTCCAACAACAGCCAACACATTATGGACTCGCCTCCCCACCAGCACACAGCCTTCAGCTCATTCCGGCCCGACCACCC
CGACCACCTTCTCACACCCTCTCCAGACTCACCAGGTCACTGGTCCAGCAGCTCGCCGCAATCTGCCCACTCTGACTG
GTCAGAGGGCATCTCCAGTCCTGTCCCCGCCATAACGCACGGACCAAACACACATAGAAACAAGCGCGTCACGGAGCA
CGCCTATTTTTAGCCTCATCAGCAAGATTTGAATCGCTAACAGCTCCCTCTGGCAAACAGTAACTCCAGACATTAATC
TTTGGTGTATGTTTTTATAATGAATAACAAAACAAGAAGGAAAAAAACAAACAATCTGACCTTCACCTTAGATAAAAG
GGATTTCATGGTCCATGTATTCAGTGACTTGTTTAAAACTGTCAAGTAATCATGTAAGTTGTCATTTAATGAATGAAT
ATTTAATGCCAAAATGAATAATGGAAAATAAATTGCAATGAGCAATCTTCTCATTTTATAAAAGCTTTTTCATAGGAA
TTCAAAGACTTTCTGAGTAGCCTACCTCTTAAAGTATGAATCGCAGCCGTCTTCAATGAAATACTGTAGATGAACGGT
TAATTTCAATTACAAGTTACAATAATCTTTTTCAGTCATTAAATCTTTAGACTGGAAAGTTCAATATTTAATCAGGAC
ATTTACAAAGTACATGGGTTGGAATCATATTAATTTTGAAAAGGGACTTGGAAATTTAAGATTCATCTTAAAAGTAGC
TTCAAGTTAGCCTAAAAATTAAAAACATTAAGAGATGTCATCAAAAATTCTATTCAACTTTGATGGTTTTTAATATTT
TCTCATTGCAACTTACATAACAGTTATAACAGATTCTTCACATACAGTAACCCTTAATGCAGGTTCAGTACATATTTT
GTATGTTGTAAATATATATATTTTTGAAACGGACCAATATATATGTGCATGTCAGTTTTTAGCTAATGCAACTTTTTA
ACCAATTCAGTTCTTTCTTTCAAAAGAGCTCCTTTGAGCTGAAATAATGTAATTGACCAGGTTAATCATTAATTGATA
AAATGATAATTATCAAGCTGCCAGGTATAAAATGATGC

>Lymnaea stagnalis_notch receptor 3_protein

MDDVDECAQDPHICRNGGKCINKFGSYECVCTPEYTGTHCDDNYIPCLPSPCQNNGTCNRTGPYSYECSCLSGFRGRN
CEVNIDDCIHNRCNNGSTCVDKVNSYECICPPSYRGRYCDDDIDECANNPTMCQNGGTCMNSPGSYMCVCVNGWAGVH
CAHNEDDCQNNPCYSGGTCHDRVGYFECECPIGKTGLRCHLEDACQSNPCNAGASCETSPIDGEPICECRKGWRGRDC
SQDVNECVETEDSPCEHGGTCINTNGSFHCQCPVGFSGSFCERNINECNSNPCMNEGTCLDETGKYSCLCMPGYTGTN
CETEVDECITASGRSPCFNGGVCQDNINRFSCTCPAGFEGPTCQLDINECDSAPCKNGANCIDSINKYTCDCKKGYSG
LNCETNKNDCEGITCKNGGRCVDGLDAYECQCSPGYTGRFCETQVNECSSNPCRYGGTCQDLENSYECKCPTGTSGVN
CENNFDDCAINPCRNGATCNDGINQYSCTCKAGFTGSNCDVDIDDCKSNPCHNGGRCTDLENSYRCDCPAGYYDSQCL
SKTDECASNPCTNGGRCIDGYNRFDCECPQGYTGNRCQSQINECQSNPCQHGGTCQDFLNYYTCSCKRGYTGKNCETN
KNECSVNPCLHGTCIDLVDDYQCYCEGSFTGKNCDVKMDPCSPNPCKNLALCMPQNNFPMLYKCTCPVGYTGALCEQD
INECAGTNPCRNGGTCRNIPGSYSCTCINGYSGRQCEVNNDDCEPNPCFNGGTCVDGVNSYTCHCVGGFGGQHCQNDI
DECASSPCINNGTCTDYVDSYTCSCKQGFSGVHCQVNDNDCSRSSCLNNGRCIDQVNGFTCECAKGYTGSNCQYNINP
CDSNPCLNGGSCYNKPGNSYMCYCPYGLTGSRCESFVDWCSLSPNPCQNKATCEQIANDFRCTCASSWSGKLCDVQMV
SCSLAAQNKGVDLNSLCQNGGTCSNVDSGRSHICSCRKGFEGSYCEIDVNECLSAPCQNGATCNDLIGKYSCQCARGF
QGQNCELNVDDCANQPCANGGVCHDLVDNFQCSCPPGTAGFLCEVNDNDCLLTTCHNGGVCVDKVSGYECICPPGFVG
KRCEGDVNECLSNPCDPYGTQDCVQLNNSYRCDCKAGWTGRTCNMRVHCNENPCEHEGQCRDTDYGAVCTCLPEFTGE
FCHVATSDCDSSPCKNQGTCSVTASGYTCSCPKGSSGVNCEIDSYDNCQDKPCQNGGTCNDKIDSFECWCPRSWNGLT
CQTLDKNFQGGLGTPVSTTSVNAVPKICLDNNCPDKAYNGVCDIECNVIDCAYDNTDCSYGTKPWENCTQKTSGGKAC
WQVFKNGNCDSECNNQKCLYDGFDCERPLQDCLYEKYCQINYNNGHCDNGCNNAECGWDGLDCDQGIEQVIPGTLFII
VLIEPEEFTAIKTDFVRKLGHLVRAMVRIKKTSSGKEMIYPWKDNEDQSSGLTRTRRWVAELFSSSEVSHLRSKRSVL
TGTKVFLEIDNRACHKFDNHTCFDNTEIIAQFVAENIRNKGFDPAVAVHQIGTEKDEQVPTPSNLNVLYIIVGVALGV
VMLVFGVIVMLKRKRVYGLTWYPENSKKSSAEGAKSGLNRKGPDGEEMQDKMDKMDANDNHTSPPPSIEHWQEDDDDD
RPIKRSRVSFYNKTNIRSDDRPGIDNTDIRQWTTKHFDAANVPHHPHLALTPPQGEDNLQADVNVRGPDGYTPLMLAS
IRGGGLGDDDDSNSGSGSTEGGDSDNNTSVDVISSLLMQGAAINAQTDRTGETSLHLAARYARADAAKVLLDAGADPN
AEDSTGRTPLHTAVAADAQGVFQILLRNRSTNLNARMHCGTTPLILACRLAIEETVEELISADADIEATDNNGRTALH
WAAAVNNVDAVMILLKNNANRDVQDQKEETPLFLASREGGHETVKVLLDHYANRDMTDHMDRLPRDIALERQHLDIVE
LLDTYKVASASTVHLGGGVTSPNHHSNMGYMHQTKQSKAKSRKKSTTVKDPGAISPPGGAVPATVTNGKVSKPRKKKG
DGETAAGNKKNKQQSLQFSSASSSSSQIQGRHPSSSDSSPMSTISPANYSIESHHSPPGYESSPFDSNLVMSADVVRH
MDDIQVMAYTYSSPALLDHNNVDWTRAQYKQLHQQQQQLQQQHLQQNMAGAQMATLQNGHMGASGGGSAPNLQNGVIG
GTNSGLMGTVGHVPPPSIKSKNLPMSPTHMQAIHQHAQQRATHGSPHHRNGDFPTSFHPDGTIQIPAQQTVAQNYSTL
QHKMVQQHHHHQQQPQQTIQQQQLPHQQQQGGYAITLEQYPTPPSNNSQHIMDSPPHQHTAFSSFRPDHPDHLLTPSP
DSPGHWSSSSPQSAHSDWSEGISSPVPAITHGPNTHRNKRVTEHAYF

>Lymnaea stagnalis_potassium voltage-gated channel subfamily KQT member 2 isoform-e (KCNQ2)_mRNA

ATGGCGTCTCGTCTCCACAGCAAGACATCGGTCCAGGGCCGGATATACAATTTTTTGGAGAGGCCCACAGGGTGGAAG
TGCTTTATATACCATTTTACAGTGTTCATGATGGTGTTGATTTGCCTCATCTTCAGTGTCCTGTCTACCATTGACCAG
TACACTGAGTTCGCCATGGAGACTTTGTTTTGGATGGAAATATTTTTGGTGTGCTTCTTTGGACTGGAGTACGTCATA
CGTTTGTGGTCAGCAGGGTGTCGCAGTAAATACATGGGCATTCGTGGGCGGATACGTTTTGCAAGGAAACCCATCTCC
ATCATTGATTTAATCGTGGTCGTCGCCTCCATATGTGTTTTCACCATCGGCTCTGAGGGTCAAGTGTTCGCCACATCG
GCCATCAGAGGCGTTCGCTTCTTGCAAATATTACGTATGTTGCACGTGGACAGACAAGGGGGCACCTGGCGTCTGTTA
GGCTCTGTCATTTACCTTCATCGGCAGGAATTGATAACGACACTGTATATAGGATTCTTGGGGTTAATTTTCTCGTCT
TACTTCGTGTACCTGGCTGAGCGTGAGGACGGCAGGAAGGACTTCTCTAGCTATGCAGACGCCCTTTGGTGGGGAGTG
ATAACTGTGATGACTATCGGCTATGGTGACAAAGTCCCTCAAACGTGGATGGGGAAAATAGTGGCTTCCTGCTTTGCT
GTTTTTGCTATTTCATTTTTTGCCTTACCAGCGGGTATTTTGGGCTCTGGTTTTGCTCTGAAAGTTCAACAGAAGCAG
AGACAAAAACATTTCAACAGACAGATCCCAGCTGCTGCAACACTCATACAGTGCTTGTGGAGATGTCATGCTGCTGAA
CCACATTCCAATTCTGAAGCCACATGGAGAATCCACATTCATGATCCTTCCAGTGAGGAGACAGTTTTCGCACGTATG

GCTCGGCGGGCGAGCTTGTCACTTCGCAAGCGACGAATGTCTCGACTGGATTCTACAAGTACTGTTCCAAACCACCTT
TATAGAGAGTCTATGTCCTCTTCTATGTCGTATGCCGATGATAGACTTGGTAGGACTCAAACCAGCTGTCTTTTTTAA

>Lymnaea stagnalis_potassium voltage-gated channel subfamily KQT member 2 isoform-e (KCNQ2)_protein

MASRLHSKTSVQGRIYNFLERPTGWKCFIYHFTVFMMVLICLIFSVLSTIDQYTEFAMETLFWMEIFLVCFFGLEYVI
RLWSAGCRSKYMGIRGRIRFARKPISIIDLIVVVASICVFTIGSEGQVFATSAIRGVRFLQILRMLHVDRQGGTWRLL
GSVIYLHRQELITTLYIGFLGLIFSSYFVYLAEREDGRKDFSSYADALWWGVITVMTIGYGDKVPQTWMGKIVASCFA
VFAISFFALPAGILGSGFALKVQQKQRQKHFNRQIPAAATLIQCLWRCHAAEPHSNSEATWRIHIHDPSSEETVFARM
ARRASLSLRKRRMSRLDSTSTVPNHLYRESMSSSMSYADDRLGRTQTSCLF

>Lymnaea stagnalis_aldehyde dehydrogenase family 3 member A2 isoform 2 (ALDH3A2)_mRNA

GTGTTGCGAGCTCGCAGACGACATTGACTTTATTTCAGAGAGTGACTGGAGTGTTAGTCCTTAGAGATTTTAATGATC
CTCAAGCTGTTGTTATGCAATATGTTTCTTGGATCTTTGTTTAATGTGCACTTCGTGAAGAAAGGAGAAGAAAATCGT
ACAGTGCGCCAAGAGTGAACAGTAACATCTGGGAGTTCACAGATTCAAAGTAATTTTTGTTACAGCAAGCCTTCAGAA
TGGGAGACTATGAACAGGTTGTCAAAGACCTCAGAGCGTCGTATGCTACTGGGAAAACCCGAAGTTTACAGTGGCGAG
TTGCCCAGCTGAAAGCCATCATTAAGCTTTTTGAGGAAAATGAATCTGCCATCTTTGAGGCTCTGTTCAAAGATTTGC
ACAAAAACAAGGCTGAGGCGGCCATAATGGAGACCATGCTCTGTGTCAATGATGCCGTCAATGCCATCAACAATTTAA
ATGACTGGACCAAGCCAGAGAAAGTAGCAAAGGGGGCCATATATATGATGGACAATGCTTACATCATGAAGGAACCCC
TGGGGGTCACACTGATCATTGGTGCCTGGAACTACCCTGTCCAGCTGACCATATTGCCTCTCATAGGGGCCATAGCAG
CAGGTAATTGTGCTGTCTTAAAACCTTCAGAAATGGCAGAGGAAACAGCCAGATTTCTTGAGGAGGTTGTGCCAAAGT
ACCTGGACAATGATTGCGTTCGTGTGATCAATGGAGGTGTCAAAGAAACAACTGCACTCCTGCAAGTCCGTTTTGACC
ACATTTTCTACACAGGCAACAGTGTCGTGGGCAAGATCGTCATGGAGGCGGCTGCCAAATATCTGACCCCGGTTATTC
TTGAACTGGGGGGCAAAAGCCCAGTTTATGTGGACAAGGGAACAGATTTGGAAATAGTAGCGAGGAGATTGAGTTGGG
GCAAGTTTTGTAACGCTGGTCAAACTTGCATCGCCCCGGACTATGTCATGTGTCCAAAGGACATCCAGGAAGGACTTG
TTGAGAAAGTCAAGTCAGCCATTGAAGAGTTCTATACAAAAGATCCGAAATCATCCGATAGTTATGGGAGGATCATCA
ATGAGAGACATTTTCAGAGACTACAGAGACTAAAGAAAGGAGCCACTGCTGCCTATGAGGGGGAAGATGATGAGAAAG
AACGTTATATTGCCCCTACAGTCATTCCCAATGTGAAACTGTCTGATCCAATCATGCAGGATGAGATATTTGGGCCAC
TTATGCCAATTGTGCCAGTAACTGACCACAAGGAAGCAATTGAAATCATCAATGGTCGTGAAAAGCCGCTGTCATTAT
ATGTGTTCACCAACAACAAAAGTATTGGTCAAGAGTTCAGGGACCGAACCAGCAGTGGTGCTCTTCTCATTAATGACA
CAGTCGTTCATGCAGGCTTGACTACTCTTCCATTTGGGGGTGTGGGCAACAGTGGTATCGGCTCCTATCATGGTAAAC
ATTCCTTCAATGCCTTCAGCCATGATAAACCAGTGATGGAGAAATCCTTGGCACTGGACCAGGTCAACAGCATGCGGT
ATCCACCTTACACTGAGAAGAAGCTTGGCTGGGTCAAATGGCTCATGGCCAAGAAGGTCAAGAGACAAGGATTTTTTT
CCTTCATGCCTTTTATTGCTATTGGAGTCATCATTTCAATGATGTTCAAAATTGTTGGAGTGGGAGCCGAGACCCTTG
AACAGAAAAAGAACATGTGAAGTGTTTTATAAGAAACTGTTGACTACATGATAAAAGACCAATTCTTTTGTTGTTAGA
GCCAAACTTTGTTTTCACAAAATGTGATTGACCTCTGTGTAGTAATATTATTACATTGAGGGGCATGCCCTACTTAGC
ACATACATGTTACATACACTTGTAACATACACTTGTATGTAAATGTTTCTGGTGTACTTTTAAAGAAGTAATTCTAAA
AACTTTTATTGTGATGGAAATAGCTCTTTATATAATCATTACTGTTTGCCCATCATTCTTTGTTGAAATTATTTTTAA
ATTTTCTGTTTAATTTTTTAAATTATGTTTGAGACCCACAACCCATATAGTGGTTTCCCGTTAGACATAGTATAATTA
TAGACTTTAAGAAAAAATATTTCACAGCCTCATTTATGTTAATGACAAGCCTTATCAAAAACAACTGTTATCAAAAACA
ACTGTTATTTATCTATCAAATATAATTGTTCTTCACTGACTGTGTTTTACGTTAAAATTAAAAACAACCATAATGTCC
TCAGCGCTCATGACATATAGCAAAAAACTGTGCCTCTGTTTTTTTAAACTGATTTAATATATCTTTAAAAAACCATAT
GTACATACTAACTCTAGCCCCAGCATGAACAATTCATATTGTCATATTGTCAAAGTGTACATTTATTTGTGGCTTAAT
TGATAAGGAACTCTTGAAGTCTGACACCCACTAACCTATTCAAAGAAATTTAGAAATTAAAATGACATTGTTAAATTA
TGTGCATTAGCTTTACAGGTAAAATTAATTTTTGAATTTTGTTTTTGCATGTCAAGTCCTTTTCTAATTGATGACAAA

TGAATATGATTTGCTTTTGACCATTTTATAATCGTCACCTATGGCTTAGGTCAAGGTTTCAGGACGGACAGCTTAATG
TGACTAAGTTTTTAAAATGTTGCCTGTGAGAAGGGTTCCTACTCCAAGACATGCAGACTTTGTAGTCACACCAGCAAG
TAACAGAGGGTTAATTATCACAAGTCCCAATGGAAGTCCCATGCATTGTCTGAGTTTCACATCATTACTGGACACTTG
AACTTAAAAATTTATTTTTACTGCAATTCGTCCATTGAAAACAATTATTATAGCCCTCTTACTCACCTTGCTTAAGTT
CTTCTTGTACAATTTTTTTTTTTTTTTTTCTTCTTGTGTTTTTTAGAACTCTGGTGTTAATGTTTTTTTTTTTTTTTT
TTTTTTTTTAACTGGCATTTTGAAATCCTTTTACATTTTTTTACAGTTTTTCTTTTCATGAATTATTATTAAAATAAT
TATATCTTTGTTGAGGCAATGTTGTGTCATTGTTTTATCGGCTCTGTTTCCTTTGTTTTTGAGTATGATTTTTTTTTC
CACAGTCGCTTTGTATTATAATAATAATATTAATAAAGCTGTTTCTTCAAAATAATTTTTTTTTT

>Lymnaea stagnalis_aldehyde dehydrogenase family 3 member A2 isoform 2 (ALDH3A2)_protein

MGDYEQVVKDLRASYATGKTRSLQWRVAQLKAIIKLFEENESAIFEALFKDLHKNKAEAAIMETMLCVNDAVNAINNL
NDWTKPEKVAKGAIYMMDNAYIMKEPLGVTLIIGAWNYPVQLTILPLIGAIAAGNCAVLKPSEMAEETARFLEEVVPK
YLDNDCVRVINGGVKETTALLQVRFDHIFYTGNSVVGKIVMEAAAKYLTPVILELGGKSPVYVDKGTDLEIVARRLSW
GKFCNAGQTCIAPDYVMCPKDIQEGLVEKVKSAIEEFYTKDPKSSDSYGRIINERHFQRLQRLKKGATAAYEGEDDEK
ERYIAPTVIPNVKLSDPIMQDEIFGPLMPIVPVTDHKEAIEIINGREKPLSLYVFTNNKSIGQEFRDRTSSGALLIND
TVVHAGLTTLPFGGVGNSGIGSYHGKHSFNAFSHDKPVMEKSLALDQVNSMRYPPYTEKKLGWVKWLMAKKVKRQGFF
SFMPFIAIGVIISMMFKIVGVGAETLEQKKNM

>Lymnaea stagnalis_copper-transporting ATPase 2 isoform-a (ATP7B)_mRNA

AGAATGACAAGCAGATAACAATAACAACAAAACTTGCGCCTATGTTTTCATTATTTGAGAAAAGTTATTTGTATAAAA
ATTCTTTGATTTGAAAGACTAAGACATCCTAATCGTAAGAAGCAGACATCATTTCATCATGGCTATGAGAAGGAAAAC
TACAATTGGCATTGATGGCTTACACAATGATGAAAGCATTAAAAAATTTAAAGACATGATGTTTCACCAAGATGGAGT
TTATTCAGTTGAAGTGAGTTTTGGTACAAAGAGGGCTGACATTCATTACAGTCCACTGAAGACAAGCCCACCCGCCTT
AGCTGAGCTGGCCACAAGCAACGGCTATCCAGCTATGGTGAAGGAGATTGAAGAGGCCGTCCCTTTGGTTCAAAGGGA
TGACCTGGAAGTGATGATTTCTGTGGAGGGCATGACCTGCATGTCCTGTGTCAGGAACATCGAGGGCACCATCTCCAA
GAGACCTGGGGTCAAGGTCATCTCTGTTTCACTGGAGAAAAAAATGGCTAAACTGGTTATCAGTCCTTCTCAGATTAC
ACCCGAAGATGCCACAGCGGCGATCGATGACATGGGATTCGATGCTAAATTAGTCAGCCCACTGTCCGCCCAGGCCGA
TATGGCGGTGACCACCCTGAGGGTTGAGGGGATGACCTGCCAGTCCTGTGTTAAGAACATTGAGGGTACGATATCCTC
CAAACCCGGGATCATTGACATTAAAGTCAGTCTCGCTGACCGACAGGCACTGGTGACCTATAACCCCAAGGTGACTAA
CCCCACAACTGTAGCAGAACAAATAGACGATATGGGGTTTGAAGCCTCTGTGCCAGTGGACCACTTGGCAAACGAAGG
CTTTGTCTCTGGGAGCTCACAAAATCTCAATGAAAATAGCTTGTTTGCCTCCAAAAAGAAAACTGTCTGCATTGATAT
CCAAGGGATGACATGTAACTCTTGTGTGAAGAATATAGAGGGCACTGTGGGTAAACATCCAGGTGTGGCCAGTATAAA
GGTGTCACTGGCCGACCAAATAGGAATCATAGACTACTATGCTGACAAGATAAGTCCAGAAAAACTTTGCAATCTCAT
CGATGACATGGGATTTGAAGCATATCTTCCAGATGTCACTAAGGTGAGGAAATCCAAGGTAAAGGTCATCCGTAGTGC
AAGTGAAATAGACTTGAAAGCTTCTCCACCACCTGCAAGGAAGATAGAAACACCTGTAGATGATGAGTTTGAGAGATG
CTTCCTCAATATCACTGGCATGACGTGTGCTTCCTGTGTTGCAACTATAGAAAAGAATGTCAGGAAAATGGATGGTGT
TCACAAAATTCTGGTCTCCTTGATGGCACAGAGAGCTGAGGTGACCTATGACCCTGCTTATGTTCTGCCAGGCCAGAT
CGCTAACAAAGTTGAAGACTTGGGCTTCAATGCATCAGTGATTGAGGGAGAAGCAGTGGGGCACGGACTGTAGAGCT
AACGATTACTGGGATGACATGCAGTTCTTGTGTCAATCGCATTGAGACGGACATTAAGAAGAAGAGAGGCATCCTATC
AGCATCTGTGGCTCTTGCCACCAGCACTGGGAAGTTCACCTTTGATTCAGAGCAGACCGGTCCAAGAGATATCATAGA
GGCTCTCAAGGGCATGGGATTTGACGCGCATCTCAGAACTGATGATGACAACAGAGCCTCGCGCTATGACCATCGAGA
TGAAATCAAAAGGTGGAGAACTTCTTTCCTGTGGTCTCTTATTTTTGGTGCCCCATCCATGGGCATCATGATGTACTT
CATGTTTGGTATGCCCGACACATCCCATGATGCCCCTGTCAACAGCACTGATGCCCACGGTGACCACATGAATAACAG
TGAAGTAGAAATGAAAATGACAGGATCCCATTACCGTCAAATAATGATCATACCTGGCCTAGACTGGCTCAACCTTAT
CATGTTTTTACTGGCTACACCTGTACAGTTTATCGGTGGAAGATACTTCTACATCCAGGCCTACAAGGTCATGAAACA
TGGGTCCACCAACATGGATGTACTGGTCGTCATGGCAACAACCATCTCCTACCTCTATTCCATTGCTGTGGTGATAGC

CGCCATAGCAACGGAGGAAGAATCCAGCCCCATGACTTTCTTTGAGACAACACCAATGCTGATGGTATTCATTTCACT
TGGTCGATGGCTAGAGCATATAGCTAAGGGTAAAACCAGTGAGGCCCTTGCCAAGCTGATCTCCCTCCAGCCGGCAGA
TGCTGTGCTTGTGGATGTGGATAAGAACTTCCAGATCCTCTCTGAGAGAACCATCAGCCTTGACCTTGTTCAGAGAGG
TGACATCTTGAAGGTCACACCCGGGGGTAAAATCCCGGTGGACGCCAAGGTCGTGTTTGGTCACTCGTCCTGTGACGA
ATCACTGATCACAGGGGAGAGCATGCCTGTGCCGAAGTCTGTGGGTTCTCCTGTCATTGGTGGAAGTATCAACCAAAA
TGGGATGCTGCTTATTGAAGCCACTTATGTTGGTGCTGACACAACACTGAGTCAGATTGTCAAACTTGTAGAGGAGGC
TCAGACATCTAAAGCTCCTATCCAGAATCTAGCTGACAAAATAGCTGGCATCTTTGTTCCTGTGGTCAGCATCTTGTC
CCTGGTAACACTTATAGTATGGGTGGCTATAGGATACTCTGATATTACATTGCTGGATAAACATTTTGATCCTAAAGG
TAACATTCCGTTGTCTGAGATTGTTTTTGAGAAGGCTTTCCAGTATGCCATTACTGTACTAAGCATTGCCTGCCCTTG
TGCCCTAGGACTGGCCACCCCAACTGCTGTCATGGTTGGAACAGGTGTCGGAGCAACCAATGGCATCTTAATTAAAGG
AGGAGAACCCCTGGAACTGACTCACAAACTGAAGGTGATTGTGTTTGATAAAACGGGCACCATCACCCATGGGGTCCC
CAGGGTGTCCAGAGTATCTATGTTTGTCAAAGAGAAGACCTGTTCCTTCATCAAGTTTCTGGCTGTGGCCGGCACTGC
TGAGACCAGCAGTGAACATCCCATAGCATCGGCCATCTTGAAATATGTTAAAGAGACTTTAGCTACTGAGAGTCTAGG
CAAAGTCTATGAGTTCTCAGCAGTGCCAGGATGTGGCATCAAATGTAAGGTGTCCCATGTGGAGAGCATTCTGAAGGG
TCTTGACATGGAAGGGGTCAACAACAGGAAAAACAGATATGGCAGTCAGCAGATTAAAATTGATAACTTCATGTCAGG
GGATCTGGAAACAAATAATCCACTGCATGATATGCCCATCGGAGCAGAGGCCTCCAGAGTCTATGAAGTGTTGATTGG
CAACCGTGAATGGATGCACAGGAATGGAATGATAGTCATAGACACCATGGATGAAGTGATGACAGAGCATGAGATGCA
AGGACACACTGCCGTGTTGTGTGCCATTGATGGCAACATTGTGGGAATGTTAGCAGTGGCGGATACTGTTAAGTCTGA
GGCCCACCTTGCTATCCACGAGCTTAAGAAGATGGGTTTGCAGGTCATTCTTCTAACAGGGGACAATCAGAAGACGGC
AAAAGCCATTGCCAAACAAGTTGGCATCACACATGTCTTTGCAGAGGTGCTCCCATCCCACAAGGTGAAGAAGATCAA
ACACTTGCAGTCAACGGGGCTGAAGGTTGCCATGGTTGGTGATGGTGTTAATGATTCCCCTGCCCTGGCCCAGGCTGA
TGTTGGAATCGCCATTGGCACAGGCACAGATGTGGCTGTTGAGGCTGCTGATATTGTCCTTATAAAGAATGATCTGTT
GGATGTATATGCAGCCATTAAACTGTCAAAAATGACTGTCAGGAGAATCCATATCAACTTTATCTCTGCTTGCGTTTA
CAACTTGTTAGGAATTCCTATTGCTGCAGGTGTTTTTACTCCCATTGGAATTGAGTTGCGTCCCTGGATGGCATCAGC
TGCTATGGCTGCATCTTCAGTTTCTGTTGTAGCAGCATCTTTGCTACTTAAAACGTTCCGTAAGCCTAAAAAAGAGGA
CCTGCTCACACCAGAGTATTACAAAAAATACTACCAGGACCAAGACTTGGATGAGATATCCATGCACAGGGGAGACGA
CTGGGAAAACCCAACTCGACAGCCCAGACAAAACAGCAATAGTTCCAAGAGAAGCTGGCGCAAAGCCAACAATCCTCC
AACTCCAGACCAGAAGAGTTTACTTGAACACGATGATGTAGACATAGAAATGAGTCCTATCACATAAATAAAAATAGC
CATAAGTATTCTGAGGCCAGTGGGGAAATAATGATTTGCAGCAAAAATTCAAACCACAAAAAATGTTTTCACTGACAA
AATGTTGGAACATCATGTAAATTCTTAAAAACTCAAAACCTTTTTTTTTTAAACAAAAATTTAAACCTATGAATCAAC
CCACTTATAACATCAACCATTGAAATCAGTTGGATTATGTTATAGTTTACAATTCCAATTACCTTTACCTTATTCTTA
GCAGTTAAAATAAAGGAAAAAAATGATATCTAAATTTAGGAATCCTTTTCATGACAAATTAGTGTGGTGGAACACACC
TGTTAATAATTAATCAATTTAAATAATTTGAATTACATGTTTGTATTGCCTACACTAGTTTTATTAACTAACAAGTGC
GTGTTTCTCATTCAATCCTATGGTTAAAAAAAAAACAACAAGACCAATAATTCTTTAACCACTTGCCATCAAACAAAA
TTCAGTCCAACAACTTCACTAATCTATGAAAACTTAATTTGTGAAATAATAATTCATTTTCTTCACTTACCAGTTAAG
TGGAAAATGTGCTGCTCTAGATAATGAGCAAATGTCAAAATTGATGTTGAAATCCAAATATGAAAAGAAATTTATATT
TTTCATAGAATAAATTTTTTTAAAATTTAAATGGTGCTTTGTTGGAGCAAATGTTAGTGACAGGAGGAGTGATTGATA
ATGGGGTGACTGCATCAGTGTATGTAGAATTTTTTTTTTGAGAATGGAGAAAATGTGCTGACTTTTATTTAAAACATT
ATGAAATAATAAAATGTATGGAAGTAGAAAAAGAATAGAATGGTGTTGAACTAAAAAAAAATTGAGATTAAAATTAAG
ATATTTTACCTTTGTTTGTGATGAAATACTACCTATGAGATATTTATTATTCAATATAATAAATTTACTTCACTCAAC
TCACTGCAATGATAGATAAACTTTAGATGACGCTAAAGGAAGGATTCTTTTTTAAATGCTGGCCTTTCATTTCTGCAC
CTAATGGTTTTGTATATTAGATCAAATTCTTTTTTTTTTTTAAATATAATTATTTTTGGATAAGTTGATAAGGGTATC
CACATAACTGTTTGAGTGGAAGACAATTTATAAAGGGTTAACACCATTCAAGATATTTTGTATGTAGATTTTTTTCAA
TCTTTGGTACTGATGTTCCTTGTGTATGAATAACTGCTGAATGCTCCATTTGACCAAATAATAACTTTTCCTAAATAT
TCTCCAACAATGATTTGTTTGTTGCACATGTCTTTCATATTAAATCCACTAAGCCAGTTGTGGGGGAAAAAAAAATTA
AAATCTTTCTTTAAAATAAAATAAATTTATTTTGAGATTTGTAAAAATATTTTTAAAATACACATTTTATTTCCATGT
ACGTATATGCATTTTAAAATATGTTTACATCAATTATAGTTTAAAAAAACACAATTAAAATGCAGTCATTTCAATGCC
AC

>Lymnaea stagnalis_copper-transporting ATPase 2 isoform a (ATP7B)_protein

```
MAMRRKTTIGIDGLHNDESIKKFKDMMFHQDGVYSVEVSFGTKRADIHYSPLKTSPPALAELATSNGYPAMVKEIEEA
VPLVQRDDLEVMISVEGMTCMSCVRNIEGTISKRPGVKVISVSLEKKMAKLVISPSQITPEDATAAIDDMGFDAKLVS
PLSAQADMAVTTLRVEGMTCQSCVKNIEGTISSKPGIIDIKVSLADRQALVTYNPKVTNPTTVAEQIDDMGFEASVPV
DHLANEGFVSGSSQNLNENSLFASKKKTVCIDIQGMTCNSCVKNIEGTVGKHPGVASIKVSLADQIGIIDYYADKISP
EKLCNLIDDMGFEAYLPDVTKVRKSKVKVIRSASEIDLKASPPPARKIETPVDDEFERCFLNITGMTCASCVATIEKN
VRKMDGVHKILVSLMAQRAEVTYDPAYVLPGQIANKVEDLGFNASVIEGEAVGHGTVELTITGMTCSSCVNRIETDIK
KKRGILSASVALATSTGKFTFDSEQTGPRDIIEALKGMGFDAHLRTDDDNRASRYDHRDEIKRWRTSFLWSLIFGAPS
MGIMMYFMFGMPDTSHDAPVNSTDAHGDHMNNSEVEMKMTGSHYRQIMIIPGLDWLNLIMFLLATPVQFIGGRYFYIQ
AYKVMKHGSTNMDVLVVMATTISYLYSIAVVIAAIATEEESSPMTFFETTPMLMVFISLGRWLEHIAKGKTSEALAKL
ISLQPADAVLVDVDKNFQILSERTISLDLVQRGDILKVTPGGKIPVDAKVVFGHSSCDESLITGESMPVPKSVGSPVI
GGSINQNGMLLIEATYVGADTTLSQIVKLVEEAQTSKAPIQNLADKIAGIFVPVVSILSLVTLIVWVAIGYSDITLLD
KHFDPKGNIPLSEIVFEKAFQYAITVLSIACPCALGLATPTAVMVGTGVGATNGILIKGGEPLELTHKLKVIVFDKTG
TITHGVPRVSRVSMFVKEKTCSFIKFLAVAGTAETSSEHPIASAILKYVKETLATESLGKVYEFSAVPGCGIKCKVSH
VESILKGLDMEGVNNRKNRYGSQQIKIDNFMSGDLETNNPLHDMPIGAEASRVYEVLIGNREWMHRNGMIVIDTMDEV
MTEHEMQGHTAVLCAIDGNIVGMLAVADTVKSEAHLAIHELKKMGLQVILLTGDNQKTAKAIAKQVGITHVFAEVLPS
HKVKKIKHLQSTGLKVAMVGDGVNDSPALAQADVGIAIGTGTDVAVEAADIVLIKNDLLDVYAAIKLSKMTVRRIHIN
FISACVYNLLGIPIAAGVFTPIGIELRPWMASAAMAASSVSVVAASLLLKTFRKPKKEDLLTPEYYKKYYQDQDLDEI
SMHRGDDWENPTRQPRQNSNSSKRSWRKANNPPTPDQKSLLEHDDVDIEMSPIT
```

>Lymnaea stagnalis_ubiquitin-protein ligase E3A (UBE3A)_mRNA

```
AATTTATAAACTTATTTATGACACGAGGGAAAATGTCTCAAGCCAGACTTTTGCATAACTGAACTTAGACAACAAAAT
CCAACATTTAAAAATCTGTCAAATAGTAACTGTCTGAGATAACGAGAACATACGAGATATTAACACACAAAATGGCGA
TCTGATGTACGACATTGGTTATTGTTTACTATGACTTTACAACTAGAAATACAATAACTAATATCTGAATACTACTAC
TAATCTGTTGAATTTCGTTGTTTGCTTTCGCATGAGTTCAATTCAGAACACCGGTTCACTGGACGGACAAGAGTCCGC
AAGTGACCTGACAGTTGAACACTCTCTGAGTGGTAAAGTGCCAAAAGTGTCTGAATCTACAGAAAATATGTCCAAACG
TGGTGCAGCCCAACAGGTGATATTTCAGTACTTCAACCAGCTCACAGAAGGCTGCGGCAATGAATCCTGCACTAATGA
AAATTGTGCCTCTAGTAGGACATTCCGGTTTAAAGAGAGTGATAACAACACTTTGGCCCTAGAGGCAATAACTCTGTC
TAAGGCTAAAGCCACTTTATGCGAAAGGCGTCCAAGAAAAATTTCTCGTCTTCCTACAGAGCAAGAAAATGGGACATT
GGACACTTCTGTTCTGGTTGCATCCAAGGAATTGGGTATGAGAACTAAAAGCAAAATGTTATCACCAACTGCATCATC
ATCAGTTGATGTTGGGTCTTCACAATCACCTCATGGCAGCAACTCTGAAATTAAAGAACCCAACTACTTAACAGAAGA
GAGACTAAGTGCTGTAATTCATGATTGCCAAGCCAGTAACGAATGGCTTAAGCTCATCCACCTCATTGGCTCTGTTTT
CAACAATCCTGAATCTATTATTATGAGTTTTCGGAAAAGCTACCCAGTTTCAAGCCCACCAAAAGTTAACTTGGTCTC
ACCTGATGATTCTAGTTCACCACCCATCAGTCAGCCAGGGATAGAGTCTCCAACGGTTCCTACAGTGAGTAGCATAAA
GGATCCTGATTTAACAGTTGACCTTCCAGCCTTAAGACGGGCTTTTAAGCTGTTGATGGATGTGCCAGATCAGCCTTA
TCAAGGTGCTCTGATCAATGCTCTGACAGCTCTTTCCCAGACACTGGAATATGAGACAAAGTACAATAAAGTGTTAGA
GAGACAAACAGATTACATTAATGTTTTTATTGTCGTCATGGAGATACCAATGCTCCATTTCCCAGAATATCTTGAAAC
TGCTTTTCCGGGAATTAACAAAGTTATAGGTTTGTTACCTGTAAGTGCTCAAGCTAGACTAGCCAGAATATGGTCGAC
TTTTGATCAAAGTCGTCTCAAAGACATGGTGCAATCATTACAACAATTGATTACAGTCAAGGTTATAAACTATGAAGG
CAGATGGTCAGCAAACTTCCGGCCAAGTGATGACTCTGCCATCACAAGTGCCACTCGTGTTCTCAAGATATTGTACTA
TGCCAGCATGCTTGGAGGACACATGGACAGCACTGAGCTGGTGGAGGAGGAGAGACGTTTAAATGACTCAGAATCACT
TCTAGAACTCATGCAAGGCGCTTTTGGCTATGAACCTAAAGAATCATCTCCTGTGAAGGAAGATCCCCTTGGAAAAGA
AGTGGGGGTTCAAGTCATTAATTGCAGGGAACCCCTTATACCTTATGAGGACTTTATCAATGAACCACTCAATGACAA
CTTAGACATAGGTGTGGACTACACCAACCACAGGCTAGAACCAGAGAACAAGTTTTCTTTTGTGCCCTACTGTTTCAT
ACTAACAACGGCGTCGAAGCACACCAGCATGTACTATGACAACCGCATCCGCATGCTGCATGAACGGAGAACAGCATT
TGTACAAACGTTGGTGCACGGTGGACCCCCAAACCCATTCCTCAGGGTTAGAGTCAGGAGGGACCACATCATTGATGA
TGCTCTTGTTAACTTGGAGATGATTGCTATGGAGAACCCTAGCGACTTACGCAAACAACTGTTTGTTGAGTTTGATGG
AGAGCAAGGTCTTGATGAAGGAGGTGTTTCCAAAGAGTTCTTTCAACTAATAGTTGAGGAATTGTTCAATCCTGATAT
AGGTATGTTCACATACAATGAACAATCTCATCACTTTTGGTTCAATTCTCTGTCGTTTGAAAATGATGCACAGTTTAC
```

ATTGATTGGTATACTTCTTGGATTAGCCATCTACAACAGCTGTATTTTAGACATACACTTTCCTATGGTGGTCTATCG
GAAACTCATGGGCAAAAAAGGAACGTTCAGAGACCTTTATGATGTGGATCCGACTTTATTTGCAAGTTTAAAGGAAAT
GCTGGAGTATAAGCTTGAGGATTTTGAAGAGGTGTTTGATCAGACTTTCCGGATTGGGTATAGTGATGTGTTTGGTAA
CAATCATACATATGATTTGAAAGAGAATGGAGAGTCAACAATGGTCTCTCAGGAAAATAAACAGGAATTTGTTGACCT
CTATGCCGACTATTTGCTGAACAAATCCATTGACCAGCAGTTTAGAGCCTTCAAACGTGGATTTCTCATGGTGACTTC
AGAATCCCCTCTTAAACAGTTGTTCCGTCCAGAAGAAATTGAAATGTTGGTTTGTGGGAGTCAGATATTTGATTTCCA
TGCATTAGAAGAAGCTACAGAGTATGATGGCGGTTTTACAGATGACTCTGCTACAATTAGAAACTTTTGGACTGTGGT
TCATGCCATGTCAGAAGAGGATAAAAAAAAGCTGCTACAGTTTACCACAGGGACAGATAGGGTACCCGTCGGAGGTCT
TTCAAAGCTCAAGATGATCATTGCCAGAAATGGTCCTGACTCTGACAGACTGCCCACATCCCACACTTGCTTCAACGT
GCTACTGCTCCCAGAATACCCCACTGTGGAGAAACTCCAGGACAGATTGTTGAAAGCCATTAACTACTCCAAAGGCTT
TGGAATGCTGTGATCTTTGTGTTTGGAACCTTGATTATTTAATTGAAACTTGTTTCTTGCTTGTAATGTTAATCTCCT
CGTGTCTCAAATTCACGAGTGTCCACATTGCTCATGTTAGTCAAATCTGTTCATTTTTTTTTGTGTGGAAGAGTTTGT
TCCTCTCTTATGCATCTTTCATTTTTCAATTGTATTTTAATTTTTTTGAAGGGTTTTAACATAATTTTTTTCCCCTGC
CTGCTTGCAGATTAAGGGGGCAAAAAAATGGAGGAAAAAAATTCAGAAATTACACTGGTGCAAATGATCTAGAGAGTC
TACTGGATTAGTCATTGCCTGTACACAACTCTCTCTTAAATGAGTTTTGCTGTTCTTTATTAATTAATATACACAGCA
TGCTGAAATATAAACAAATAAGAGAAAAAATAGAATCAGTTAAAAAAAATTAACTCTTGATTTCAGTCCTTGTTCCTT
GGTTAAAATTGTGAAAATAAATAAAATAAAATTATATAAATGATGGAAAAGATGTGCCATCTTTGTCACTATAAAGTA
TATTTTTAATGTACCGGTACTGTATGTAACTTTTAATATTTTGAAGCTGTTTGGAATGAGTCCAAATTCTTTGAAACA
AATAATAAAAACAAAGTGACGATGTAGTCTGCTCAGTCGAAAGCTGCCAAACTGACGATGTAGTCTACTCAGTGGAAA
GCTGTCACCAAGCCAAATACTATTCAATAAATATGTTTGAGTACTTATGGTGTGGATTTATCAAAATTATGTGTCATC
AGTCTAACAGTTTCTACTGTCTAACAGTGTCTACAGTCTAACAGTGTCTACAGTCTAACAGTTTTTACTGTCTAACAG
TGTCTACAGTCTAATAGTGTCTACAGTCTAACAGTGTCTACTGTCTAAAAGTGTCTACTGTCTAACAGTGTCTACAGT
CTAACAGTGTCTACTGTCTAACAGTGTCTACAGTCTAACAGTGTCTACAGTCTAACTGTCTACTGTCTAACAGTGTCT
ACAGTCTAATAGTGTCTACAGTCTAATAGTGTTTAACAGTGTCTACAGTCTTTTAACAGTGTCCTTTCTTTTCAGGAA
AATTAGGGAAGGGAAGTAAGCAAAATTGTCTGACTGAAGTTGTTGCTGGACCTTATCAGTAATATCAGTAGCTTTCAG
ACTTTAAACCTGCACACAGAAAGTCTGAGAAAGTGACTTAGTTTAAAACTATCATTTTAAAGTTTTCTTACCCTGGCA
AAAGCAAACCACTACTAATTTGCCAAAAGTTGGAATTTAAAAAAAAATTAAATTAAAAGATAAGACATATCTATGGAA
ATGGAAGCTCATCAGTTGTAGGTTAGCCCATCAGTTGTAGGTAGCTTATCAGTTGTAGGTAGCTCATCAGTTGTAGGT
AGCCTATCAGTTGTAGGTAGCTTATCAGTTGAAGGTAAAAATGTTGCTTTTTAAATCCAATTCCTTTGTTTCAGAAAT
ACCGAGCTGTTTGCAATAGTTATTTTATATCTTTAAGTTAAAAAAAAAAAGTAAACATTTTTGCTGATTTTTTTCTCT
CTAGAAGATAACCTACTGCCTGCATTACCCCCTAACCCTGTGAAACAATGGAATAGTATCTAAACTAGTGTCCCGAGC
TTGACCTGGCTGGGACTTGAGTTGCAATCTGGTCCTATGTTGTAGATATGCTGAATGCCACGGCCCAACCCAGGGAAC
TTCTTGCCAAAGTACAAGATAGGGTCGTGAAGAAAGATTAAGCCTAGCCTGCAGCTTGTGCACCTCCCCCACCCCTGT
GTGGATTAGAATAACCTTAATTTTTTTGTCAAGAGCTACTCATTCAATACTAAAGTACAGATGTGAAACCACTGTGTG
GCAACTAGTCCTCTATCACAGAAAATATGCTCTTAGAGTACTACTTCCTTGTTACCACTATTACTTACTAATGACCCA
GTATCCCACAAGGTGTTTGATTTACTTGAAGGTTCAAATACTGTGGTGAGAGATTGACTACCGGCACCTTAAACTGAT
GTTAATGAGAGAAATGTCAAGGCTGATGAAGTGCAAAGGTGAATTTTTTTTCTTTCCTATCCAGAGAAGCAAAATGAA
AACTTGAATTATTTGATGTTACGGATGCTACGGATGTTACAGATGCTACGACACTTAGAGCACTGGTTTTCATAAACC
GAGGCAGAATGGCATACATCCATGATGCAGTACCCGTAGATGAAACAAGTTAAGCAAATTGAAGTATGCTAACATGTT
CTTACTCTCCAAACCAAAGCGTGCTGTCATAGAATTCTGCCATCTTTAGATTAAACTTCAGCTGCTGGTTTCAGTGCA
TAGAGCCCACCAAATATGTCACAACAGATTGTGGAAGAGGTCACCATCTATTTCATGATTAGCTAAGCCCATAGAGCC
CACAAAATATGTCACAATAGATTGTGGAAGATGTCACCATCTATTTCATGATTAGCTAAGCCCCTAGAGCCCACAAAA
TATGTCACAATAGATTGTGGAAGATGTCACCATCTATATCATGATTAGCTAAGCCCATAGAGCCCACAAAATATGTCA
CAATAGATTGTGGAAGATGTCACCATCTATTTCATGATTAGCTAAGCCCATAGAGCCCACAAAATATGTCACAACAGA
TTGTGGAAGATGTCACAATAGATTGTGGAAGATGTCACAATAGATTGTGGAAGATGTCACAATAGATTGTGGAAGATG
TCACCATCTATTTCATGATTAGCTAAGCCCATAGAGC

>Lymnaea stagnalis_ubiquitin-protein ligase E3A (UBE3A)_protein

MSSIQNTGSLDGQESASDLTVEHSLSGKVPKVSESTENMSKRGAAQQVIFQYFNQLTEGCGNESCTNENCASSRTFRF
KESDNNTLALEAITLSKAKATLCERRPRKISRLPTEQENGTLDTSVLVASKELGMRTKSKMLSPTASSSVDVGSSQSP
HGSNSEIKEPNYLTEERLSAVIHDCQASNEWLKLIHLIGSVFNNPESIIMSFRKSYPVSSPPKVNLVSPDDSSSPPIS

QPGIESPTVPTVSSIKDPDLTVDLPALRRAFKLLMDVPDQPYQGALINALTALSQTLEYETKYNKVLERQTDYINVFI
VVMEIPMLHFPEYLETAFPGINKVIGLLPVSAQARLARIWSTFDQSRLKDMVQSLQQLITVKVINYEGRWSANFRPSD
DSAITSATRVLKILYYASMLGGHMDSTELVEEERRLNDSESLLELMQGAFGYEPKESSPVKEDPLGKEVGVQVINCRE
PLIPYEDFINEPLNDNLDIGVDYTNHRLEPENKFSFVPYCFILTTASKHTSMYYDNRIRMLHERRTAFVQTLVHGGPP
NPFLRVRVRRDHIIDDALVNLEMIAMENPSDLRKQLFVEFDGEQGLDEGGVSKEFFQLIVEELFNPDIGMFTYNEQSH
HFWFNSLSFENDAQFTLIGILLGLAIYNSCILDIHFPMVVYRKLMGKKGTFRDLYDVDPTLFASLKEMLEYKLEDFEE
VFDQTFRIGYSDVFGNNHTYDLKENGESTMVSQENKQEFVDLYADYLLNKSIDQQFRAFKRGFLMVTSESPLKQLFRP
EEIEMLVCGSQIFDFHALEEATEYDGGFTDDSATIRNFWTVVHAMSEEDKKKLLQFTTGTDRVPVGGLSKLKMIIARN
GPDSDRLPTSHTCFNVLLLPEYPTVEKLQDRLLKAINYSKGFGML

Figure S2. Identified conserved domains (specific hits) within the *L. stagnalis* homolog protein sequences (query sequence) for *in silico* validation. Conserved domain search was performed with NCBI CDD/SPARCLE (Lu et al. 2020; Marchler-Bauer et al. 2017).

>*L. stagnalis*_klotho

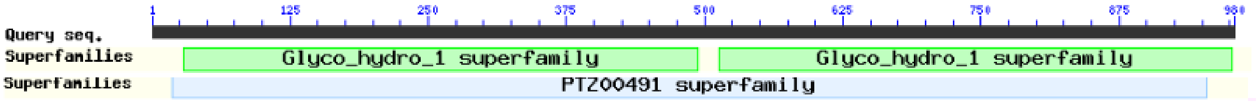

>*L. stagnalis*_ major vault 1

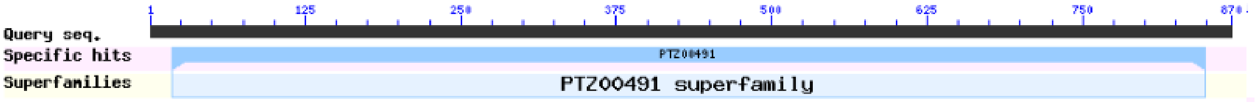

>*L. stagnalis*_ gelsolin

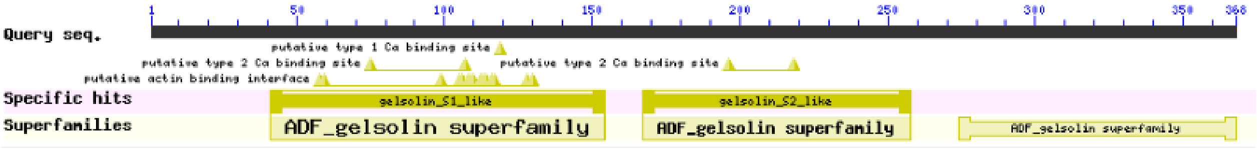

>*L. stagnalis*_ huntingtin

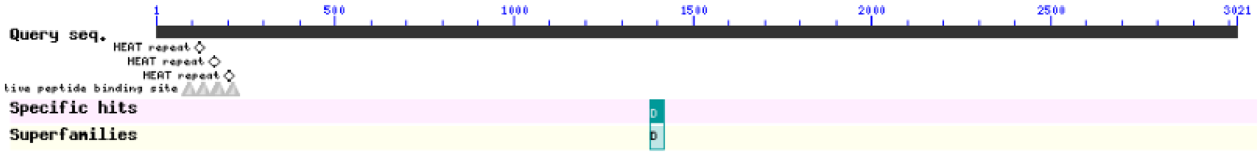

>*L. stagnalis*_ fragile X mental retardation protein

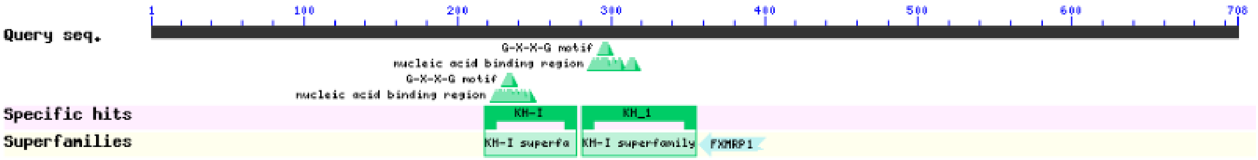

>*L. stagnalis*_ Parkinson disease protein 7/Protein deglycase DJ-1 (PARK7/DJ-1)

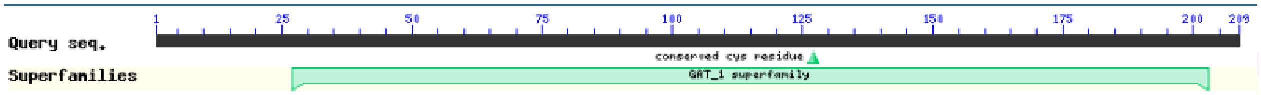

>*L. stagnalis*_ alpha-secretase (ADAM10)

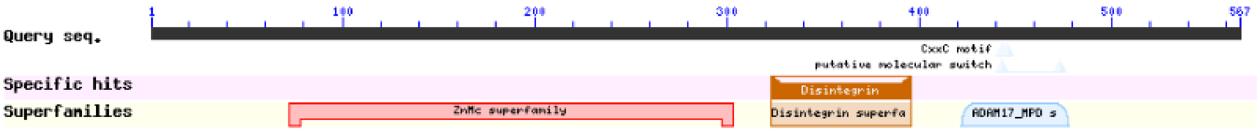

>*L. stagnalis*_ apolipoprotein E (apoE) receptor

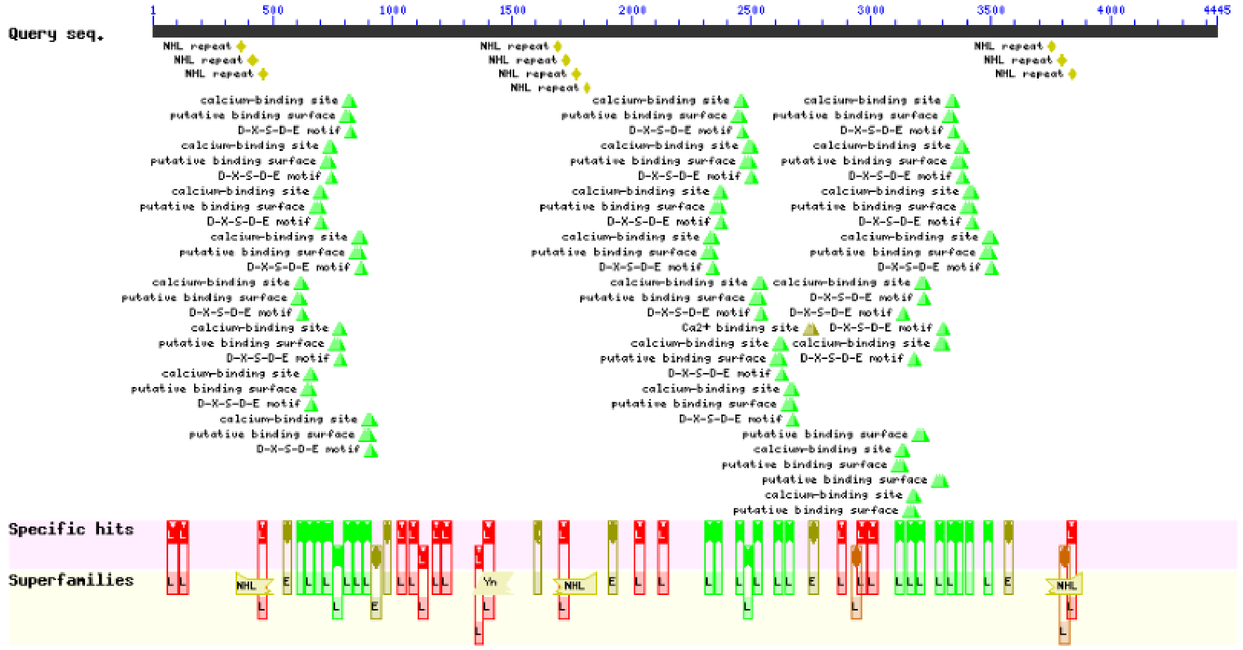

>*L. stagnalis*_ choline acetyltransferase (ChAT)

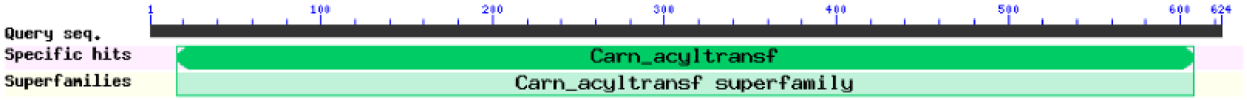

>*L. stagnalis*_ amyloid precursor protein (APP)

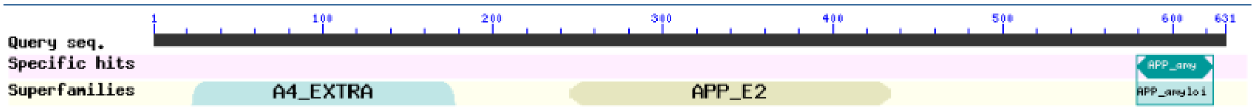

>*L. stagnalis*_ presenilin 1 (PSEN1)

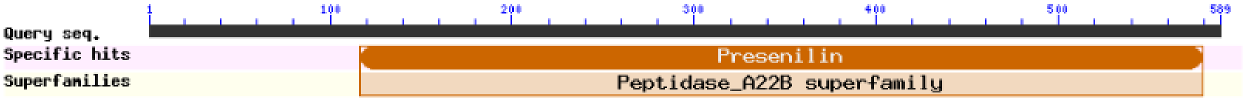

>*L. stagnalis*_ notch receptor 3

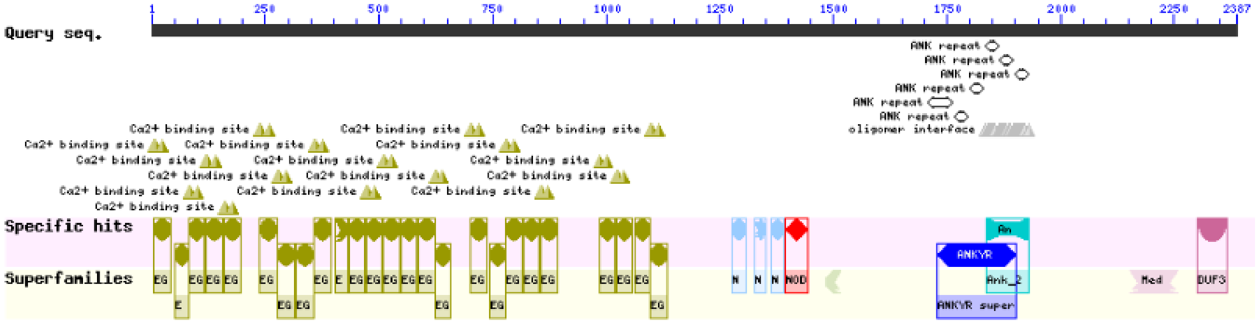

>*L. stagnalis*_potassium voltage-gated channel subfamily KQT member 2 isoform-e (KCNQ2)

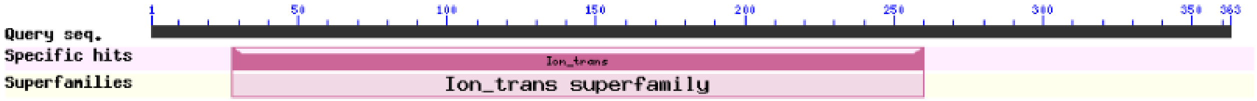

>*L. stagnalis*_ aldehyde dehydrogenase family 3 member A2 isoform 2 (ALDH3A2)

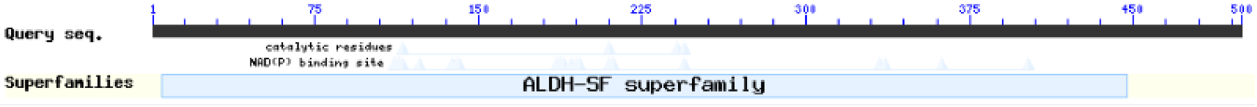

>*L. stagnalis*_ copper-transporting ATPase 2 isoform a (ATP7B)

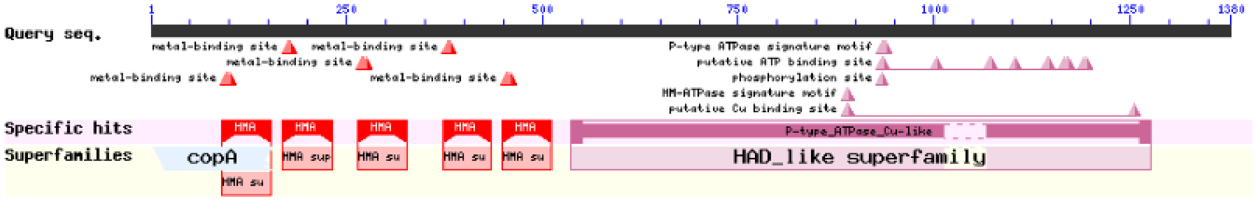

>*L. stagnalis*_ ubiquitin-protein ligase E3A (UBE3A)

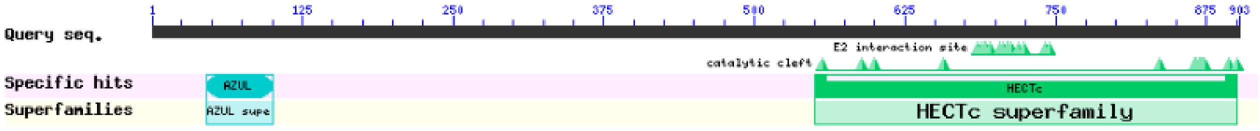

Lu, S., Wang, J., Chitsaz, F., Derbyshire, M. K., Geer, R. C., Gonzales, N. R., et al. (2020). CDD/SPARCLE: the conserved domain database in 2020. *Nucleic Acids Res, 48*(D1), D265-D268, doi:10.1093/nar/gkz991.

Marchler-Bauer, A., Bo, Y., Han, L., He, J., Lanczycki, C. J., Lu, S., et al. (2017). CDD/SPARCLE: functional classification of proteins via subfamily domain architectures. *Nucleic Acids Res, 45*(D1), D200-D203, doi:10.1093/nar/gkw1129.

Figure S3. Sequence comparisons between some chosen sequences from *L. stagnalis* and *A. californica*.

Fragile X mental retardation protein

```
#
# Aligned_sequences: 2
# 1: Lymnaea stagnalis
# 2: Aplysia californica
# Matrix: EBLOSUM62
# Gap_penalty: 10.0
# Extend_penalty: 0.5
#
# Length: 730
# Identity:     550/730 (75.3%)
# Similarity:   609/730 (83.4%)
# Gaps:          40/730 ( 5.5%)
# Score: 2795.5
#
#
#=======================================

Lymnaea            1 MEDLSVEVGGSN--GVYYKAYLKSFYEDEVLVSFENNWQADKRVKLTNVR     48
                     |||||||:.|::  |.:||.|:|.|:|||.||||||||..|:.||.:.:|
Aplysia            1 MEDLSVEISGNDGYGTFYKGYIKCFFEDEALVSFENNWLPDRTVKFSLIR     50

Lymnaea           49 LPPKSGATKPEFREDERVEVF--GKVKDEEGLAWYPAKIKMLKGEFAVVA     96
                     |||..|..||||||||:|||:  ||.||:||.|||||:|||||||||||.
Aplysia           51 LPPTPGQAKPEFREDEKVEVYTKGKDKDDEGPAWYPARIKMLKGEFAVVD    100

Lymnaea           97 SPWDANDILPLDKIRSVNHNPPITKESFFQFVLEVPPDLREGCQEELAIQ    146
                     .|||.||||||||||.||.||||||:||.||:||||||||||||||:|||
Aplysia          101 FPWDQNDILPLDKIRMVNLNPPITKDSFCQFLLEVPPDLREGCQEEVAIQ    150

Lymnaea          147 EFRKHIGGAMVSYNPEDKSLHVLSTSPSVIKRASMIGDMFLRNMRQKVLL    196
                     |||||||||:|:|.|||:|:||:::..:|:||||||||||||||||||||
Aplysia          151 EFRKHIGGAVVTYCPEDRSIHVVTSDQTVVKRASMIGDMFLRNMRQKVLL    200

Lymnaea          197 KQRTEEAAKKLQSTKIRSGYMEEFQVRDELMGLAIGTHGANIQQARKVDG    246
                     |||||||||||||||:||||||||||||||||||||||||||||||||:|
Aplysia          201 KQRTEEAAKKLQSTKVRSGYMEEFQVRDELMGLAIGTHGANIQQARKVEG    250

Lymnaea          247 ITGIELDEGSCTFKVYGETQEAVKSARGLLEFSEETFQVPRDLVAKVIGK    296
                     ||||||||.||||||||||.|||:||||||||||||||||||||||||||
Aplysia          251 ITGIELDEASCTFKVYGETLEAVRSARGLLEFSEETFQVPRDLVAKVIGK    300

Lymnaea          297 NGRNIQDIVDKSGVVRVKIEGDNEHETEREE--FFASFQGQVPFIFVGTM    344
                     |||||||||||||||||||||||||||||||  ||...:|||||||||||
Aplysia          301 NGRNIQDIVDKSGVVRVKIEGDNEHETEREEASFFCPMKGQVPFIFVGTM    350
```

```
Lymnaea          345 ESISNAKLLLEYHLDHLKEVEQLRQAKLEIDQQLKSLSGPQPGSYFPPPR    394
                     ||||||||||||||||||||||||||||||||||||||||||||||||||
Aplysia          351 ESISNAKLLLEYHLDHLKEVEQLRQAKLEIDQQLKSLSGPQPGSYFPPPR    400

Lymnaea          395 DRRWGYPEQFDDRRGRGSRGGRGTGRGRRWNTDRHGDDPSMPAAMVGDWS    444
                     :|||||.:.::||||||.|||||.|||||:..||||||||||.:||.|||
Aplysia          401 ERRWGYQDSYEDRRGRGGRGGRGAGRGRRFGMDRHGDDPSMPTSMVADWS    450

Lymnaea          445 AEVDEEKRQAGYLTDSILSGRGRAGGAYRRGSRGGGMRGGRGGLPPRG--    492
                     ||.:||||.|||||||||:|||..|| ||||:|||||||...||||||
Aplysia          451 AEGEEEKRDAGYLTDSILTGRGGRGG-YRRGARGGGMRGRGDGLPPRGPV    499

Lymnaea          493 -AGYDDDESRDPRSRRRMTDDDDTVLDNASVTSQDQDYDQQDRQRRPRRK    541
                      .||::|::||.|||||:|||||||||||||||||||||||..|||| ||:
Aplysia          500 MGGYEEDDNRDSRSRRRLTDDDDTVLDNASVTSQDQDYDQHARQRR-RRR    548

Lymnaea          542 KNRPRGNGGQASGTETDTSVSNFRGDRSRGRGGRGGGYNQRGHESDSGRG    591
                     ||||||:.|..|||||||||:||:|.:  ||.||||||...|      |.|
Aplysia          549 KNRPRGSYGTHSGTETDTSISNYRSE--RGSGGRGGGRQGR------GAG    590

Lymnaea          592 GYRG-NESDSGHPPSVSASGVGRSSVSPGMQTVNGGGGDRPTKQEPPPKD    640
                     .|.| .::||...||.|.|..||||||||:||||||.|||..||:..||:
Aplysia          591 AYSGPRDADSAPVPSGSVSTAGRSSVSPGVQTVNGGSGDRSLKQDGGPKE    640

Lymnaea          641 QRDGRPPRDTRPRGSNNNSSVPPS------------GSTQPPKQMVGNHH    678
                     ||:||||||.||||||||||...|            |:.||     |:||
Aplysia          641 QREGRPPRDPRPRGSNNNSSATASATSGAGGNSSQLGTKQP-----GSHH    685

Lymnaea          679 SGSDSDSKLAKSKMNNNSAKAKEHIVNGAE    708
                     |||||.|||||:|:||  :|.|||||||.
Aplysia          686 SGSDSGSKLAKNKLNN--SKTKEHIVNGE-    712


#---------------------------------------
#---------------------------------------
```

#**Gelsolin**

```
#
# Aligned_sequences: 2
# 1: Lymnaea stagnalis
# 2: Aplysia californica
# Matrix: EBLOSUM62
# Gap_penalty: 10.0
# Extend_penalty: 0.5
#
# Length: 369
# Identity:     233/369 (63.1%)
# Similarity:   288/369 (78.0%)
# Gaps:           3/369 ( 0.8%)
# Score: 1256.0
#
#
#=======================================

Lymnaea            1 MAGRGLVKAKKYDWKDSNLALFGSDLEKNVKKASAATEVAWKGAGTQPGV     50
                       ..|||||||||||||||||||||:||||||.||..|.|||.||:|.|:
Aplysia            1 --MSGLVKAKKYDWKDSNLALFGSDVEKNVKKESAEQEPAWKNAGSQVGI     48

Lymnaea           51 QVWRIVQFKVTPWPKEDYGKFFSGDSYIVLNTYKEEGNDQLLYDVHFWIG    100
                     |:||||:|||.|||||||||||.|||||:||||||:..||||||||||||
Aplysia           49 QIWRIVKFKVQPWPKEDYGKFFEGDSYIILNTYKEQDTDQLLYDVHFWIG     98
```

```
Lymnaea          101 KESTQDEYGTAAYKTVELDTFLNDVPVQHREVQDHESDLFKSYFKTITIM    150
                     :.||||||||||||||||||.|:||||||||||.|||:|||||||:||||
Aplysia           99 RHSTQDEYGTAAYKTVELDTLLDDVPVQHREVQGHESELFKSYFKSITIM    148

Lymnaea          151 KGGAQTGFRHVEEEKYKPRLFHFSGQRKNVVVSEVPLCKDRIKSDDVFIL    200
                     ||||:||||||:.|:||.||...:|.:::|.|:||||.|:|:.:.|||:|
Aplysia          149 KGGAETGFRHVKPEEYKQRLLQITGNKQSVTVTEVPLNKNRVTAKDVFVL    198

Lymnaea          201 DLGKKIYQWNGRGSNKDERFKAGQFCQQLESERSGRAKADVLEEDTTDRS    250
                     |.|.:|:||||....|:|::||.|..||:.|||.|:...:|.::::.|.|
Aplysia          199 DNGLEIFQWNGEECAKEEKYKAVQVVQQIRSERGGKPSVEVFDQNSDDGS    248

Lymnaea          251 HLFYQSLTEDDSDDDSEFDAKDLQ-KELFRLSDSSGNMTFKVEKKGTVSK    299
                     ..|......:|.|||||::..|.: .||:|||||||...|:..|:|.|.|
Aplysia          249 TFFDHFNDNEDDDDDSEYEDNDNKTPELYRLSDSSGEFEFERTKEGRVFK    298

Lymnaea          300 SDFDTKDVFILDAKKSLFVWIGQGTTAGEKKLALQYAHEYLQKTDHPLIP    349
                     .||.:|||||.|.||.:|||||:..:..|.:.||.|||:|||.|.|||:|
Aplysia          299 DDFSSKDVFIFDNKKEVFVWIGKSASKSENQNALSYAHKYLQGTQHPLLP    348

Lymnaea          350 VTCLKEGQESRDFAAAIAA    368
                     :||:|||:|::.|.:||||
Aplysia          349 ITCVKEGKENKFFRSAIAA    367


#---------------------------------------
#---------------------------------------
```

#**Choline acetyltransferase**

```
#
# Aligned_sequences: 2
# 1: Lymnaea stagnalis
# 2: Aplysia californica
# Matrix: EBLOSUM62
# Gap_penalty: 10.0
# Extend_penalty: 0.5
#
# Length: 732
# Identity:     446/732 (60.9%)
# Similarity:   508/732 (69.4%)
# Gaps:         108/732 (14.8%)
# Score: 2348.0
#
#
#=======================================

Lymnaea            1 --------------------------------------------------      0

Aplysia            1 MDHRNGDETSCSSSTHSNNNSRSSSNTASNNSIISDAGGITKQTPFNQNS     50

Lymnaea            1 -------------------------------------------------M      1
                                                                      .
Aplysia           51 SSGQHPDQQEQDQQQQQQKRQSLNGGISNGGSGGLSSPANQKATVNGSNK    100

Lymnaea            2 GKTTECPFSPRKF----PLPKLPVPDLQGTMEKYLSLIKTVVSPQEYSRT     47
                     |.||....|...:    ||||||||:||.||||||||:|||:||.|:|||
Aplysia           101 GTTTRTNGSQVYYDLSQPLPKLPVPELQSTMEKYLSLVKTVMSPHEFSRT    150

Lymnaea           48 KYTVDEFCKPGGVGHELQEYLLKRQQCMDNWANEWWLNDMYLNVRIPVLI     97
                     ||.||||||....||||||:||||||.||||||:.|::|||||:::|:.|
Aplysia          151 KYIVDEFCKTQAQGHELQEHLLKRQQGMDNWANDLWIHDMYLNIQVPLTI    200
```

```
Lymnaea           98 NPNPAAVFPYQGFPSHREQIRFAAKFIRGMLDFKHLTDTRTLPIERCKYK    147
                     |.|.||:||||.|||.|||:||.|||.||:||||.||||||||||||:||
Aplysia          201 NSNAAAMFPYQAFPSQREQLRFTAKFARGLLDFKRLTDTRTLPIERCRYK    250

Lymnaea          148 EKGQPLCMDQHYRLFTSYREPGRERDVQRTDLGFMGREYIVVACNNQFYK    197
                     |||||:||:||||||||||||||||||.|:|.|..||::|:|||.|||||
Aplysia          251 EKGQPMCMEQHYRLFTSYREPGRERDVMRSDTGTPGRDFIIVACRNQFYK    300

Lymnaea          198 VDVQREGDELTEADICLQLSRVLNMAEQESNQSEPVGLLTSQKRTLWAEQ    247
                     |||.:||.||:||||.|||:||:..|:|..::..|||:||||.|.:|||.
Aplysia          301 VDVIQEGQELSEADIFLQLNRVMTSAQQSVDKPAPVGILTSQARNIWAEH    350

Lymnaea          248 RERLQLDATNRANLHILENCLFLLCLDKPTVPSRCGIQGGMLNDMTARTH    297
                     ||||..||||||||..|||||||:|||..|:||.....|||..||.||||
Aplysia          351 RERLLQDATNRANLFALENCLFLVCLDLTTLPSGRDGDGGMAGDMAARTH    400

Lymnaea          298 HIIHGQGVHNNSANRWMDKTIQVIVSEDGTCGINMEHSVAEGIALGHMIE    347
                     .|:|||||..|:||||||||:|.||:||||||:||||||.|||||...:|
Aplysia          401 QILHGQGVERNTANRWMDKTVQFIVAEDGTCGVNMEHSVVEGIALCSAVE    450

Lymnaea          348 HAFGVMGKEKIQDGPRDPNSLPHPKCLQWSLSAHSLADIEQAKESVDNMV    397
                     |||.||||:...|...||.||..||||||:|:..||.|||.|||:|..:|
Aplysia          451 HAFSVMGKDNFSDKICDPASLALPKCLQWNLTTQSLRDIEVAKENVARLV    500

Lymnaea          398 NDFDLTVFRFEGYGREFIKTQGMSPDAYIQLALQLTYYKIHGTLTSTYES    447
                     :||||.||||..|||||||.||||||||||||||||||||||..||||||
Aplysia          501 DDFDLAVFRFPRYGREFIKAQGMSPDAYIQLALQLTYYKIHGCSTSTYES    550

Lymnaea          448 ASVRRYRQGRVDVIRANSPPALTWIKAMLGQTEATEEDKLRLFTEAVHWQ    497
                     |||||||.||||.||||||.||.||||||||||||:|||.||.||||.||
Aplysia          551 ASVRRYRHGRVDNIRANSPAALAWIKAMLGQTEATDEDKRRLLTEAVQWQ    600

Lymnaea          498 QDYMLDTILGYGIDLHLLGLREAAKEMGIPTPEFFNDPSYKELNTFRLST    547
                     ||.||:||||.|.|||:|||||||.|:|:|||:.|.|.|||..|.|:|||
Aplysia          601 QDNMLETILGNGTDLHILGLREAAAELGLPTPDLFQDASYKAFNCFKLST    650

Lymnaea          548 SQVPTVSDYWMGYGAVVPDGYGCCYNPK-PDSIIFSVATFNTCQDTSSEM    596
                     ||||..||:||||||||||||||||||: ||||:||||:|.:|.||||||
Aplysia          651 SQVPMTSDFWMGYGAVVPDGYGCCYNPQAPDSIVFSVASFLSCYDTSSEM    700

Lymnaea          597 FAHSLESSLLQMAELCTYDP----ETATIKQR    624
                     |..|||||||||||:||.:.    :....|:|
Aplysia          701 FTQSLESSLLQMAEICTVESDVKVQNPNSKER    732


#---------------------------------------
#---------------------------------------
```

Amyloid precursor protein

```
#
# Aligned_sequences: 2
# 1: Lymnaea stagnalis
# 2: Aplysia californica
# Matrix: EBLOSUM62
# Gap_penalty: 10.0
# Extend_penalty: 0.5
#
# Length: 683
# Identity:     423/683 (61.9%)
# Similarity:   516/683 (75.5%)
# Gaps:          67/683 ( 9.8%)
# Score: 2232.0
```

```
#
#
#=======================================

Lymnaea            1 -------------------------MRHLFQVG-----ALF-ALIQVLFS     19
                                              :.:..::|     |:| .|:|||::
Aplysia            1 MNATLRPLTEYGKFEAEDLKYLLLGLDYTAEMGLVTNCAIFLGLLQVLYA     50

Lymnaea           20 ASLEDKYEPMVAFICERPAMHRGVNGWIADKSTDCLDRMEDILAYCKAMY     69
                     ||:|||||||||||||||||||||||||||||||||:||||||:||:.||
Aplysia           51 ASVEDKYEPMVAFICERPAMHRGVNGWIADKSTDCLNRMEDILSYCQRMY    100

Lymnaea           70 PDHNITNVVESSYLVTISDWPMGNAERQHPHRVRPFRCLVGGFQSDALLV    119
                     ||||||||||:||||||.||||.:|:|.||||||||||||||||||||||
Aplysia          101 PDHNITNVVEASYLVTIKDWPMTSADRMHPHRVRPFRCLVGGFQSDALLV    150

Lymnaea          120 PQHCEFDHRHDQTQCEGFAHWNVIADDACDKKGMHLESFGMLLNCNLGKF    169
                     ||||.|||||||..|:||:||||:||::|..|.||||||||||||.||.|
Aplysia          151 PQHCVFDHRHDQRVCQGFSHWNVVADESCRSKDMHLESFGMLLNCKLGMF    200

Lymnaea          170 SGVEYVCCPVETETKYHQPQTDDKPDSWVNTHDDMDKEEKTNASKSSTD-    218
                     |||||||||.:.:..||.||||||||.|   ||...:||.....|...|
Aplysia          201 SGVEYVCCPNQNKPNYHPPQTDDKPDDW---HDSSAEEEDEEEEKEGEDS    247

Lymnaea          219 -PAAATSSSS---------SSSEEVLIFVCSLLQEAD--ENTVDLYEAYL    256
                      |.|..|:||         |:|:..:     .:.|.|  |:.:|.|||||
Aplysia          248 NPTAPQSTSSEESSDSGATSNSDSAV-----TINEDDLEEDVMDFYEAYL    292

Lymnaea          257 RGQEFPQKYNNEHKKFVAARDRMKKNQQHKVTKLLQEWQAARDHVNEVRK    306
                     ||||||.|||||||||:||:||||||||||.||||:||.||||||:||||
Aplysia          293 RGQEFPSKYNNEHKKFLAAKDRMKKNQQHKTTKLLQQWQVARDHVDEVRK    342

Lymnaea          307 SDPKTADTMAKEITARFQNLYAAYEQEDDSEKEQLTSLHQQHVQAALNER    356
                     .|.|.||:::.||.:|||.|||:|||||.:||:||.:|||||||||||||
Aplysia          343 VDTKKADSLSGEIASRFQKLYASYEQEDAAEKQQLMALHQQHVQAALNER    392

Lymnaea          357 KRDAMDKYMRALEKGDADKIIKYLRAYIKAEEKDRMHTVNHFEHVKYSSP    406
                     ||||||.||:||||||.:||||.||:|.:...||||||||||||||||:.
Aplysia          393 KRDAMDNYMKALEKGDVEKIIKDLRSYSRRRRKDRMHTVNHFEHVKYSNA    442

Lymnaea          407 SQAKALQPHIKDHLSLTEKRIEQALQLLSRYPDIEAKNKPEIYEFLKRFE    456
                     .:|..:.|.|.:||.|:|:||:|||::||||||||.:.:||:.||:|||:
Aplysia          443 REALRIHPFIINHLRLSEQRIDQALEMLSRYPDIEVQVRPEVEEFMKRFD    492

Lymnaea          457 SIANSIRDVV--LPEIVEESESEESEEVTEVPQNI---VNNNDEINLDDN    501
                     :|||||::||  ||::.||.:::.:.:  |.||:.   |:.:|:|.:||.
Aplysia          493 AIANSIKNVVLPLPKVEEEPQTKSASQ--EAPQDASDSVSPDDDIRIDDA    540

Lymnaea          502 NDFDVSKQEIDTQGDDVVENEHDYEKKNAFVANRMEDTHHIQQGFVESAA    551
                     .|||:|.:::|.:||||.|:|||||:.:.|:|:||:|..|::|||.||||
Aplysia          541 GDFDLSSEQVDEEGDDVSEDEHDYERGSQFIAHRMDDKMHVRQGFAESAA    590

Lymnaea          552 TSSQVGSTIGIALGSVSVFVIIVVAIIMLKRNKTRQSVTHGYVEVDPSAS    601
                     ||||:|||||||||.||||||||||:.|:||....|..:.||||||||||
Aplysia          591 TSSQMGSTIGIALGGVSVFVIIVVAVFMVKRKNRNQYPSPGYVEVDPSAS    640

Lymnaea          602 PEERHLANMQMNGYENPTYKYFEVQNNPKA---    631
                     |||||:|||||||||||||||||     |.
Aplysia          641 PEERHVANMQMNGYENPTYKYFE-----KGEFV    668


#---------------------------------------
#---------------------------------------
```

#**Presenilin**

```
#
# Aligned_sequences: 2
# 1: Lymnaea stagnalis
# 2: Aplysia californica
# Matrix: EBLOSUM62
# Gap_penalty: 10.0
# Extend_penalty: 0.5
#
# Length: 626
# Identity:     372/626 (59.4%)
# Similarity:   436/626 (69.6%)
# Gaps:          82/626 (13.1%)
# Score: 1782.0
#
#
#
#=======================================

Lymnaea            1 --MSSHSKTSGAVFATDDSPTERTSLMSSFV-NEAARDGGSTSVFGPVPS     47
                       ...:|.||..|   |.||||||.|||:.. .|.|.:.|:   |.||  
Aplysia            1 MNPQRNSTTSVGV---DSSPTERTGLMSNLTGGEWAGESGN---FSPV--     42

Lymnaea           48 SPNTNRITTIEDGSATPAIPETSVIIPNANNTGQVSGAPRDSHRRRRRNP     97
                         .|.:..:|.:.|  :|||||:.|:.|:|  .:..|:....||....
Aplysia           43 ----RRYSAADDEATT--VPETSVVTPSTNST--TNQQPQSQRTRRGEGS     84

Lymnaea           98 SQRS-------------------------------AHQQQEEEEDEETLL    116
                     ..||                               :.||:||::.|||||
Aplysia           85 QNRSDGDGAAATRGGGAQPAAAARQEAGRGSGGAVSGQQEEEDDGEETLL    134

Lymnaea          117 YGAKHVIMLFVPVTLCMAVVVATISTVNYYTDTGTYLIYTPFHDKTDNTG    166
                     |||||||||||||||||.|||||||::.|||..|||.:||||||||::||
Aplysia          135 YGAKHVIMLFVPVTLCMVVVVATISSITYYTTKGTYFLYTPFHDKTEDTG    184

Lymnaea          167 TKIWQSLANSLIMLGGIVVMTIVLLLLYKYKCYKIIHGWLVLSSVMLLFF    216
                     ||:|||:||:||:||.|:||||||||||||:|||||:||||:||||||||
Aplysia          185 TKLWQSMANALILLGAIIVMTIVLLLLYKYECYKIINGWLVMSSVMLLFF    234

Lymnaea          217 FSYIYMEQILRSYNTPIDYITVAIIMWNFGVGGMFCIHWKGPLLLQQAYL    266
                     |||||:|||||:||.|:||||||||||||||||:||||||||||||||||
Aplysia          235 FSYIYLEQILRAYNVPMDYITVAIIMWNFGVGGLFCIHWKGPLLLQQAYL    284

Lymnaea          267 IMVSSLVALMFIKYLPDWTAWTVLGVMVVWDLVAVLCPKGPLRMLVETAQ    316
                     |.:|:||||||||:|||||.|.||||||||||||||||||||||||||||
Aplysia          285 ISISALVALMFIKFLPDWTTWAVLGVMVVWDLVAVLCPKGPLRMLVETAQ    334

Lymnaea          317 TRNEPIFPALIYSSTMVWLITMADDESSSKKGKRGTSQGTGVSGTSGGAR    366
                     .|||||||||||||||||.|||||.:.:.||.::...|.|..:..|  |.
Aplysia          335 NRNEPIFPALIYSSTMVWTITMADGDPNKKKNQKKKKQETTTAEES--AN    382

Lymnaea          367 GASEIHESD-DEGGFREHLSNGTNRANELTASSDSQTARRAVQALGEMSH    415
                     ||.....:| |:|||:||:.||.:|:..|::.|:|.:||.||.|||:||.
Aplysia          383 GAQASGGADEDDGGFQEHIQNGGSRSRGLSSGSESNSARTAVAALGDMSQ    432

Lymnaea          416 G-NPNPHRHIAGSSSDPVQNPTETSAVVPKKKKTPKPR-QRAAAPQENSA    463
                     . :|...|    :..|.|....||:.||  .:..|:|| ||||..|:|..
Aplysia          433 ADSPRQQR----AKVDTVAVDAETTVVV--NRNPPRPRAQRAATDQQNRQ    476

Lymnaea          464 EEDEENNIVRFQKFERKHICNYYLFPKLLTHHFILFAGGVKLGLGDFIFY    513
                     ..:.|.::...:..|.:                     ||||||||||||
Aplysia          477 RNESETDVASSEADEDR---------------------GVKLGLGDFIFY    505
```

```
Lymnaea          514 GVLVGKASSKGDWNTTLACFVAILIGLCCTLLLLAIFRKALPALPISLTF    563
                         |||||||||.|||||||||||||||||||.||||||||||||||||||||
Aplysia          506 GVLVGKASSNGDWNTTLACFVAILIGLCFTLLLLAIFRKALPALPISLTF    555

Lymnaea          564 GLVFNFATSALVRPFMDSLASEQVYI    589
                         ||||||||||||||||||||||||||
Aplysia          556 GLVFNFATSALVRPFMDSLASEQVYI    581


#---------------------------------------
#---------------------------------------
```

#**Huntingtin**

```
#
# Aligned_sequences: 2
# 1: Lymnaea stagnalis
# 2: Aplysia californica
# Matrix: EBLOSUM62
# Gap_penalty: 10.0
# Extend_penalty: 0.5
#
# Length: 3045
# Identity:    2384/3045 (78.3%)
# Similarity:  2698/3045 (88.6%)
# Gaps:          45/3045 ( 1.5%)
# Score: 12328.5
#
#
#=======================================

Lymnaea            1 MATIEKLIKAFEGLKVFQPNAQTVEDPKKKDQNLPTKKDKMLHCNIVADC     50
                       ||||||||||||||:|||||||..:|.||||||:||||||:||||:||||
Aplysia            1 MATIEKLIKAFEGLRVFQPNAQAADDSKKKDQNVPTKKDKILHCNVVADC     50

Lymnaea           51 MCSPNMRTIADFPKFLGIAMESFLTLCDDPEADVRMVADECLNRTIKVLL    100
                       ||:||||.:||||||||||||:||.|||||||||||||||||||||||||
Aplysia           51 MCAPNMRGVADFPKFLGIAMEAFLALCDDPEADVRMVADECLNRTIKVLL    100

Lymnaea          101 ETNLGRLQVELYKELKKNGPSRSLRAALWRFADMCHLIRPQKCRPYIVNL    150
                       ||||||||||||||||||||||||||||||||||||||||||||||||||
Aplysia          101 ETNLGRLQVELYKELKKNGPSRSLRAALWRFADMCHLIRPQKCRPYIVNL    150

Lymnaea          151 LPCVARICRREEEAVQDTLSMAMPKICSALMPFANDTEVKALLKSFLPNL    200
                       ||||||||||||||||:|||.|||||||||||||||||||||||||||.|
Aplysia          151 LPCVARICRREEEAVQETLSSAMPKICSALMPFANDTEVKALLKSFLPTL    200

Lymnaea          201 KSNSAVCRRMSASSLALICQHSRAPLSFFNYLIGVLLEMILPVDIDHEVP    250
                       ::.|||||||||||||||||||||||||||||:.|||||||||||:|:||
Aplysia          201 RATSAVCRRMSASSLALICQHSRAPLSFFNYLVSVLLEMILPVDIEHDVP    250

Lymnaea          251 TLLGVILCLRHSIPHLVPSNNKDQGLKDSFGYRESEAEQAIGEEKMVKIL    300
                       ||||||||||||||||.|:.|||.||||||||||.||||||..::::|||
Aplysia          251 TLLGVILCLRHSIPHLAPTTNKDLGLKDSFGYREVEAEQAIHVDQIIKIL    300

Lymnaea          301 QALLHYSGHSDHNVVTAALESLQQLLRTPPPILRKMLLTRGSISRTYIFI    350
                       ||||||:||||||||||:|||||||||||||.|.::||.:||||||:||.
Aplysia          301 QALLHYTGHSDHNVVTASLESLQQLLRTPPPALLRLLLKKGSISRTFIFS    350

Lymnaea          351 HDFEEEDQARIESVVDLTSMSDDANLDED-ADLGYTNSHPSARKSNTSGL    399
                       .||.||:||||:||.||....|::.||:| .|:  :....::..|:.|..
Aplysia          351 QDFNEEEQARIDSVTDLALTVDESGLDDDPGDV--SGQSVASGGSDNSTF    398
```

```
Lymnaea               400 MSEFEAKSATSQSRLAASLTESNVGYNELVNND---AILVTDGTDYSGVE    446
                          .|::...|.|||.  ::.:..::.|..::|..|   .|:|||||||||||
Aplysia               399 ASDYGTGSFTSQQ--SSDVEAASAGLTKIVIGDEGEMIVVTDGTDYSGVE    446

Lymnaea               447 IGDLNEERSEMSATSGMSHSDSIETLQSVRSISPHLAYTPPVQLLGHDMN    496
                          ||||||||||:||.|.:||||||||||||||:|||   ..|:||..||||
Aplysia               447 IGDLNEERSELSAASCISHSDSIETLQSVRSLSPH---PGPLQLPSHDMN    493

Lymnaea               497 GNPQIVVYPGEDAAPPLSPNPALGEPLEQINIEHESLQEDEVPLLFYLRL    546
                          ||||:.|...|::. |.|||||.|:.::||.:|.|||:||||||||||||
Aplysia               494 GNPQLPVEQEEESV-PASPNPATGDTVQQIEVEPESLKEDEVPLLFYLRL    542

Lymnaea               547 LCKRFLLTGVTDGLVTDKQVRVSLKSLALGCVSCSLALCPRLFLFKLCPT    596
                          ||||||||||.:|||:||||||||||||||||:.|:|:||||||||:|||
Aplysia               543 LCKRFLLTGVPEGLVSDKQVRVSLKSLALGCVAHSVAICPRLFLFKVCPT    592

Lymnaea               597 ANNAGNDQNLQDTTLYASHPDHQLKGQTAVVIGSFIRAALIEGRGNFHQW    646
                          |..||:||||||.||||.|.|||||||||:|:||||||||:|||.:|.||
Aplysia               593 ATGAGSDQNLQDLTLYAGHSDHQLKGQTALVLGSFIRAALLEGRADFSQW    642

Lymnaea               647 IDSHKPPEQSVLSLEALLKIIINILEDESAVAVRAALMALQMCLSYLMDS    696
                          :..|.|.||:|||||.||:||:|||.|||||||||.|:|||||||:|:||
Aplysia               643 VMEHLPSEQNVLSLEDLLRIIVNILGDESAVAVRAGLVALQMCLSHLLDS    692

Lymnaea               697 CHGRLGFRILLDLLIVKTNPYWLVKVELLELIAGLNFKVISYLESISPDI    746
                          ||||||..||||||.:..|||||||||||||:||||||||||:|||||||
Aplysia               693 CHGRLGLSILLDLLCIHNNPYWLVKVELLELMAGLNFKVISYMESISPDI    742

Lymnaea               747 ARGDHNFLGRMCLQEHIFQEIVIQLLGDEDPRVRAAASAAILRMVPTLFF    796
                          .||:||:||:||||:|..|||::.||||||.|||.||:.|.||::|.||:
Aplysia               743 TRGEHNYLGKMCLQQHCIQEILLPLLGDEDARVRTAAANAFLRLIPKLFY    792

Lymnaea               797 GSDSPQQDPVLSVASDLTQHLLTPIMNPIMAGQLPPLVQGLMKPYAFDIL    846
                          .||||||||||:||||.|::||||:|:|:::||||||||||:.||..|.|
Aplysia               793 ASDSPQQDPVLAVASDQTRYLLTPMMSPVLSGQLPPLVQGLVGPYKHDEL    842

Lymnaea               847 QEIDPSTESALSRVVQQLLHTLLMSQSKFVTSGCCLALCRLSEEYLVTQY    896
                          .::.|:||||||||||.|||.||:|.|:::|||||.||||||||||||||
Aplysia               843 AQVCPATESALSRVVQMLLHNLLVSNSRYITSGCCNALCRLSEEYLVTQY    892

Lymnaea               897 ASSWSCGPAKPIASKERVEKLGMRRPPSRSLSASSMDELTSASGGGPLPI    946
                          |:||.|||||||..||..|:.|:|||||||||||||||||:|||||||||
Aplysia               893 AASWGCGPAKPIVPKESEERFGIRRPPSRSLSASSMDELTAASGGGPLPI    942

Lymnaea               947 VLSLMLSSQAGLELTTHQDLLELAGNLVCGAAYKNLRPSEDTEKLSGSGD    996
                          |:|||||||||||||||||||:||||||.|||||||:.|::.:|.  |||
Aplysia               943 VMSLMLSSQAGLELTTHQDLLQLAGNLVAGAAYKNLKNSDELDKT--SGD    990

Lymnaea               997 DGHWAAVADRFLVPMIVQLFTHTARLLNACTHAIEETMPGPPQVKPSLPS   1046
                          |..|:||:||.|:|:|.||||||||||||||||||||.||||||||||||
Aplysia               991 DVSWSAVSDRLLLPIIEQLFTHTARLLNACTHAIEETNPGPPQVKPSLPS   1040

Lymnaea              1047 LPNAATLSPVRRKMKGEKETNPPGPGASPDQKSGQKTPGKDQKDSEKDRN   1096
                          ||||:|||||||||||||:.:|..|..|||.|:||||||||.||||||:
Aplysia              1041 LPNASTLSPVRRKMKGEKDASPQAPVGSPDPKTGQKTPGKDSKDSEKDK-   1089

Lymnaea              1097 RKDGIGSFYNIPQYVKLFEVLRGSYSNFKTSLDLTSSDKFCTMLRTTLTV   1146
                          :|||||:|||||||:|||:|||||||||||||||||||||||:||:.|.|
Aplysia              1090 KKDGIGAFYNIPQYMKLFDVLRGSYSNFKTSLDLTSSDKFCTILRSALRV   1139
```

```
Lymnaea        1147 LSQLLEIATLYDVGKVTDEILGYLKVTMALEPTWTVLCVQQLLKALFGTN   1196
                    ||||||:|||.||||||:|:||||||||||||||||||||||||||||||
Aplysia        1140 LSQLLEVATLQDVGKVTEEMLGYLKVTMALEPTWTVLCVQQLLKALFGTN   1189

Lymnaea        1197 MASQWDSNSQSATSLTSDLGARVAGGSSPGLYYYCLNKPYAQLAHCLVGA   1246
                    :||||:.:.|||..:.:::..||..|:|||||::|||:||||||||||.|
Aplysia        1190 LASQWEPSKQSAAPILAEVSTRVTAGTSPGLYHHCLNRPYAQLAHCLVAA   1239

Lymnaea        1247 ACRATLPADEAHGSLLWLKQRVDRKLPAILKPTSKVDKAVIGSYIRLFEP   1296
                    ||||||||||||.|||||||||||||||||||:|||||:|||||||||||
Aplysia        1240 ACRATLPADEAHSSLLWLKQRVDRKLPAILKPSSKVDKSVIGSYIRLFEP   1289

Lymnaea        1297 LVIKALKQYTVTSSLDLQCQVLALLAQLIQLRVNYCLLDSDQIFIGFVIK   1346
                    ||||:||||||||||:||||||||||||||||||||||||||||||||:|
Aplysia        1290 LVIKSLKQYTVTSSLELQCQVLALLAQLIQLRVNYCLLDSDQIFIGFVLK   1339

Lymnaea        1347 QFEYIEEGQIRNSEVLIPHIFQFLVMLSYEKFHTKSIIDMPRIIHRCDGI   1396
                    ||||||||||||||:||||||||||||||||||:||||||||||||||||
Aplysia        1340 QFEYIEEGQIRNSELLIPHIFQFLVMLSYEKFHSKSIIDMPRIIHRCDGI   1389

Lymnaea        1397 MASGLQPTTHAIPALRPVVYDLFLLRGTVKSEVGKDLETQREVVVSMLLR   1446
                    ||||||||||||||||||||:|||||||||||||||||||||||||||||
Aplysia        1390 MASGLQPTTHAIPALRPVVYELFLLRGTVKSEVGKDLETQREVVVSMLLR   1439

Lymnaea        1447 LVQYYQALDMFTLVLQQCHRESEERWKRLSRQVMDAVLPALAKQQINLES   1496
                    |||||||:|||.|||||||||||||||||||||||.||||||||.:||.|
Aplysia        1440 LVQYYQAVDMFVLVLQQCHRESEERWKRLSRQVMDVVLPALAKQTMNLCS   1489

Lymnaea        1497 QDALDVLHRLFESVSPSVFRPVDFLLKTLLAPPHDVSDVSSLQKWLCLVL   1546
                    |::||||||||||||||||||||.|||||||||||:|.|.|||||:||||
Aplysia        1490 QESLDVLHRLFESVSPSVFRPVDTLLKTLLAPPHDISTVQSLQKWMCLVL   1539

Lymnaea        1547 IIVRVLISQSKEEVILSRLTELQLHVCLIRDLDNSGTSAANSVVELLRNL   1596
                    .|||||||||||:||||||:|||||||||||:||:.::|.:|::|:|:||
Aplysia        1540 TIVRVLISQSKEDVILSRLSELQLHVCLIRDVDNTDSAATSSMIEVLKNL   1589

Lymnaea        1597 APEETVAWFLLQVIGKCSEMLNKETSLLGSNYNGPSYSTSNGNGIIVSRC   1646
                    |||||:||:||||||||||||:|||:|| |:..|.....|:|:|  .||.
Aplysia        1590 APEETLAWYLLQVIGKCSEMLDKETALL-SSAGGGGAGGSSGDG--ESRY   1636

Lymnaea        1647 HFLLQQTLHLMLYITHMFQSGSLFRRVATAAMGLLQLDSPACMYSVGEIN   1696
                    ||||||||||:|||||||||| |||||.||||.||||:||||:|||.:||
Aplysia        1637 HFLLQQTLHLLLYITHMFQSG-LFRRVVTAAMRLLQLESPACVYSVRQIN   1685

Lymnaea        1697 EHMVGVSSVCPALTLHWCNVLILLNFDDRGLWTRVVQSPPTYK-------   1739
                    :|||.||||||.||||||.||||||||||.||:.|||:|||||
Aplysia        1686 QHMVAVSSVCPTLTLHWCYVLILLNFDDRSLWSTVVQTPPTYKVMAHRSH   1735

Lymnaea        1740 ----ST---GTSVRHLPRSSECCGLEMLRRGGLILFCDYVCENLSDAEHM   1782
                        ||   ..::....|||:|||||||||||||||||||||||||||||
Aplysia        1736 QYSQSTEDHDATLTPQQRSSQCCGLEMLRRGGLILFCDYVCENLSDAEHM   1785

Lymnaea        1783 TWLIINHVSDLILLSNESPVQDFIRQVAIHRNSAASSLFIQAIHARGDSI   1832
                    |||||||||||||||.||||||||.  |||||.|||||||||||||||||
Aplysia        1786 TWLIINHVSDLILLSQESPVQDFIG--AIHRNPAASSLFIQAIHARGDSI   1833

Lymnaea        1833 TKPSMVKRTLKCLDAIHISQSGSLVALLIDKFLGCHRLAITRMTDSIVCQ   1882
                    |||.:|||||:||:|:|.||||||||||:|.|||.|:|||.|:||:|||.
Aplysia        1834 TKPHLVKRTLRCLEAVHSSQSGSLVALLVDTFLGSHQLAIARLTDAIVCH   1883

Lymnaea        1883 RLESLLGETAEEIAKQLPKEDIEKQLQFMKSNGLIQRHQRLASLLSKLCI   1932
                    |||:||||||.||.||||||||||.||||||.|||||||||.||||||..
Aplysia        1884 RLETLLGETAGEITKQLPKEDIEKLLQFMKSKGLIQRHQRLTSLLSKLGT   1933
```

```
Lymnaea         1933 AAGSTAQIKLSPERSHPLSVVPVDVAKISIDKEFYLSIVKEQCFMATPNT   1982
                     |||||:|::||||||:|||....:||.::|||||||::||||||.|:||.
Aplysia         1934 AAGSTSQVRLSPERSNPLSGPVTNVANVTIDKEFYLAVVKEQCFQASPNI   1983

Lymnaea         1983 RECAFLLQRLDYPDILSITMTKEFNLSILEECMSLGAFRSVLRYNRDVDL   2032
                     ||||||||||:||||||||||.|||||:|||||||||||||||||||.:.
Aplysia         1984 RECAFLLQRLEYPDILSITMTSEFNLSVLEECMSLGAFRSVLRYNRDAEA   2033

Lymnaea         2033 GSLSPQPASNEHTLDPLFQASQLTLFRHINNMVIQLPLPHQCLVFIDSAP   2082
                     |:|||..|..|..:||||.||:|||||||||:|.|||.|||||.|.|..|
Aplysia         2034 GTLSPLAAPGERIMDPLFLASELTLFRHINNVVNQLPTPHQCLSFTDCGP   2083

Lymnaea         2083 ASSLHYMDRIEELFTDAQWVDNNFCLASALVRYMVAIHQFPWRAELPPES   2132
                     |||||||||:|||||||.||..||.||:||||::||:..||||||||.:|
Aplysia         2084 ASSLHYMDRMEELFTDAAWVHANFNLAAALVRHLVALSHFPWRAELPADS   2133

Lymnaea         2133 LKDVASFVVLCAELIHWSVEHDMLPESEHIQNCLSCLSLLLQDPAVHALM   2182
                     ..|||.|.||||||||||:.|:::||||.||.|||||||||||..:|:.:
Aplysia         2134 HHDVARFAVLCAELIHWSLRHELMPESEQIQTCLSCLSLLLQDGKLHSQL   2183

Lymnaea         2183 SQTEHATFVCSIVGFLYQLLCSMAVLPGENVACLFQDDRRDDAEEEDVSL   2232
                     .|.|:|:.||:||..|||:|.|:||||||.||.|:.|:.|::|||:||||
Aplysia         2184 GQIENASCVCAIVAALYQVLSSLAVLPGEQVANLYHDEHREEAEEDDVSL   2233

Lymnaea         2233 SACLIRACDEISELVHCLHTRLDPNTSHEPRLPQFLASTFRNIIIAVARL   2282
                     ||||:|||::|||||||||||:||:.:.|||||.||||:||||:|:|:||
Aplysia         2234 SACLVRACEQISELVHCLHTRMDPSYTKEPRLPPFLASSFRNIVISVSRL   2283

Lymnaea         2283 PAVNTYARTPPLVWRLGWSPTPVGELRTCLPPLPVEYLQEKDVLKEFVTR   2332
                     |.||||||||||||||||||||.|||||||||||||||||||||:|||.|
Aplysia         2284 PVVNTYARTPPLVWRLGWSPTPGGELRTCLPPLPVEYLQEKDVLREFVAR   2333

Lymnaea         2333 ISSLGWVNRQQFEESWMSLLGVLNPVSHTGHDLSAEEEIEQAQGMVIAVK   2382
                     |:.|||||||||||||||||||||||||||||||.|||||||||||:|||
Aplysia         2334 INFLGWVNRQQFEESWMSLLGVLNPVSHTGHDLSPEEEIEQAQGMVLAVK   2383

Lymnaea         2383 AITSLLLQSSMVPHAGNPSNSYYETRPRDKPLAFLHTRCGKKLSVIRGLI   2432
                     ||||||||:|:||.:||.:||.||||||||||||||||||||||||||||
Aplysia         2384 AITSLLLQASLVPTSGNTANSCYETRPRDKPLAFLHTRCGKKLSVIRGLI   2433

Lymnaea         2433 EKEIVNLCAARPDRLVPQAYSGSPSDKSSPNLFDGNLERELGVEDFSLGQ   2482
                     |:||:||||:||||||.|||.|:||||:|.:||:||||||:|.|::||||
Aplysia         2434 EQEIINLCASRPDRLVAQAYCGNPSDKASVSLFNGNLEREIGSEEYSLGQ   2483

Lymnaea         2483 ISIESTWSLVGSLDTNLSNSDTTDSLDSPTGRGDMAATSPSSASSSSG-G   2531
                     |||||||||||:||..||.|::|||.||..|:. .|.|:||:.|.||| |
Aplysia         2484 ISIESTWSLVGNLDLPLSTSESTDSPDSTAGQA-AAETTPSATSPSSGSG   2532

Lymnaea         2532 RLS-----TARSVHHCGLDIHSCLQFLLELYGAWLHIDNNPKPPLMLLNS   2576
                     ..|     ::|||..|||||||||||||||||||||:||:||||||||||
Aplysia         2533 SASSPEGVSSRSVSRCGLDIHSCLQFLLELYGAWLHVDNSPKPPLMLLNS   2582

Lymnaea         2577 VVKSMVCLSDLFMEREQFEFMQDILLDLLKGHPVEDELITQYLIVGMCKA   2626
                     ||||||.|||||:||||||||||||||||||||.||||:||||:||:||:
Aplysia         2583 VVKSMVSLSDLFVEREQFEFMQDILLDLLKGHPAEDELVTQYLVVGLCKS   2632

Lymnaea         2627 TAIVGTEALISERVVKLIESGLKSTHLPTKISSLHGALYLLEGGPSELNS   2676
                     ||:||||||::||:|||:||||||||||||||:||||||:||.|.|:|::
Aplysia         2633 TAVVGTEALVNERMVKLVESGLKSTHLPTKISALHGALYVLETGVSDLST   2682
```

```
Lymnaea         2677 SLLPILTDFLSKHLAIVISQTCIISQQFVIIMWAVAFYIIENFSNELKDS   2726
                     :.||:|||||:|||||| ||.||||||||:.||||||||||||:||:||.
Aplysia         2683 AFLPVLTDFLAKHLAIV-SQVCIISQQFVVNMWAVAFYIIENFTNEIKDL   2731

Lymnaea         2727 DFTSKTIQLVVQTASGNEENVSTSVFLTVMKGTERLLLIDVLTQSDTETI   2776
                     ||:||.:.||||||||.||.|||:|||.||||||||||.|||||:|||||
Aplysia         2732 DFSSKIMPLVVQTASGTEETVSTAVFLVVMKGTERLLLTDVLTQTDTETI   2781

Lymnaea         2777 IKLSMDRLCLPNPQRALAALGLMFTCMYSGKSTDQYSPQPREEQMFGDSG   2826
                     ||||||||||||||||||||||||||||||||.|||||:|||||.||:.|
Aplysia         2782 IKLSMDRLCLPNPQRALAALGLMFTCMYSGKSVDQYSPRPREEQTFGEPG   2831

Lymnaea         2827 FQLLHQDPDSLILAMERVTVLFDRIKKGYPYEARVITRLLPAFLADFFPA   2876
                     |:||||||||||||||||||||||||||||||||||||||||||||||||
Aplysia         2832 FELLHQDPDSLILAMERVTVLFDRIKKGYPYEARVITRLLPAFLADFFPA   2881

Lymnaea         2877 QDIMNKVIGEFLSAHQPYPYLIAKVVFQVFTNLHQQKQQGLVKEWVMLSL   2926
                     ||||||||||||||||||||||||||||||..||||:||.||:|||||||
Aplysia         2882 QDIMNKVIGEFLSAHQPYPYLIAKVVFQVFATLHQQQQQSLVREWVMLSL   2931

Lymnaea         2927 SNFTQRSPVSMAMWSLTLFFISASTNVWLRALFPHVLGRMGYMEPMDRRL   2976
                     ||||||||.:||||||:|||||||||:||||||||||||||:||.:||:|
Aplysia         2932 SNFTQRSPPAMAMWSLSLFFISASTNIWLRALFPHVLGRMGHMESLDRQL   2981

Lymnaea         2977 FCLCAVDFYCQLTDDGHKRAFLATFQTIAAPDSPYSDLVQCITML   3021
                     |||||:|||.|||::|.:||||:|||.||||:|||||||.|:.
Aplysia         2982 FCLCALDFYRQLTEEGQRRAFLSTFQAIAAPESPYSDLVACLA--   3024


#---------------------------------------
#---------------------------------------
```

#**Parkinson disease protein 7/Protein deglycase DJ-1**

```
#
# Aligned_sequences: 2
# 1: Lymnaea stagnalis
# 2: Aplysia californica
# Matrix: EBLOSUM62
# Gap_penalty: 10.0
# Extend_penalty: 0.5
#
# Length: 209
# Identity:     132/209 (63.2%)
# Similarity:   154/209 (73.7%)
# Gaps:          25/209 (12.0%)
# Score: 670.0
#
#
#=======================================

Lymnaea            1 MFNLCCNKAGHRLFYHSSRKNMASVSTALVFLAEGAEEMETVITVDVLRR     50
                                             :.:||||||||||||||||:||||||
Aplysia            1 ------------------------MPSALVFLAEGAEEMETVISVDVLRR     26

Lymnaea           51 GEVDVVLAGIDGDGPVKCSRNVKLVPDKSLRDALH-KEYDVLICPGGGLG     99
                     ..:||.|||:.|||||.|||||:|||||:|:.||. ..||||:.||||.|
Aplysia           27 AGIDVTLAGLGGDGPVLCSRNVRLVPDKNLKQALSAAPYDVLVLPGGGGG     76

Lymnaea          100 AENLCKSKEVGKALQEQEKRGGFIAAICAGPTALLAHNVGKGKKITSYPS    149
                     ::.|.:|:||.|||.|||.|||:|||:||.|||||||.:.||||:||:||
Aplysia           77 SKKLSESEEVRKALLEQENRGGLIAAVCAAPTALLAHGIAKGKKVTSHPS    126
```

```
Lymnaea          150 MADKLKDAYKYSEDRVVVDGKFITSRGPGTCFEFALAIVEQLKGKEKAHA    199
                     :|||...|.|||||||||||.||||||||||||||||.:|.|||||.:
Aplysia          127 VADKLSGDYSYSEDRVVVDGKLITSRGPGTCFEFALAIVTELMGKEKAES    176

Lymnaea          200 LVKPMLIKI    208
                     ||.|||:|:
Aplysia          177 LVDPMLVKL    185


#---------------------------------------
#---------------------------------------
```


#**Ubiquitin-protein ligase E3A**

```
#
# Aligned_sequences: 2
# 1: Lymnaea stagnalis
# 2: Aplysia californica
# Matrix: EBLOSUM62
# Gap_penalty: 10.0
# Extend_penalty: 0.5
#
# Length: 927
# Identity:     638/927 (68.8%)
# Similarity:   749/927 (80.8%)
# Gaps:          39/927 ( 4.2%)
# Score: 3274.0
#
#=======================================

Lymnaea            1 MSSIQNTGSLDGQESASDLTVEHSLSGKVPKVSESTENMSKRGAAQQVIF     50
                     |||.....|||||:|||||:||:.::   .::|...||| ||.||:|:|.
Aplysia            1 MSSSNTNSSLDGQQSASDLSVENLIN---RRLSPPPENM-KRAAAKQLIE     46

Lymnaea           51 QYFNQLTEGCGNESCTNENCASSRTFRFKESDNNTLALEAITLSKAKATL    100
                     ||:.||||||:|:|||||||||..|:|::::.|.||||||.|||:||.|
Aplysia           47 QYYYQLTEGCGDENCTNENCASSSDFQFRDANRNKLALEAINLSKSKAPL     96

Lymnaea          101 CERRPRKISRLPTEQENGTLDTSVLVASKELGMRTKSKMLSPTA-SSSVD    149
                     |||||.|:||.||::|.....::...|.:.:|...:||:.|||: |||.|
Aplysia           97 CERRPNKLSRYPTQEEGAASSSTAASAGEPVGQGARSKIASPTSCSSSTD    146

Lymnaea          150 VG-------SSQSPHGSNSEIKEPNYLTEERLSAVIHDCQASNEWLKLIH    192
                     |.       ||.|..|:.::     :||||.|:.||.:|:|.|.|.||.|
Aplysia          147 VAAEAVSACSSSSSLGTATQ-----FLTEEILTKVIDECEARNSWSKLNH    191

Lymnaea          193 LIGSVFNNPESIIMSFRKSYPVSSPPKVNLVSPDDSSSP-----------    231
                     :|||||||||||::|||||:.|.|.|......:.|:||:.
Aplysia          192 VIGSVFNNPESILLSFRKASPASEPVVDEAAATDESSATTSQASDGDSSG    241

Lymnaea          232 -----PISQPGIESPTVPTVSSIKDPDLTVDLPALRRAFKLLMDVPDQPY    276
                          |.||.|..|....|.....|.|::||||:||||::.|:||||||:
Aplysia          242 SNLPVPESQSGPASTDKVTTDGHHDDDMSVDLPSLRRAYQRLLDVPDQPF    291

Lymnaea          277 QGALINALTALSQTLEYETKYNKVLERQTDYINVFIVVMEIPMLHFPEYL    326
                     ||||:|||..||.||:.|.||...|.|:.||||||::||||||||:||||
Aplysia          292 QGALVNALMILSHTLDMELKYKDALARRPDYINVFVIVMEIPMLHWPEYL    341

Lymnaea          327 ETAFPGINKVIGLLPVSAQARLARIWSTFDQSRLKDMVQSLQQLITVKVI    376
                     ..|||.:.:.:|.:|:|.||||||||:|:...||::|||.|||:||||||
Aplysia          342 VNAFPCLCRALGSMPLSGQARLARIWATYGSGRLQEMVQGLQQMITVKVI    391
```

```
Lymnaea          377 NYEGRWSANFRPSDDSAITSATRVLKILYYASMLGGHMDSTELVEEERRL    426
                     |.|||||.:|:|.||.:|||||||||::||||:|||||||.||:.||..|
Aplysia          392 NNEGRWSQSFQPGDDMSITSATRVLKVIYYASVLGGHMDSPELLREEAEL    441

Lymnaea          427 NDSESLLELMQGAFGYEPKESSPVKEDPLGKEVGVQVINCREPLIPYEDF    476
                     :..||:.|.||||||.|||:|:|.||||||||:|||||||||||:|:|||
Aplysia          442 SQRESIQEHMQGAFGMEPKDSTPPKEDPLGKELGVQVINCREPLVPFEDF    491

Lymnaea          477 INEPLNDNLDIGVDYTNHRLEPENKFSFVPYCFILTTASKHTSMYYDNRI    526
                     |||||||.::|..||||      ||||||||.|||||||||:||||:|||
Aplysia          492 INEPLNDTVNIYQDYTN------NKFSFVPYSFILTTASKHSSMYYENRI    535

Lymnaea          527 RMLHERRTAFVQTLVHGGPPNPFLRVRVRRDHIIDDALVNLEMIAMENPS    576
                     |||.||||||:||||.|||||||||||||||:||||||||:|||.||||||
Aplysia          536 RMLSERRTAFIQTLVSGGPPNPFLRVRVRREHIIDDALVSLEMTAMENPS    585

Lymnaea          577 DLRKQLFVEFDGEQGLDEGGVSKEFFQLIVEELFNPDIGMFTYNEQSHHF    626
                     ||||||||||:||||||||||||||||||||||||||||||.|||:||||
Aplysia          586 DLRKQLFVEFEGEQGLDEGGVSKEFFQLIVEELFNPDIGMFAYNEESHHF    635

Lymnaea          627 WFNSLSFENDAQFTLIGILLGLAIYNSCILDIHFPMVVYRKLMGKKGTFR    676
                     |||||||||||||||||||||||||||||||||||||||||||||||||.
Aplysia          636 WFNSLSFENDAQFTLIGILLGLAIYNSCILDIHFPMVVYRKLMGKKGTFA    685

Lymnaea          677 DLYDVDPTLFASLKEMLEYKLEDFEEVFDQTFRIGYSDVFGNNHTYDLKE    726
                     ||||:||||..||::||:|:.||||:||.|:|.|||.||||:.||.:|||
Aplysia          686 DLYDLDPTLMHSLQDMLDYEGEDFEDVFAQSFSIGYHDVFGHCHTVELKE    735

Lymnaea          727 NGESTMVSQENKQEFVDLYADYLLNKSIDQQFRAFKRGFLMVTSESPLKQ    776
                     .|||..::|:||.||:.|||||||||||::||.|||||||||||||||:|
Aplysia          736 GGESLPLTQDNKHEFIKLYADYLLNKSIERQFLAFKRGFLMVTSESPLRQ    785

Lymnaea          777 LFRPEEIEMLVCGSQIFDFHALEEATEYDGGFTDDSATIRNFWTVVHAMS    826
                     ||||||||:|||||:|.|||||||||||||||.:|.||||||||||.|.
Aplysia          786 LFRPEEIEVLVCGSKILDFHALEEATEYDGGFTQESQTIRNFWTVVHDME    835

Lymnaea          827 EEDKKKLLQFTTGTDRVPVGGLSKLKMIIARNGPDSDRLPTSHTCFNVLL    876
                     |.:|||||||||||||||||||.||||||:||||||||||||||||||||
Aplysia          836 ETEKKKLLQFTTGTDRVPVGGLSHLKMIIAKNGPDSDRLPTSHTCFNVLL    885

Lymnaea          877 LPEYPTVEKLQDRLLKAINYSKGFGML    903
                     |||||:||||:||||||||||||||||
Aplysia          886 LPEYPSVEKLKDRLLKAINYSKGFGML    912


#---------------------------------------
#---------------------------------------
```

#**Major vault 1**

```
#
# Aligned_sequences: 2
# 1: Lymnaea stagnalis
# 2: Aplysia californica
# Matrix: EBLOSUM62
# Gap_penalty: 10.0
# Extend_penalty: 0.5
#
```

```
# Length: 872
# Identity:     658/872 (75.5%)
# Similarity:   749/872 (85.9%)
# Gaps:          21/872 ( 2.4%)
# Score: 3407.5
#
#
#=======================================

Lymnaea            1 MADPRKTRSDDPHSRWDKADNPIYRIPPYYYIHVMDQNSNVTRIELGPQT     50
                                ..||  |.||.|:||||||||||:|||:||||||:||||
Aplysia            1 -----------MTSR--KEDNAIFRIPPYYYIHVLDQNTNVTRIEIGPQT     37

Lymnaea           51 FVRQDNQRVTLGPEKMIIVPPRHYCTIENPVCRDKDGNVVVDRLGQIKLK    100
                     |:||||||||||||||::|||||||.|||||.|||||..|:|:|||.||:
Aplysia           38 FIRQDNQRVTLGPEKMVVVPPRHYCMIENPVFRDKDGKPVIDQLGQTKLQ     87

Lymnaea          101 HAELEIRLAQDPFPLYPGEILRHPVTALTVVQSNSALKILAVLDFKDGDV    150
                     ||:|||||.||||||:|||:|:.|||||.||.:|:||:|.||||::|.:.
Aplysia           88 HADLEIRLTQDPFPLFPGEVLKQPVTALKVVPANAALRIRAVLDYEDAEG    137

Lymnaea          151 D-RAAGDVWLFEGPGTYIPKKETVVKETVRATVIGPNQAIKLRATKEFED    199
                     : |.|||.|||||||||||:||.:::||:|||||||||||:|||.||..|
Aplysia          138 EKRTAGDEWLFEGPGTYIPRKECMIEETIRATVIGPNQAIRLRARKECID    187

Lymnaea          200 RDGNLRVTGEEWLVKQTGAYLPGVFEEVVDIVKAYVLTETKALHVRALKS    249
                     ||...||||||||||:|||||||.:|||||:|.||||||.||||:|||.:
Aplysia          188 RDNIRRVTGEEWLVKRTGAYLPGAYEEVVDLVNAYVLTEKKALHMRALMT    237

Lymnaea          250 FKDDFGVERKNGEEWLITMDDSEAHIPGVYEEVVGVVNITTLTNRQYAVI    299
                     ||||||:.|||||||||||.|:|.|||.||||||||||||.|:||||.||
Aplysia          238 FKDDFGIVRKNGEEWLITMTDTETHIPNVYEEVVGVVNITPLSNRQYCVI    287

Lymnaea          300 LDPVGADGKPQLGQKKLVRGEKSFFLLPGEKLEKGIQNIYILGEDEGLVL    349
                     ||||...|:|||||||||:|||||||:|||||||||||:|||||||||:|
Aplysia          288 LDPVDDKGRPQLGQKKLVKGEKSFFLMPGEKLEKGIQNVYILGEDEGLIL    337

Lymnaea          350 RAIEQFKDGVSQRSPGDRWMIRGPLEYVPPVEIEVVMKRKAIPLDLNEGI    399
                     ||.|.|.||...|:|||||||:||.|||||||:|||:||:|||||.||||
Aplysia          338 RANESFMDGQINRNPGDRWMIKGPTEYVPPVEVEVVLKRQAIPLDENEGI    387

Lymnaea          400 YVRDIKTGKVRAITGATYMIKEDEELWEKELPETVEELIISERDPKSERY    449
                     ||||||:||||||.|.||||.:|||||.||||.|||.|:.:.:||.::|.
Aplysia          388 YVRDIKSGKVRAIQGETYMINQDEELWAKELPPTVESLLSAGKDPLADRS    437

Lymnaea          450 ATKGQDSKSKTRDKTRVVTFRVPHNAAVQIYDYKDKKSRVVFGPELVMLG    499
                     ..|..|...|||||||||||||||||||||||||:||:||||||||||||
Aplysia          438 DRKSTDHSPKTRDKTRVVTFRVPHNAAVQIYDYKEKKARVVFGPELVMLG    487

Lymnaea          500 PDEQFTVLSLSGGKPKKNHAIKALCLLLGPDFATDIITVETADHARLSLQ    549
                     ||||||.||||||||||.:.||||||||||||.||||.||||||||||||
Aplysia          488 PDEQFTQLSLSGGKPKKPNLIKALCLLLGPDFCTDIIVVETADHARLSLQ    537

Lymnaea          550 LAYNWYFEVTGKTPEESAKLFSVPDFVGDSCKAIASRVRGAVAQVTFDDF    599
                     |:|||:||...|:|||:||:||||||.||||||||||||||||||.||||
Aplysia          538 LSYNWHFETLSKSPEEAAKIFSVPDFTGDSCKAIASRVRGAVAQVQFDDF    587

Lymnaea          600 HKNSAKIIRSSVFGF-KDGKVGSHLTFSQNNLVITNVDIQSVEPVDQRTR    648
                     ||||||||||||||| .:.||.....|.|||||||::|||||||||||||
Aplysia          588 HKNSAKIIRSSVFGFDPNNKVRDKFVFPQNNLVITSIDIQSVEPVDQRTR    637
```

```
Lymnaea          649 DSLQKSVQLAIEITTNSQEATARHEAERLEQEAKGRLERQKIMDEAEAEK    698
                     |:||||||||||||||||||.|||||||||||||||||||||||||||||
Aplysia          638 DALQKSVQLAIEITTNSQEAAARHEAERLEQEAKGRLERQKIMDEAEAEK    687

Lymnaea          699 SRRELLELQANSAAVESTGQAKAEAQSRAEAARIEGEAAVQQAKLKAEAQ    748
                     :|:|||||||||||||||||||||||||||||:|||||||:||:||::|.
Aplysia          688 ARKELLELQANSAAVESTGQAKAEAQSRAEAAKIEGEAAVEQARLKSQAT    737

Lymnaea          749 TIEAESDLQRLTQARTAELAYLKEQNIMEIEKTTELTTIEVNKFRQMVES    798
                     .||:|::|:|||.||.|||.||.|||.||:||.||::.||..||::|||:
Aplysia          738 KIESEAELERLTSAREAELKYLSEQNRMELEKATEMSNIETTKFKEMVEA    787

Lymnaea          799 IGSGTLLAMATAGPDMQVRMLQALGLKSTLITDGTTPVNLFNTASGLIGD    848
                     ||:.||..||||||||||||||:|||||||||||::|:|||||||||||.
Aplysia          788 IGTQTLQTMATAGPDMQVRMLQSLGLKSTLITDGSSPINLFNTASGLIGG    837

Lymnaea          849 LVPSKRRRREKKEEEEEESDSFA    870
                     |||.:.|.|:..:::|
Aplysia          838 LVPKRHRARDDDDDDDE------    853


#---------------------------------------
#---------------------------------------
```

Table S1. Detailed functions of *L. stagnalis* findings in higher organisms.

***L. stagnalis* findings to genes linked to human aging/disease-related genes**	**Their normal and pathological functions in higher organisms**
klotho [MT153186]	A transmembrane protein mainly involved in the modulation of aging. Mice models revealed that a defect in Klotho gene expression accelerates the aging process, while its overexpression extends the life span.
major vault 1 [MT153187]	Major vault protein is the main component of ubiquitous cellular ribonucleoprotein particles called vaults. It is involved in intracellular signalling and cell survival. Due to its anti-apoptotic effect, it is considered to play a role in aging modulation.
gelsolin [MT153188]	A Ca^{2+}-dependent actin regulatory protein. It has an anti-aging function manifested in an age-related anti-apoptotic effect in mitochondria. Well-characterized point mutations of gelsolin gene will cause unfolded protein conformations and so systematically deposed gelsolin proteins leading to Gelsolin Amyloidosis disease.
huntingtin [MT153189]	The exact function of huntingtin protein is unknown, however, animal models revealed that it is necessary for development, memory consolidation, and it is linked to aging. The increased number (more than 36 copies) of repetitions of the tribasic CAG sequence at the 5' end of huntingtin gene is the main cause of Huntington's disease.
fragile X mental retardation protein [MT153190]	The protein is most commonly found in the brain and essentially responsible for normal cognitive development and female reproductive function. The increased number (more than 200 copies) of repetitions of the tribasic CGG sequence in the gene

	leads to fragile X mental retardation syndrome. Moreover, minor expansions of this repeat that do not cause fragile X syndrome are associated with an increased risk for premature ovarian aging.
Parkinson disease protein 7/Protein deglycase DJ-1 (PARK7/DJ-1) [MT153192]	PARK7/DJ-1 protein has a neuroprotection function acting as a chaperon to inhibit the aggregation of α-synuclein. Defect of the gene causes autosomal recessive early-onset Parkinson's disease.
α-secretase (ADAM10) [MT153191]	α-secretases are proteolytic enzymes that cleave APP in its transmembrane region. Since the α-secretase pathway is the predominant in APP processing, mutations can lead to β-amyloid production and so to Alzheimer's disease.
apolipoprotein E (apoE) receptor [MT137053]	Being involved in different neural signalling pathways, these receptors seem to play a protective role against β-amyloid production and tau phosphorylation.
choline acetyltransferase (ChAT) [MT153193]	A transferase enzyme responsible for the synthesis of neurotransmitter acetylcholine. Disturbances of ChAT and so the cholinergic system is associated with Alzheimer's disease.
amyloid precursor protein (APP) [MT153194]	An integral membrane protein expressed in many tissues and concentrated in the synapses of neurons. It is primary implicated as a regulator of synapse formation. Mutations of the protein as well as β-amyloid production from APP by secretases is one of the main cause of Alzheimer's disease.
presenilin 1 (PSEN1) [MT153195]	PSEN1 is a transmembrane protein which constitutes the catalytic subunits of the gamma-secretase intramembrane protease complex. Mutations of the gene or protein cause early onset forms of familial Alzheimer's disease manifested in, for example, an increased ratio of produced Aβ 42 / Aβ 40.
notch receptor 3 [MT153197]	Being as a part of notch signalling pathway, the receptor plays a key role in neuronal development. Mutations in the gene have been identified as the underlying cause of cerebral arteriopathy.
potassium voltage-gated channel subfamily KQT member 2 isoform e (KCNQ2) [MT153198]	Forming the so-called M channel by association with KCNQ3, KCNQ2 plays a critical role in the regulation of neuronal excitability. Defects of this gene cause epilepsy.
aldehyde dehydrogenase family 3 member A2 isoform 2 (ALDH3A2) [MT153199]	An aldehyde dehydrogenase enzyme what plays a major role in the detoxification of aldehydes generated by alcohol metabolism and lipid peroxidation. Mutations of the gene will lead to Sjörger-Larsson syndrome.
copper-transporting ATPase 2 isoform a (ATP7B) [MT153200]	This protein is a member of the P-type ATPase family, a group of proteins that transport metals into and out of cells by using ATP. Mutations of the gene, manifested in protein structure changes what prevent copper-transporting, cause Wilson disease.
ubiquitin-protein ligase E3A (UBE3A) [MT153201]	Attaching ubiquitin as a marker to proteins that should be degraded, this enzyme is involved in targeting proteins for degradation within cells.

	Mutations of the gene, mostly resulted in non-functional proteins, are responsible for Angelman syndrome.
